# Identification of Pro-Inflammatory Cytokines Associated with Muscle Invasive Bladder Cancer; The Roles of IL-5, IL-20, and IL-28A

**DOI:** 10.1371/journal.pone.0040267

**Published:** 2012-09-04

**Authors:** Se-Jung Lee, Eo-Jin Lee, Seon-Kyu Kim, Pildu Jeong, Young-Hwa Cho, Seok Joong Yun, Sangtae Kim, Gi-Young Kim, Yung Hyun Choi, Eun-Jong Cha, Wun-Jae Kim, Sung-Kwon Moon

**Affiliations:** 1 Department of Biotechnology, Chungju National University, Chungju, Chungbuk, South Korea; 2 Department of Food Science and Technology, Chung-Ang University, Ansung, Korea; 3 Juseong Gene Therapy R&D Center, Juseong University, Chungbuk, Korea; 4 Laboratory of Immunobiology, Department of Marine Life Sciences, Jeju National University, Jeju, Republic of Korea; 5 Department of Biochemistry, College of Oriental Medicine, Dongeui University, Busan, South Korea; 6 Department of Urology, College of Medicine, Chungbuk National University, Cheongju, Chungbuk, South Korea; 7 Korean Bioinformation Center, Korea Research Institute of Bioscience and Biotechnology, Daejeon, Korea; 8 Department of Biomedical Engineering, Chungbuk National University, Cheongju, Korea; Albert Einstein College of Medicine, United States of America

## Abstract

We used gene expression profiling to identify inflammatory cytokines that correlate with bladder cancer development. Gene expression profiles of the tissue samples were investigated using cDNA microarrays that contained 103 non-muscle invasive bladder cancers (NMIBC), 62 muscle invasive bladder cancers (MIBC), 58 samples of histologically normal-looking surrounding tissues, and 10 normal, healthy subjects who served as the control cohort for comparison. We grouped the data-sets according to biological characterizations and focused on immune response genes with at least 2-fold differential expression in MIBC vs. controls. The experimental data-set identified 36 immune-related genes that were significantly altered in MIBC samples. In addition, 10 genes were up-regulated and 26 genes were down-regulated in MIBC samples compared with the normal tissues. Among the 10 up-regulated molecules examined, the capacity for both wound-healing migration and invasion was enhanced in response to IL-5, IL-20, and IL-28A in bladder cancer cell lines (253J and EJ cells), compared with untreated cells. The expression levels of IL-5, IL-20, and IL-28A were increased in patients with MIBC. All 3 cytokines and their receptors were produced in bladder cancer cell lines, as determined by real-time PCR, immunoblot analysis and confocal immunofluorescence**.** Up-regulation of MMP-2 and MMP-9 was found after IL-5, IL-20, and IL-28A stimulation in both cell types. Moreover, an EMSA assay showed that treatment with IL-5, IL-20, and IL-28A induced activation of the transcription factors NF-κB and AP-1 that regulate the MMP-9 promoter. Finally, activation of MAPK and Jak-Stat signaling was observed after the addition of IL-5, IL-20, and IL-28A to bladder cancer cells. This study suggests the presence of specific inflammatory cytokine (IL-5, IL-20, and IL-28A)-mediated association in bladder cancer development. All 3 cytokines may be important new molecular targets for the modulation of migration and invasion in bladder cancer.

## Introduction

Bladder cancer is one of the most prevalent malignancies in economically advanced countries, and nearly all malignant bladder cancers are transitional cell carcinoma (TCC), which arise from the transitional epithelium [1], [2]. Two types of TCC have been histopathologically classified: non-muscle invasive bladder cancer (NMIBC) and muscle invasive bladder cancer (MIBC) [3], [4]. At initial presentation, 70–80% of patients are diagnosed with NMIBC that is restricted to the mucosa. The remainder of the cases presents MIBC with invasion of the muscular layers of the bladder. The patients with NMIBC can be successfully treated, while the most deaths occur in patients with incident MIBC [4]. Therefore, much effort has been focused on understanding the mechanisms of MIBC development for possible therapeutic applications.

It is now widely accepted that intravesical immunotherapy with Bacillus Calmette Guerin (BCG) is the most effective adjuvant agent for the treatment of NMIBC [5]–[7]. However, the most useful therapeutic method for the treatment of the patients with MIBC remains to be identified. Therefore, many studies have been conducted in order to gain more insight into the mechanisms of MIBC development, which may lead to the discovery of potential therapeutic treatment. The biochemical and biological studies associated with aggressive TCC have been analyzed to determine prognostic indicators, or to develop agents for diagnostic and therapeutic application. Several specific molecular markers have been identified by gene expression profiles in bladder cancer, including cell cycle regulators, cell proliferation, apoptosis and angiogenesis factors [8]. Inflammation is involved in the development of several diseases, such as atherosclerosis, diabetes, and tumors, and is accompanied by the appearance of numerous inflammatory biomarkers [9]–[11]. However, the inflammatory-phenotype association that regulates bladder cancer development and metastasis is still poorly understood.

A huge amount of data has shown that interleukins exert numerous functions by regulating cell growth, cell survival, differentiation, and apoptosis in several diseases [11]. Interleukins may also exhibit distinct effects on the regulation of immune responses and the pathogenesis of bladder cancer [4]–[7]. Treatment with interleukin-6 (IL-6) was associated with anti-tumor effect via the induction of apoptosis in mouse bladder carcinoma [12]. IL-15 gene delivery showed anti-tumor effect in a mouse orthotopic bladder cancer model [13]. In contrast, IL-6 treatment has been found to contribute to cell growth in human bladder cancer cells *in vitro* [14]. Furthermore, the expression of IL-8 and IL-17 has been implicated in tumor growth and metastasis in human bladder cancer [15], [16]. Although many studies have analyzed the effects of interleukins on the growth of bladder cancer [12]–[16], its exact role and molecular mechanism in the process of migration and invasion of TCC associated with clinical study has not been elucidated.

In this study, we have utilized a microarray-based approach to identify clinically and biologically informative expression patterns that differ between patients with NMIBC and MIBC and control samples. Our results focused on differences between MIBC and control samples in the expression patterns of genes that play a significant role in the most important cellular processes involved in inflammatory responses. Genes with at least 2-fold differential expression in MIBC vs. controls were identified, and the novel functions and signaling pathways in an inflammatory-based series of bladder TCC were elucidated.

## Results

### Differential Gene Expression Patterns Among Patients Groups

**Figure 1:**
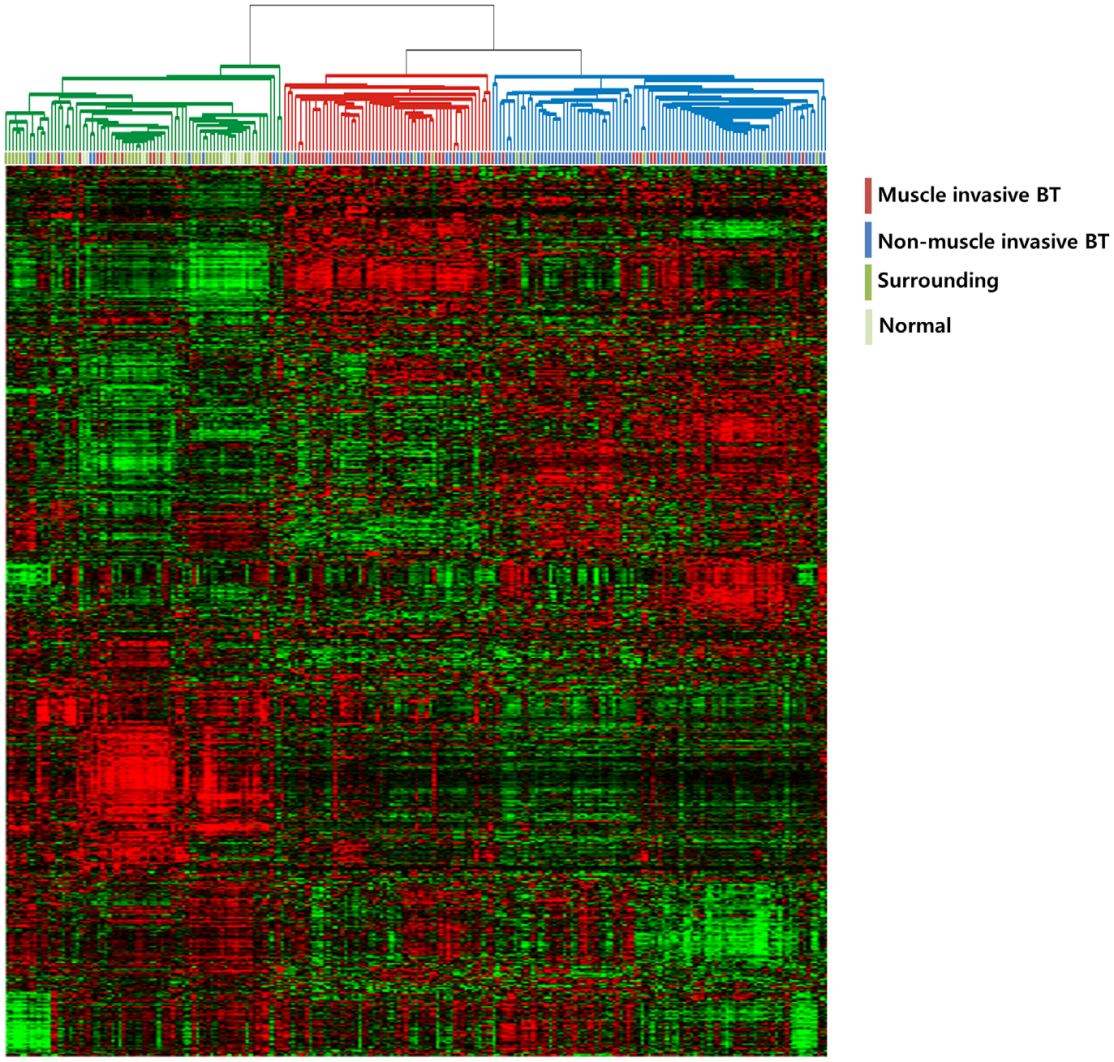
Gene expression patterns of human bladder cancers. Genes with expression values that had a standard deviation of at least 0.7 were selected (4,145 genes). The red and green colors reflect high and low expression levels, respectively.

We characterized the gene expression patterns of 233 bladder cancer patients’ samples; 103 NMIBCs, 62 MIBCs, and 68 normal mucosa or mucosa surrounding (adjacent to) cancers (Table 1). We first applied hierarchical clustering analysis of gene expression patterns to assess the molecular characteristics of the different patient groups. As expected, hierarchical clustering analysis of gene expression data from all tissues yielded 3 major clusters, 1 representing the normal bladder mucosa, 1 representing the MIBC patient group, and 1 representing the NMIBC patient group (Figure 1). Thus, the gene expression patterns reflecting the molecular configuration were readily distinguishable between bladder tumors and non-tumor tissues.

**Table 1:** Baseline Characteristics of Primary Bladder Cancer Patients.

Variables	Characteristics (n = 165)
Age - yr (mean)	65.2±12.0
Gender - no. of patients (%)	
Male	135 (81.8)
Female	30 (18.2)
Grade - no. of patients (%)	
Low	105 (63.6)
High	60 (36.4)
Stage - no. of patients (%)	
NMIBC	103 (62.4)
Ta	23 (22.3)
T1	80 (77.7)
MIBC	62 (37.6)
T2N0M0	26 (41.9)
T3 N0M0	13 (21.0)
T4/Any T N+/M+	23 (37.1)
Recurrence - no. of patients with NMIBC (%)	
No	67 (65.0)
Yes	36 (35.0)
Progression - no. of patients (%)	
NMIBC	
No	92 (89.3)
Yes	11 (10.7)
MIBC	
No	42 (67.7)
Yes	20 (32.3)
Survival - no. of patients with MIBC (%)	
Cancer-specific	
Alive	33 (53.2)
Deceased	29 (46.8)
Overall survival	
Alive	28 (45.2)
Deceased	34 (54.8)
Mean follow-up - months	48.4

Abbreviations: NMIBC, non-muscle invasive bladder cancer; MIBC, muscle invasive bladder cancer.

**Figure 2:**
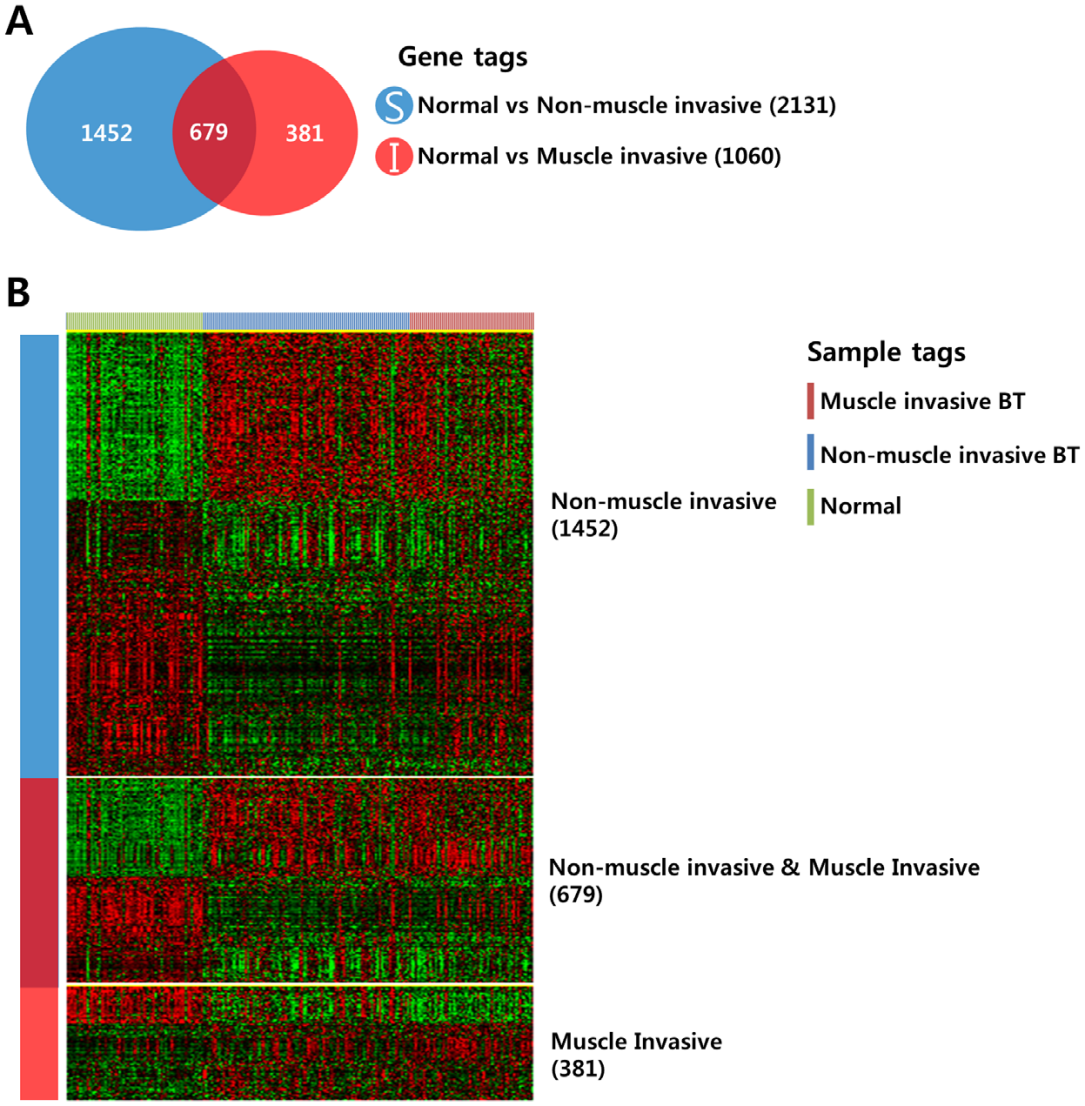
Comparative analysis of differentially expressed genes among the patient groups. (A) Venn diagram of genes selected by a 2-sample t-test. The blue circle (gene list S) represents genes differentially expressed between normal mucosa and NMIBCs. The red circle (gene list I) represents genes differentially expressed between normal mucosa and MIBCs. A cut-off *P*-value of less than 0.001 was applied to select genes with an expression that was significantly different between the two groups. (B) Expression patterns of selected genes in the Venn diagram. The data are presented in matrix format in which rows represent individual genes and columns represent each tissue. The red and green colors reflect high and low expression levels, respectively.

We next attempted to identify gene sets that were differentially expressed among the 3 different groups. We applied Venn diagram comparison of 2 gene lists to compare the gene expression patterns of NMIBCs and MIBCs. First, we generated 2 different gene lists by applying a 2-sample t-test (Figure 2A, *P*<0.001). Gene list A represents the genes that were differentially expressed between the normal mucosa and NMIBC, and gene list B represents the genes that were differentially expressed between the normal mucosa and MIBC. When comparing the 2 gene lists, 3 different patterns were observed: S not I (1,452 genes), S and I (679 genes), and I not S (381 genes) (Figure 2B). Genes in the S not I category showed NMIBC-specific expression patterns, while genes in the I not S category displayed MIBC-specific gene expression patterns. Genes in the S and I category exhibited both NMIBC and MIBC expression patterns, meaning 679 genes in the S and I category were common to both NMIBC and MIBC development.

### Functional Classification of the Gene Expression Signature for MIBC Development

**Figure 3:**
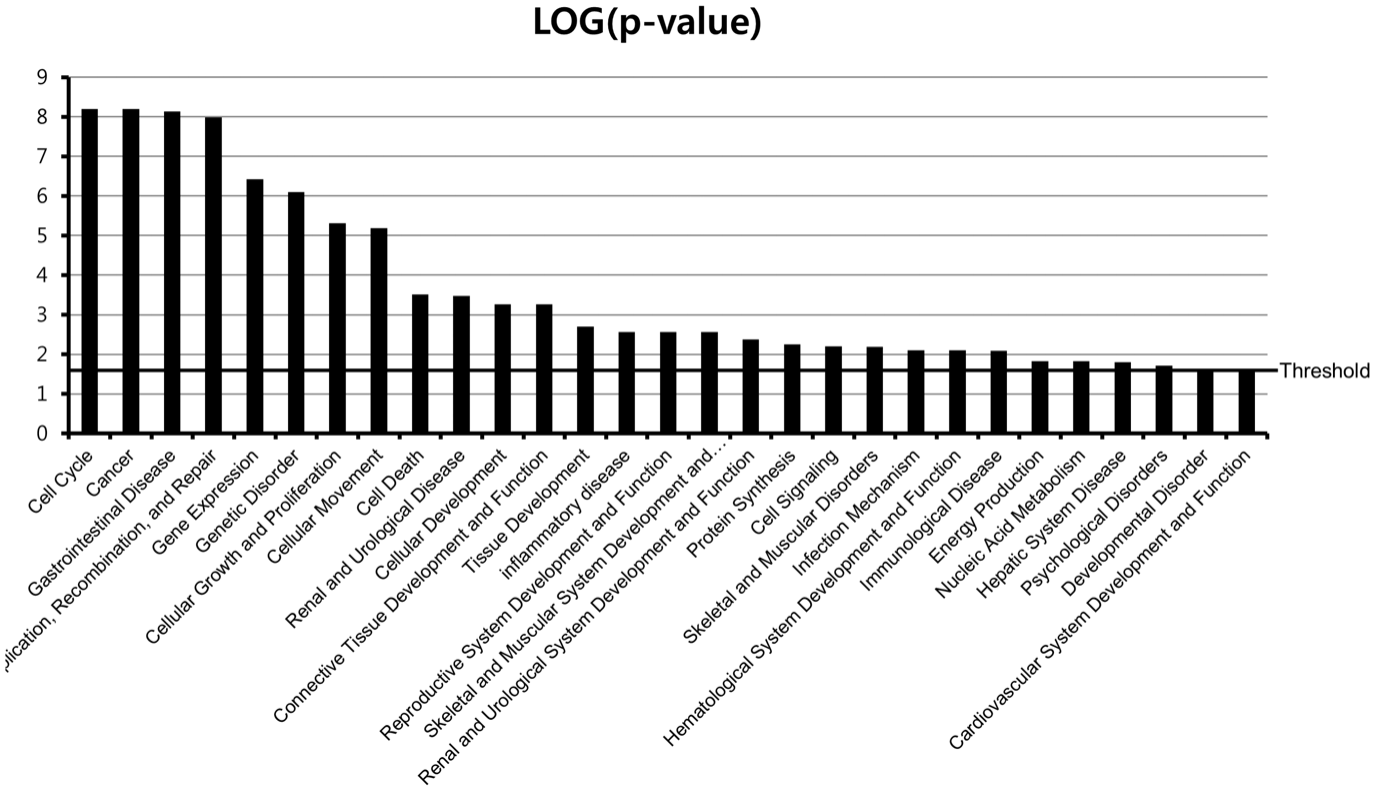
Functional classification of MIBC development-related genes. Classification enrichment was determined using Ingenuity Pathway Analysis software (version 8.8). The significance of each function was estimated using Fisher’s exact test method. The threshold of statistical significance was -log (*P* = 0.05).

To determine whether our gene expression signature was enriched in known biological functions, bioinformatic functional classification analyses of the genes that were differentially expressed between normal mucosa and MIBC were carried out. This analysis revealed a series of MIBC development associated with functional categories. Functional classifications of gene sets are illustrated in Figure 3. We found that genes involved in the cell cycle, cancer, cellular growth and proliferation, cell death, and DNA-replication and -repair were significantly enriched. We also found that genes involved in infection mechanisms, immunological disease, and inflammatory disease were also present in significant numbers. It is interesting that a significant number of genes involved in renal and urological disease, cellular development, tissue development, and developmental disorders were observed, which inspired confidence in our results. There has been much progress in bladder cancer research on genes that contribute to the cell cycle, cellular development, cell growth, or cell proliferation, which were highly significant functions in Figure 3. In order to identify the gene expression levels in bladder tumors, we further analyzed the microarray data sets. Individual tumor samples from bladder cancer patients revealed 664 genes that were differentially expressed in all of the tumor tissue samples, where differential genes were either up- or down-regulated (Table S1, S2, S3, and S4).

In the present study, we analyzed the expression profile of genes involved in the cellular proliferation, apoptosis, cell cycle control, angiogenesis, wound healing, DNA-replication and -repair, cytoskeleton, and cell adhesion between normal mucosa and MIBC (Table S1 and S2). In MIBC, the mRNA expression levels of the cellular proliferation-related genes VAX1, TTK, TPX2, TIMELESS, STIL, TBRG4, MCM7, KIF2C, HGS, DHCR7, CENPF, BRCA1, UHRF1, TRAIP, RECQL4, RACGAP1, NRAS, MKI67, KISS1R, KIF15, ING1, IMPDH1, FGF18, E2F1, DLG7, BUB1B, BUB1, BRCA2, and BLM were up-regulated compare with normal mucosa (Table S1). The next cluster was composed of apoptosis-related genes that were over-expressed (TOP2A, SCOTIN, MTP18, GSK3B, FANCG, BIRC5, AKT1S1, YARS, TRIB3, TRAF2, SCARB1, RAD21, FAF1, ESPL1, E2F2, BCL2L12, ATF5, and AATF) in MIBC (Table S1). The genes belonged to a family correlated with the cytoskeleton and were significantly over-expressed in MIBC: TUBG1, SPTBN2, SPAG5, SCYL1, RCC2, RAE1, PRC1, PPP4C, NUSAP1, MYOHD1, LMNB2, KIFC1, KIF4A, KIF20A, KIF14, KIAA1688, GTSE1, CCNB1, C9orf48, C18orf24, AZI1, AURKB, TUBA6, STMN1, SNIP, SLC9A3R1, SAC3D1, ODF2, NEK2, LMNB1, KNTC1, KIF22, ITGB4BP, FAM33A, CCNB2, and ANLN (Table S1). We also detected the up-regulated expression of cell adhesion-related molecules such as TSTA3, TROAP, ADAM15, TINAG, PVR, PPFIA1, CELSR3, and ADRM1 in MIBC (Table S1). Angiogenesis-related molecular genes including TOP2A, POLD1, Pfs2, ORC1L, MCM7, NPR1, and FGF18 are up-regulated in MIBC (Table S1). However, the up-regulated expression of angiogenic factor VEGF was unexpectedly observed in NMIBC (Table S1). DNA replication and repair-related genes were up-regulated in MIBC: TDP1, DNA2L, TYMS, TDG, POLQ, POLE2, POLD2, POLA2, ORC6L, MCM10, PRPF19, MGC32020, GTF2H4, EME1, RUVBL2, RAD51AP1, NUDT1, FANCB, and FANCA (Table S1). Finally, the present data showed the over-expressed levels of cell cycle regulated genes, which contribute to tumor progression and invasion: UBE2C, TREX1, RCC1, PSMD8, PA2G4, LIN9, GSG2, E2F3, CDT1, CDK5RAP1, CCNF, ZWINT, PKMYT1, CDC45L, CCNE2, CCNA2, SUV39H1, RAD54L, POLD1, HCAP-G, H2AFX, GPS1, FLJ22624, FANCD2, CHAF1A, CDCA3, ASPM, XRCC2, SPBC25, SMC4L1, SGOL1, SC65, PTTG1, POLE, PBK, MCM2, KNTC2, KIAA1794, EXO1, CIT, CHAF1B, CEP55, CDCA8, CDCA5, CDCA2, CDCA1, C20ort172, ANAPC11, CDC2, CDC20, CDC25C, CDC7, CDC27, and CDC25A (Table S1).

### IL-5, IL-20, and IL-28A Stimulate Migration and Invasion of Bladder Cancer Cells

**Figure 4:**
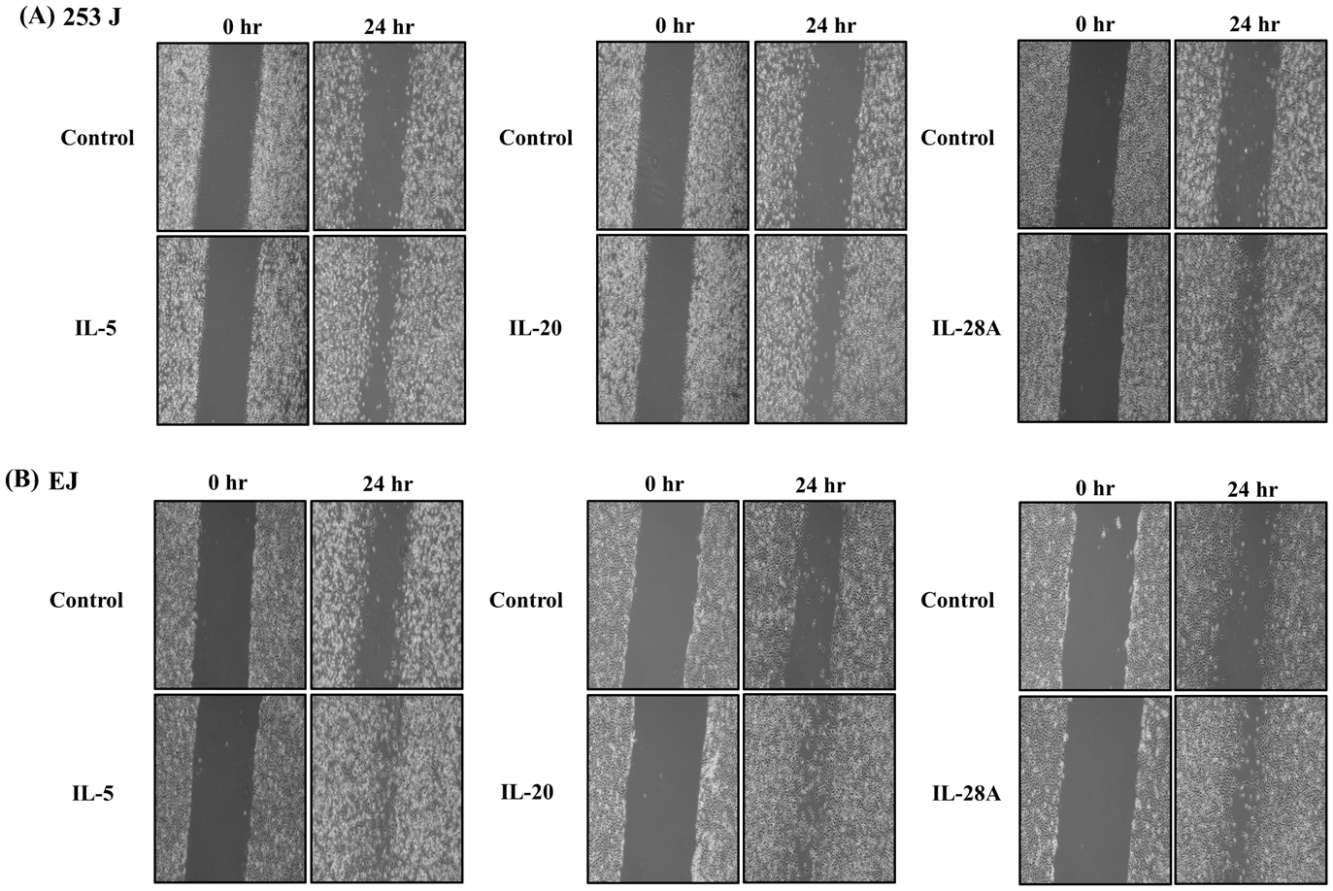
IL-5, IL-20, and IL-28A induced wound healing migration of bladder cancer 253J and EJ cells. (A, B) The confluent bladder cancer cells were incubated with serum free medium and treated with recombinant protein IL-5, IL-20, and IL-28A for the indicated times. The widths of injury lines made in cells were then examined at 0 and 24 h. Wound-healing migration is represented by the widths of injury lines.

**Figure 5:**
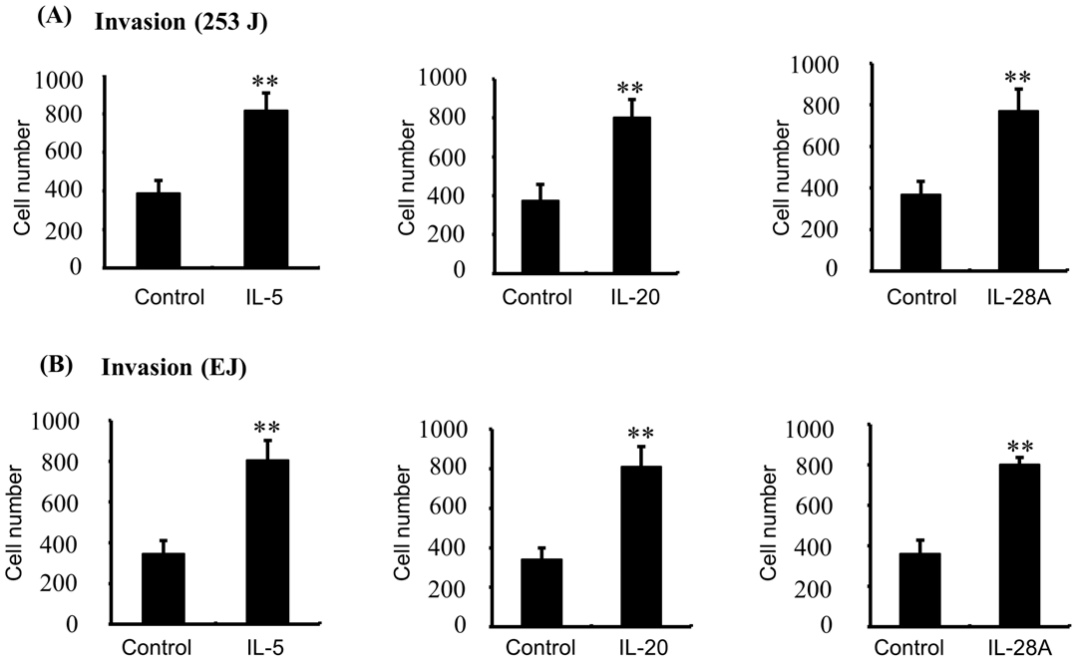
IL-5, IL-20, and IL-28A stimulated the invasion of bladder cancer cells. (A, B) The cells were placed in the upper chamber, and the indicated concentrations of IL-5, IL-20, and IL-28A were placed in the low well of the chemotaxis chamber. Invaded cell numbers were counted after the indicated times. The results are expressed as the number of invaded cells relative to an untreated control, as determined from 3 independent experiments. **P<0.01 compared with no treatment.

Bladder cancer is an immune- or inflammation-related disease, and immunotherapy such as intravesical instillation of Bacillus Calmette–Guérin **(**BCG) is the most important treatment option for NMIBC to prevent disease progression [5]–[7], [9]. Cumulative studies have indicated that alteration in cytokine levels might be a key event in determining the disease progression of bladder cancer [17]–[19]. For the next analysis, therefore, we selected immunological or inflammatory functions from our functional classification results (Figure 3). Because death in bladder cancer patients is strongly linked with the development of MIBC [4], we analyzed the gene expression patterns of representative immune- or inflammation-associated genes, comparing MIBC and control samples (Table 2 and 3). Ten (10) of these genes (IL-5, IL-26, IL-22RA1, IL-1RAPL1, IL-1F5, IL-17RB, IL-17RE, IL-20, IL-28A, and TRAF2) showed increased expression and 26 genes were down-regulated in MIBC samples, compared to control samples (Table 2 and 3). We divided the 10 up-regulated genes into 3 types: (1) cytokines (IL-26, IL-5, IL-20, and IL-1F5) (2) cytokine receptors (IL-22RA1, IL-17RB, IL-17RE, IL-1RAPL1, and IL-28AR1), and (3) cytokine receptor adaptor protein (TRAF-2). First, to examine the capacity to induce migration and invasion, we used cytokines IL-26, IL-5, IL-20 and IL-1F5. Second, we used cytokine IL-22, IL-17E, IL-17C, and IL-28A because these cytokines mediates cell responses through its interaction with their receptors (IL-22/IL-22RA1, IL-17E/IL-17RB, IL-17C/IL-17RE, IL-28A/IL28AR1) [20]–[23]. In the case of IL-1RAPL1, we used overexpression of the IL-1RAPL1 cDNA gene because the ligand of IL-1RAPL1 had not yet been identified [24]. Finally, we also used the TRAF2 cDNA gene we had cloned. Previous reports indicated that MIBC development was strongly associated with migration and invasion of cancer cells [4], [15]. To determine whether up-regulated genes in MIBC induce the migration and invasion of bladder cancer cells, we performed wound-healing assays and invasion assays in bladder cancer 253J and EJ cells using recombinant proteins or cDNA genes. Among the 10 molecules examined, IL-5, IL-20, and IL-28A significantly enhanced both wound-healing migration and invasion of 253J cells, compared with untreated cells (Figure 4A and 5A). Similar results were found in EJ cells (Figure 4B and 5B). In addition, cell proliferation was not observed in any of the 3 cases of cytokine-treated bladder cancer cells (Figure S1A and B). However, the other 5 cytokines and 2 genes had no effect on the migration and invasion of bladder cancer cells (Figure S2, S3, and S4).

**Table 2:** Differentially up-regulated genes in MIBC samples, compared to control samlples.

Gene name	Gene BankAccession No.	P-value
10 UP- regulated genes		
IL5	NM_000879.2	<0.001
IL26	NM_018402.1	<0.001
IL22RA1	NM_021258.2	<0.001
IL1RAPL1	NM_014271.2	<0.001
IL1F5	NM_173170.1	<0.001
IL17RB	NM_172234.1	<0.001
IL17RE	NM_153483.1	<0.001
IL20	NM_018724.3	<0.001
IL28RA1	NM_170743.2	<0.001
TRAF2	NM_021138.3	<0.001

P-value was less than 0.001.

**Table 3:** Differentially down-regulated genes in MIBC samples, compared to control samlples.

Gene name	Gene BankAccession No.	P-value
26 Down- regulated genes		
IL7R	NM_002185.2	<0.001
IL7	NM_000880.2	<0.001
IL6ST	NM_175767.1	<0.001
IL18RAP	NM_003853.2	<0.001
IL18R1	NM_003855.2	<0.001
IL16	NM_172217.1	<0.001
IL15	NM_172175.1	<0.001
IL11RA	NM_147162.1	<0.001
IL10RA	NM_001558.2	<0.001
TLR7	NM_016562.3	<0.001
HLA-DRB4	NM_021983.4	<0.001
HLA-DRA	NM_019111.3	<0.001
HLA-DQA1	XM_936120.1	<0.001
HLA-DPB1	NM_002121.4	<0.001
HLA-DPA1	NM_033554.2	<0.001
FCER1A	NM_002001.2	<0.001
CXCL12	NM_000609.4	<0.001
CLEC10A	NM_182906.2	<0.001
CFI	NM_000204.1	<0.001
CFD	NM_001928.2	<0.001
CD8A	NM_001768.4	<0.001
CCL14	NM_032962.2	<0.001
BCL2	NM_000657.2	<0.001
B2M	NM_004048.2	<0.001
TLR3	NM_003265.2	<0.001
CFH	NM_000186.2	<0.001

P-value was less than 0.001.

### IL-5, IL-20, and IL-28A Expression was Up-regulated in Patients with MIBC

**Figure 6:**
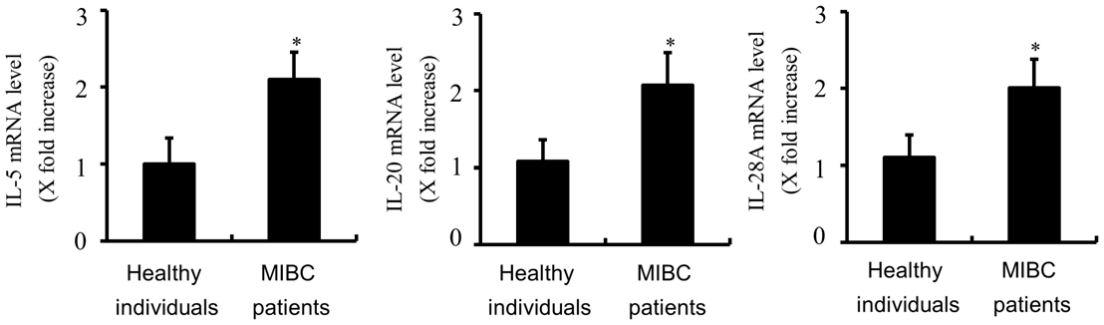
Increased expression of the mRNA levels of IL-5, IL-20, and IL-28A in MIBC patients. Quantitative real-time PCR was used to validate the gene expression of IL-5, IL-20, and IL-28A in MIBC patients and healthy individuals. Clinical samples were obtained from 62 MIBC patients and 68 healthy individuals. The mRNA was isolated and used to perform real-time PCR for IL-5, IL-20, and IL-28A. Results are represented as IL-5, IL-20, and IL-28A mRNA expression relative to GAPDH mRNA expression. *P<0.01 compared with healthy individuals.

To validate the level of IL-5, IL-20, and IL-28A, we next measured the mRNA levels of 62 MIBCs and 68 normal samples by real-time PCR. The results showed that the expression levels of IL-5, IL-20, and IL-28A mRNA were commonly higher in MIBC patients than in healthy individuals (Figure 6), which suggests that the expression of IL-5, IL-20, and IL-28A is strongly and significantly associated with invasive bladder cancer.

### IL-5, IL-20, IL-28A, and their Receptors Detected by Real-time PCR, Immunoblot and Confocal Immunofluorescence Microscopy in Bladder Cancer Cells

**Figure 7:**
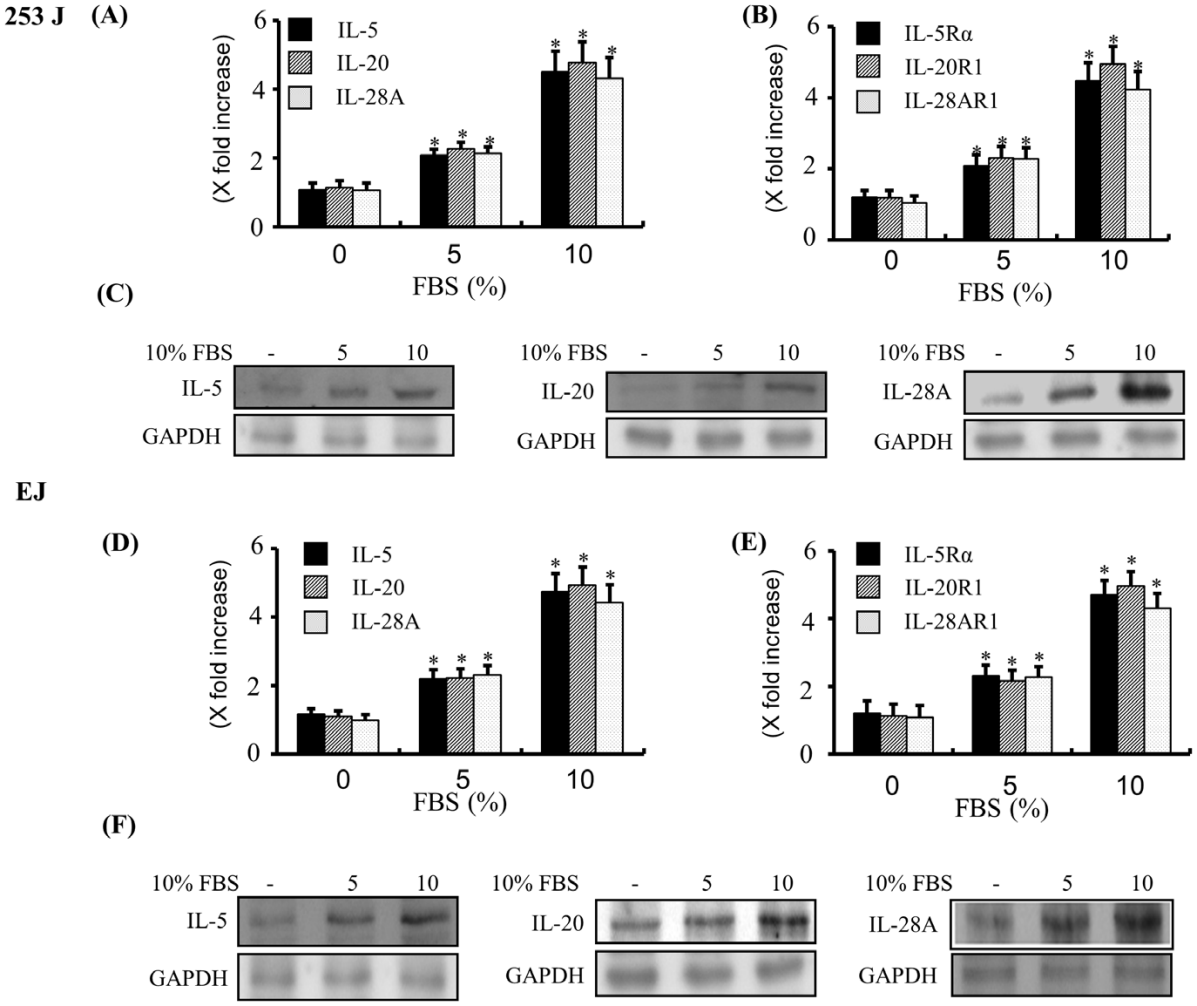
Expression of IL-5, IL-20, IL-28A, and their receptors in bladder cancer cells. (A, B, D, and E) Cells were incubated with the various concentrations of FBS for 24 h, and the mRNA expression levels of IL-5, IL-20, IL-28A (A, D) and their receptors (B, E) were quantified by real-time PCR. The results were expressed as IL-5, IL-20, IL-28A and their receptor’s mRNA expression relative to GAPDH mRNA expression. *P<0.01 compared with no treatment. (C, F) Cells were treated with various concentrations of FBS for 24 h, and protein levels of IL-5, IL-20, and IL-28A were analyzed by immunoblot. GAPDH expression was used as the loading control.

**Figure 8:**
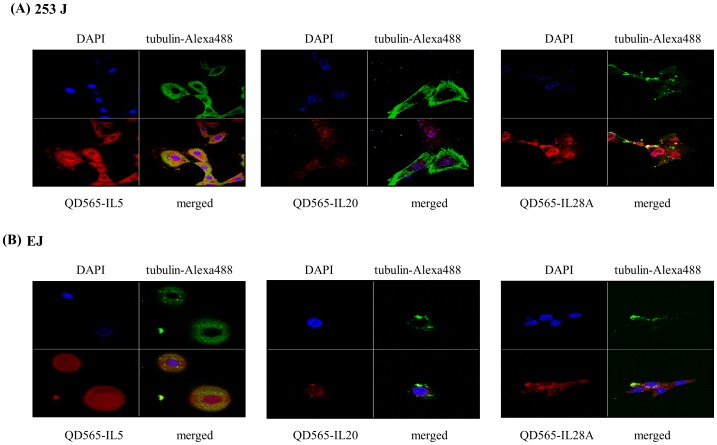
Confocal microscopy analysis of IL-5, IL-20, and IL-28A in bladder cancer 253J and EJ cells. (A, B) 253J and EJ cells, respectively, were stained with antibodies against QD565-conjugated IL-5, IL-20, and IL-28A (red). Both nuclei (anti-DAPI) and cytoplasm (anti-tubulin-Alexa488) are counterstained with DAPI (blue) and tubulin-Alexa488 (green).

In order to determine whether IL-5, IL-20, and IL-28A mRNA were expressed in both 253J and EJ cells, real-time PCR assay was carried out. As shown in Figure 7A and D, the expression of IL-5, IL-20, and IL-28A mRNA was endogenously detected in both cell lines. Treatment of both cell lines with 10% FBS for 24 h showed significant up-regulation of IL-5, IL-20, and IL-28A expression in mRNA levels (Figure 7A and D). In addition, real-time PCR analysis showed the expression of the receptors of 3 cytokines, IL-5Rα, IL-20R1, and IL-28AR1 in both 253J and EJ cells (Figure 7B and E). IL-5, IL-20, and IL-28A proteins were observed by immunoblot of protein extracts from both cell lines (Figure 7C and F). IL-5, IL-20, and IL-28A protein expression was increased by adding 10% FBS (Figure 7C and F). Using immunofluorescence confocal microscopy, we next examined the sub-cellular localization of IL-5, IL-20, and IL-28A protein in both cell lines. All 3 cytokines were dispersed in the cytoplasm and in the peri-nuclear areas (Figure 8A and B).

### IL-5, IL-20, and IL-28A Activates MMP-9 Expression via Activation of Transcription Factors NF-κB and AP-1 in Bladder Cancer Cells

**Figure 9:**
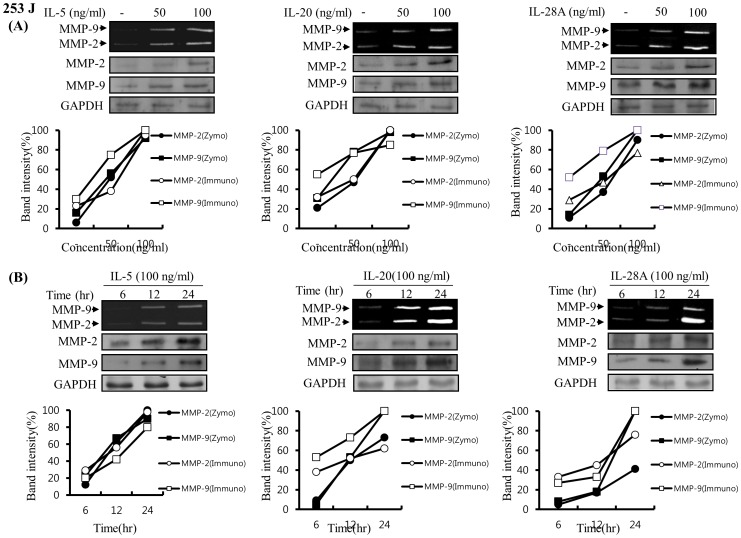
IL-5, IL-20, and IL-28A induced MMP-9 expression in human bladder cancer 253J cells. (A) Cells were grown to 70% confluence in DMEM supplemented with 10% FBS and the medium was changed to a serum-free medium. The cells were treated with different concentrations of IL-5, IL-20, and IL-28A for 24 h. (B) Time-dependent MMP-9 expression in bladder cancer cells induced by IL-5-, IL-20-, and IL-28A. Cells in serum-free medium were incubated with IL-5-, IL-20-, and IL-28A (100 ng/ml) for various times. Conditioned media from (A) and (B) were analyzed by zymographic MMP activity. Protein expression of MMP-2, MMP-9 and GAPDH was determined by immunoblot analysis.

**Figure 10:**
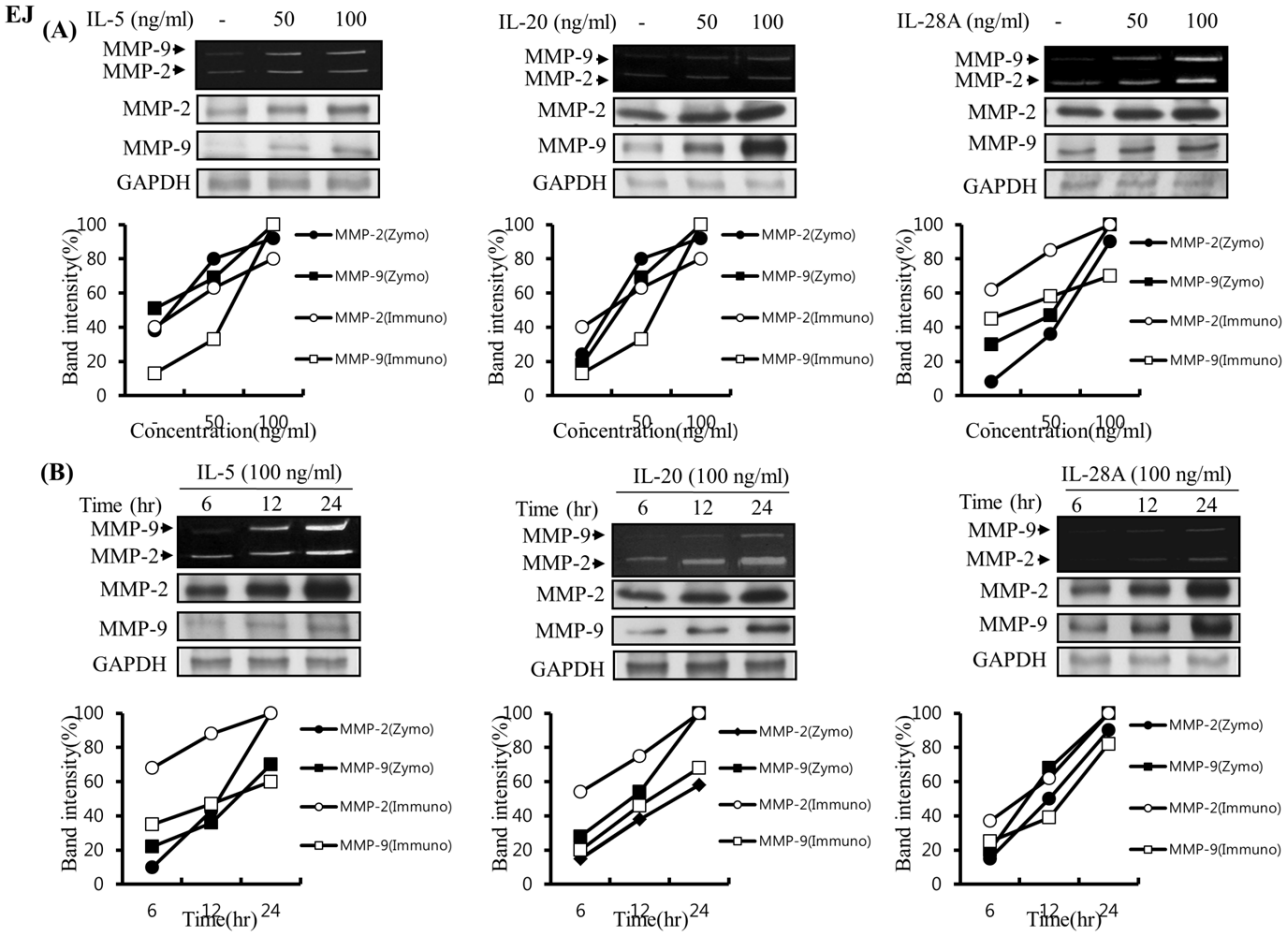
Induction of MMP-9 expression induced by IL-5, IL-20, and IL-28A in human bladder cancer EJ cells. (A) Confluent cells were cultured in DMEM supplemented with 10% FBS, and the medium was changed to a serum-free medium. The cells were stimulated with indicated concentrations of IL-5, IL-20, and IL-28A for 24 h. (B) Induction of time-dependent MMP-9 expression in IL-5-, IL-20-, and IL-28A-treated bladder cancer cells. Cells in serum-free medium were stimulated with IL-5-, IL-20-, and IL-28A (100 ng/ml) for indicated times. Zymographic MMP activity was analyzed using conditioned media from (A) and (B). Protein expression of MMP-2, MMP-9 and GAPDH was subject to immunoblot analysis.

**Figure 11:**
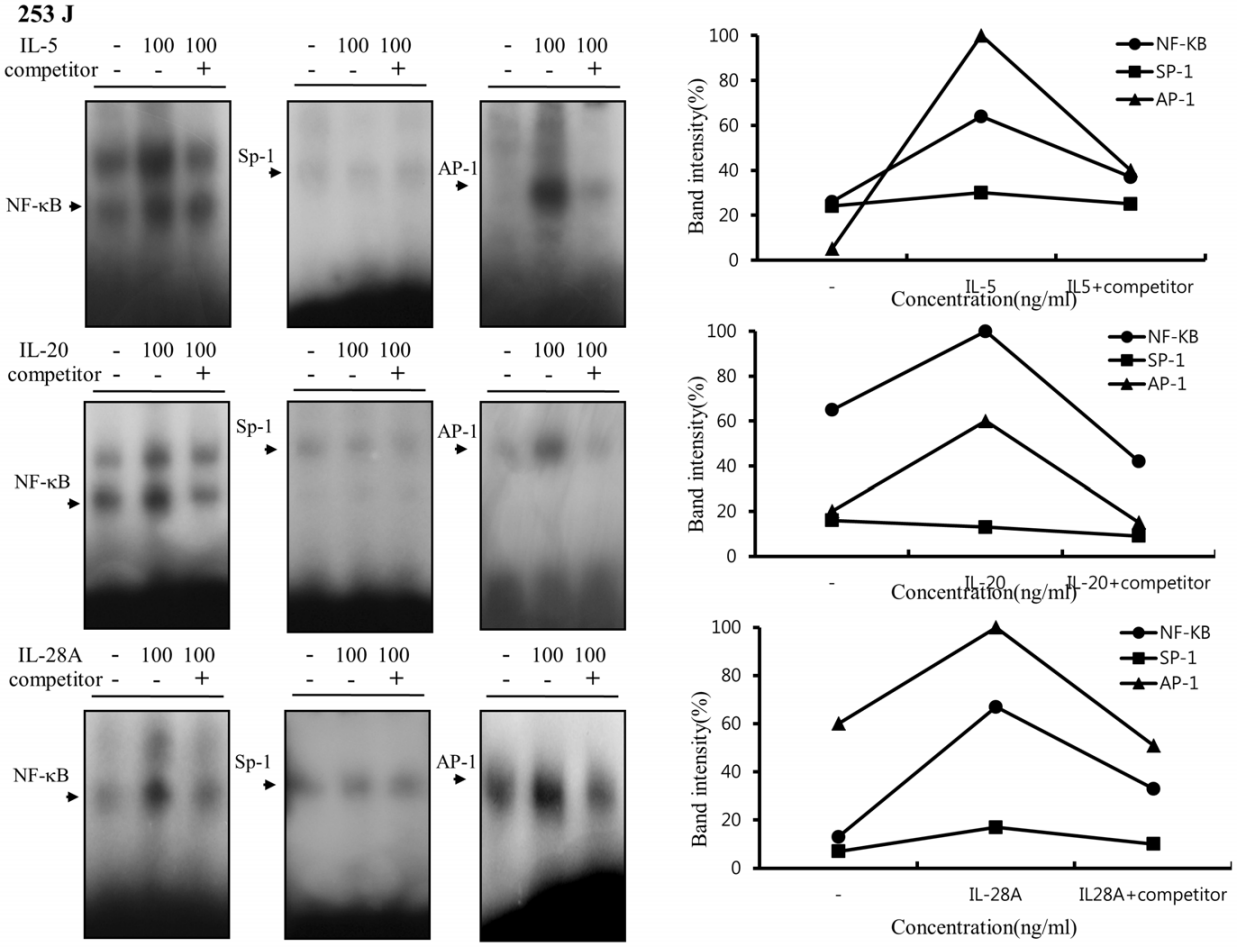
IL-5, IL-20, and IL-28A induced MMP-9 expression through increased DNA-binding activity of NF-κB and AP-1 in the MMP-9 promoter in bladder cancer 253J cells. Cells were cultured with serum-free medium containing the indicated concentrations of IL-5, IL-20, and IL-28A. After 24 h, nuclear extracts from the cells were analyzed by EMSA for activated NF-κB, AP-1, and Sp-1 using radiolabeled oligonucleotide probes.

**Figure 12:**
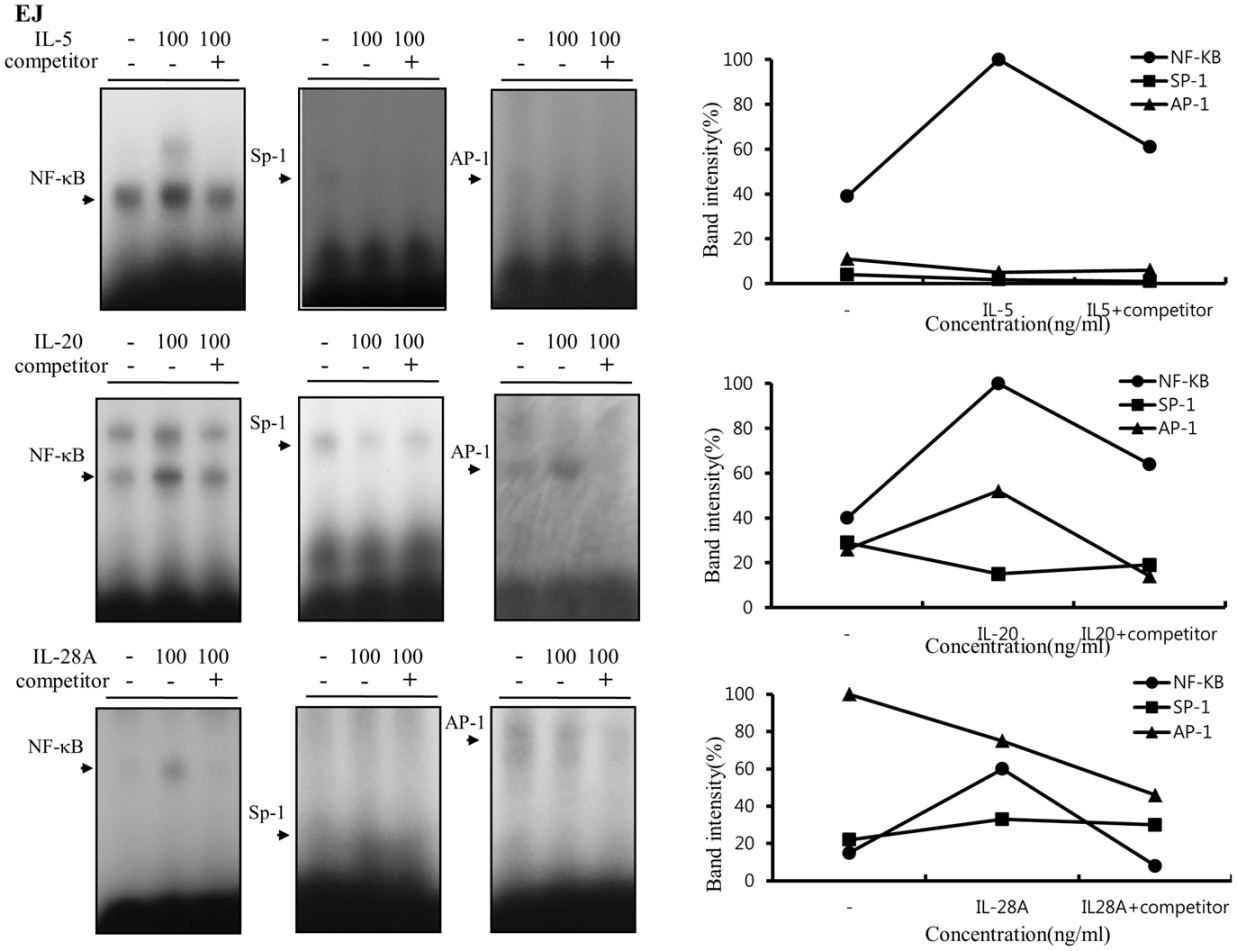
IL-5, IL-20, and IL-28A stimulated MMP-9 expression through increased DNA-binding activity of NF-κB and AP-1 motifs derived from the MMP-9 promoter in bladder cancer EJ cells. Cells were incubated with serum-free media for the indicated concentrations of IL-5, IL-20, and IL-28A for 24 h. Then, nuclear extracts from the cells were subjected to EMSA to test NF-κB, AP-1, and Sp-1 binding activity using radiolabeled oligonucleotide probes.

Previous reports demonstrated that MMP-9 expression was closely associated with bladder tumor invasion and migration [4], [15], [25]–[29]. Our data showed that IL-5, IL-20, and IL-28A stimulated the migration and invasion of bladder cancer cells (Figure 4 and 5). These results prompted us to examine whether IL-5, IL-20, and IL-28A induces MMP-9 expression. Treatment of both types of cancer cells with IL-5 resulted in significant up-regulation of MMP-9 expression in a concentration- and time-dependent manner, detected using gelatin zymography and immunoblot analysis (Figure 9A, 9B, 10A, and 10B). Similar results were observed after treatment with either IL-20 or IL-28A, respectively (Figure 9A, 9B, 10A, and 10B). In addition, the expression of MMP-2, another matrix metalloproteinase, was also stimulated in IL-5-, IL-20-, and IL-28A-treated cells, such as 253J and EJ cells (Figure 9A, 9B, 10A, and 10B). The 5′-regulatory region of the human MMP-9 promoter contains several consensus motifs for NF-κB, AP-1, and Sp-1 transcription factors [30]–[32]. We reasoned that MMP-9 expression by IL-5, IL-20, and IL-28A might be correlated with increased activity of NF-κB, AP-1, and Sp-1 in the nucleus. To this end, we performed an electrophoretic mobility shift assay (EMSA) using nuclear extracts of bladder cancer cells induced by IL-5, IL-20, and IL-28A. IL-5, IL-20, and IL-28A induced significant increase in NF-κB and AP-1 binding activities in 253J cell lines (Figure 11). No specific binding complexes into Sp-1 were observed in cells treated with any of the interleukins (Figure 11). However, in the case of EJ cells, both IL-5 and IL-28A stimulated NF-κB binding activity (Figure 12). Increased NF-κB and AP-1 binding activities were detected in IL-20-treated EJ cells (Figure 12).

### Induction of the MAPK and Jak-Stat Signaling Pathway in Bladder Cancer Cells Induced by IL-5, IL-20, and IL-28A

**Figure 13:**
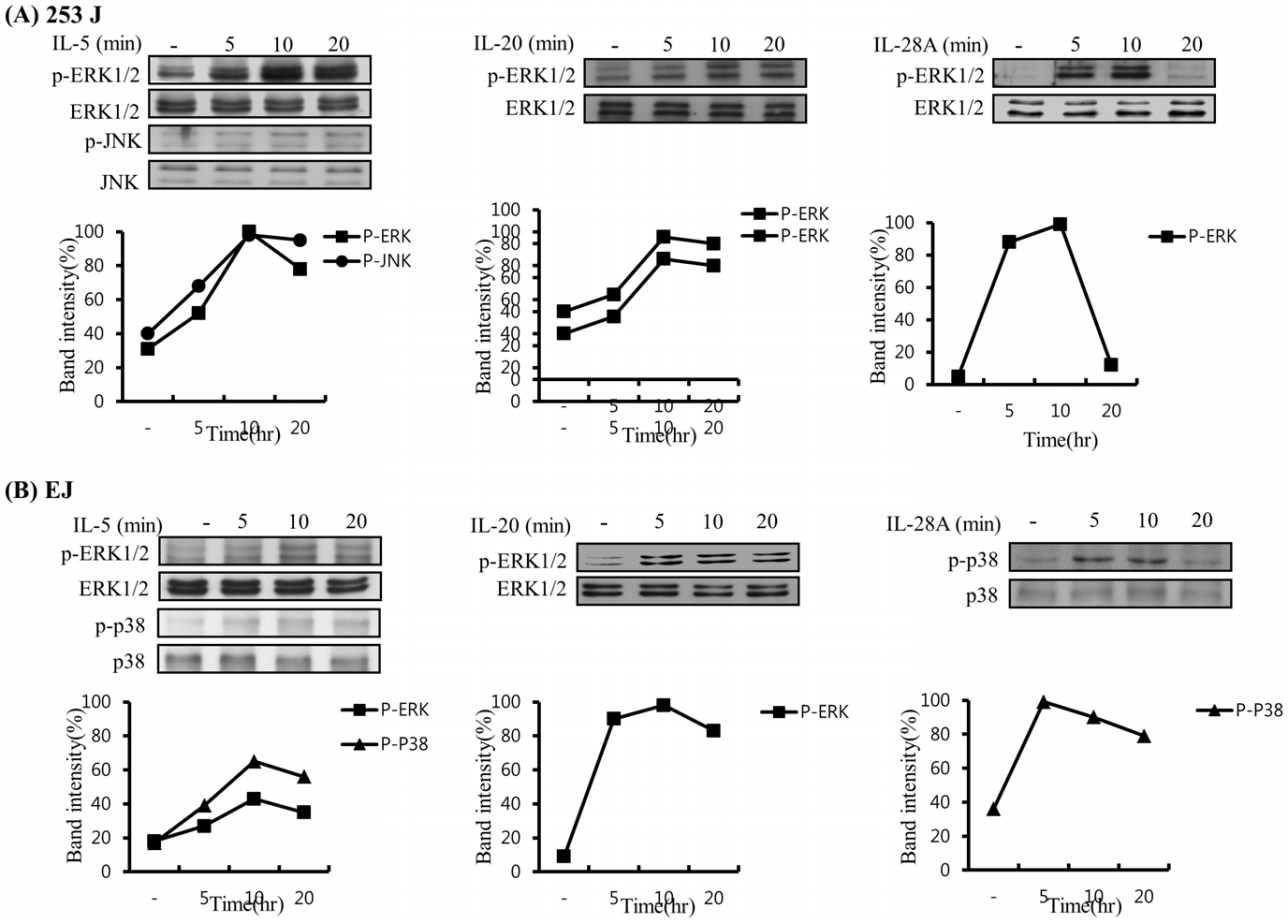
MAPK signaling pathway in IL-5-, IL-20-, and IL-28A-treated bladder cancer cells. (A, B) Cells were incubated in IL-5, IL-20, and IL-28A (100 ng/ml) for the indicated times, and were then harvested, lysed and subjected to immunoblot analysis for the activation levels of MAPK using specific antibodies.

**Figure 14:**
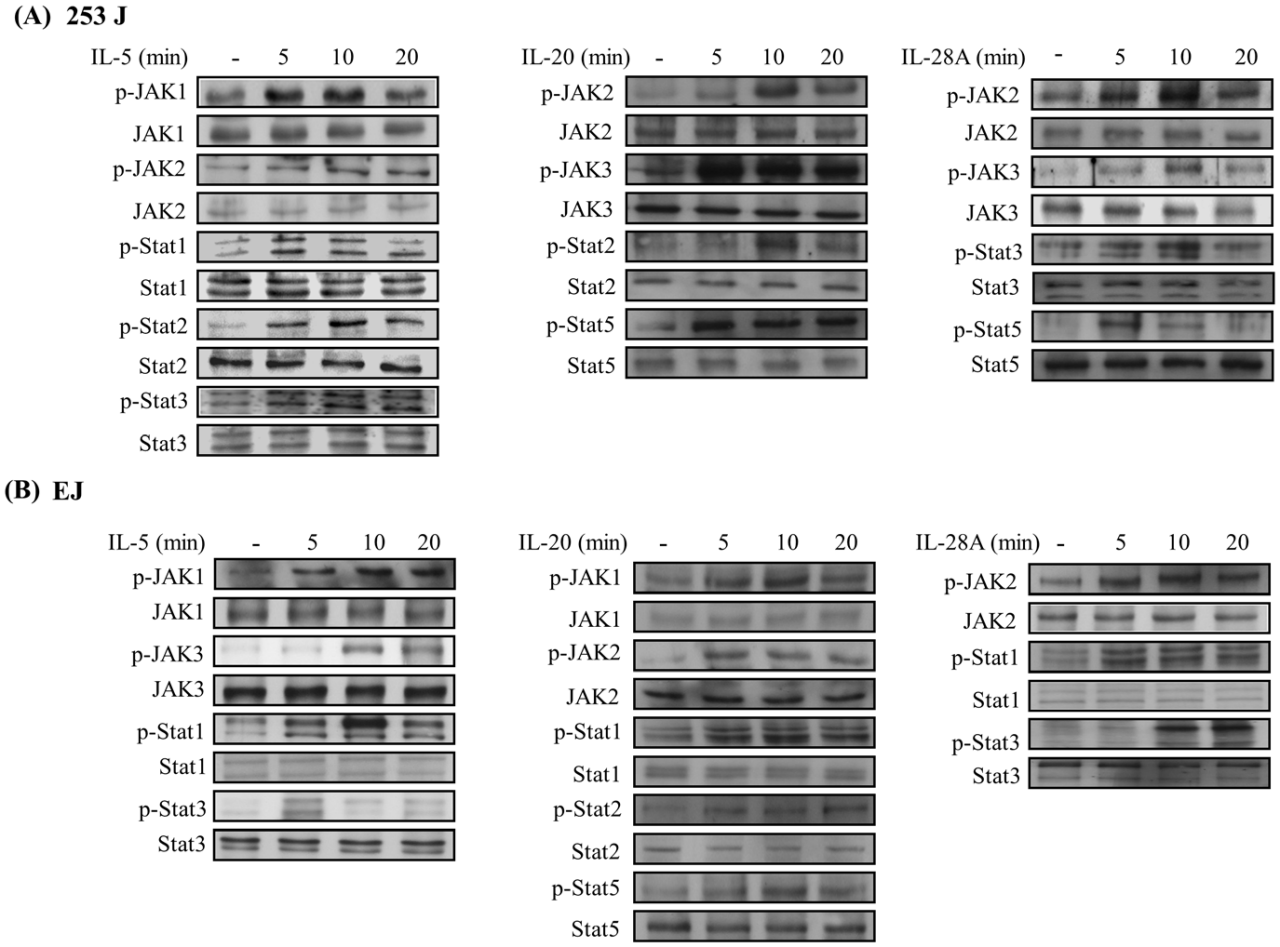
Effects of IL-5, IL-20, and IL-28A on the Jak-Stat signaling pathway in bladder cancer cells. (A, B) Cells were treated with IL-5, IL-20, and IL-28A (100 ng/ml) for the indicated times, and were then harvested. The activation levels of Jak-Stat were detected by immunoblot analysis using specific antibodies.

Because signaling for cytokines primarily activates the Jak/Stat and MAPK signal transduction pathways [8], we next investigated the signaling cascades induced by IL-5, IL-20, and IL-28A in bladder cancer cells. Time course experiments were performed in 253J and EJ cells. IL-5 treatment induced the activation of ERK1/2, JNK, JAK1, JAK2, Stat1, Stat2, and Stat3 in 253J cells (Figure 13A and 14A). Stimulation of EJ cells with IL-5 resulted in the activation of ERK1/2, p38MAPK, JAK1, JAK3, Stat1, and Stat3 (Figure 13B and 14B). In addition, IL-20 increased the activation of ERK1/2 in both 253J and EJ cells (Figure 13A and 13B). Activation of JAK2, JAK3, Stat2, and Stat5 was detected in IL-20-treated 253J cells (Figure 14A). Treatment with IL-20 stimulated the activation of JAK1, JAK2, Stat1, Stat2, and Stat5 in EJ cells (Figure 14B). In the case of IL-28A, the activation of ERK1/2 was observed in 253J cells (Figure 13A), p38MAPK activation was up-regulated in EJ cells (Figure 13B). Treatment of 253J cells with IL-28A induced the activation of JAK2, JAK3, Stat3, and Stat5 (Figure 14A). Moreover, the activation of JAK2, Stat1, and Stat3 was induced by IL-28A treatment in EJ cells (Figure 14B). However, AKT activation was not influenced in IL-5-, IL-20-, and IL-28A-treated bladder cancer cells (Figure S5A and B).

## Discussion

Many studies have used gene expression profiling of urinary bladder cancer using microarrays. Previous studies involving analysis of gene expression profiling have focused on cellular proliferation, cell cycle regulation, DNA replication and repair, apoptosis, signal transduction, transcription factors, angiogenesis, cell adhesion, wound healings, and the cytoskeleton. In the present study, the expression patterns of a number of tumor-related classical genes (eg, VEGF, PGF, FGF18, AKT, E2F1, ATF5) within our microarray dataset were detected as predicted. The hierarchical clustering analysis suggested that many genes may participate in regulatory networks involving the multiple biological systems that are required for bladder cancer development. However, little is known about the immunological or inflammatory-associated cytokines involved in the development of human urinary bladder cancer.

Based on the results from the present microarray dataset, we have determined the differences in immune responsive gene expression patterns between normal and MIBC. Ten (10) genes (Table 2) were up-regulated based on their gene expression patterns in MIBC, compared with normal mucosa samples, suggesting that these up-regulated genes are closely connected with the development of bladder cancer. At the first stage of the study, from these 10 genes we found 3 major cytokines, IL-5, IL-20, and IL-28A, which participate in migration, invasion, and MMP expression without affecting cell proliferation, indicating a coordinated program cluster to allow the progression of TCC as determined by the wound-healing migration, invasion assay, zymography, protein levels, and EMSA activity levels. In addition, we also identified that MAPK and Jak/Stat signaling are activated in bladder cancer cells following treatment with IL-5, IL-20, and IL-28A.

IL-5 was originally identified as a T-cell replacing factor (TRF), and was subsequently found to regulate the activation, proliferation, and survival of eosinophils [33]. IL-5 has also proven to be an important regulator for the differentiation of mouse B cells [34]. IL-5 receptor is a heterodimer composed of α- and β-subunits. The α-subunit is ligand-specific (IL-5Rα), whereas the β-subunit (βc) is common to IL-3 and IL-5 [33], [34]. Previous studies have shown that IL-5 activated Lyn [35], Jak2/Stat1 [36], MAPK [35], Syk [37], and PI3K [38] in eosinophils. The activation of Jak2, Btk tyrosine kinases, PI3K, Shc, Vav, and HS1was associated with IL-5-induced proliferation of B cells [39]. The IL-5 promoter contained essential transcription factors including Sp1, E12/E47, Oct-2, and c/EBPβ in B cells and eosinophils [40]. The use of rBCG vaccines for the treatment of bladder carcinomas did not produce TH-2 type cytokines including IL-5 levels [41]. In the present study, both IL-5 and IL-5Rα were detected by RT-PCR and immunoblot in bladder cancer cells. We have also identified the activation of ERK1/2, p38MAPK, JNK, JAK1, JAK2, JAK3, Stat1, Stat2, and Stat3 in bladder cancer cells. Our observation in this experiment is consistent with a recent report showing that the circulatory levels of IL-4, IL-5, and IL-10 were significantly higher in bladder cancer patient serum than in normal samples [19]. Thus, increases in IL-5 levels in this study might be responsible for augmented development of bladder tumor cells and their inability to be recognized by inflammatory.

IL-20, the pleiotropic inflammatory cytokine, is found in keratinocyte and identified as a member of the IL-10 family cytokines, which includes IL-10, IL-29, IL-20, IL-22, IL-24, and IL-26 [42], [43]. IL-20 stimulates signals through 2 alternative heterodimeric complexes, which consist of either IL-20R1 and IL-20R2 or IL-22R1 and IL-20R2 [42], [43]. Results from the present study showed expression of IL-20 and IL-20R1 in bladder cancer cells. With regard to signaling, IL-20 induced Stat3 activation in keratinocytes [42]. A previous report showed the activation of MAPK, such as ERK1/2, p38 MAPK, and JNK, in IL-20-treated HUVEC cells [44]. IL-20 treatment also induced the activation of Jak2/Stat3 and ERK1/2 pathway in GBM8901 glioblastoma cells [45]. Our results from bladder cancer cells indicate that IL-20 induced activation of ERK1/2 and Jak1, Jak2, Jak3, Stat1, Stat2, and Stat5. In addition, IL-20 is associated with multiple inflammatory diseases [45], [20], including psoriasis, rheumatoid arthritis, renal failure, brain injury, and atherosclerosis. In recent reports, IL-20 has regulated angiogenesis [44], [45]. In the present study, the up-regulation of IL-20 was not only shown in MIBC tissues but also produced by bladder cancer cells. The results of the present study demonstrate a key role for IL-20 in the development of MIBC.

Recently, new human IFN-λ proteins were discovered and named IFN-λ1 (IL-29), IFN-λ2 (IL-28A), and IFN-λ3 (IL-28B) [21], [22]. IL-28A forms signals through a heterodimer receptor complex consisting of IL-28AR1 and IL-10R2 [21], [22], [46]. IL-28A activates the JAK/STAT and MAPK pathways, which lead to the induction of antiviral, antiproliferative, antitumor, and immune responses. [21], [22], [46], [47]. From the perspective of biological function, many results have suggested anti-viral effects in several types of virus infection, including EMCV, HCV and CMV [21], [22], [46]–[48]. In addition to anti-viral effects, IL-28A also exhibits anti-proliferative and anti-tumor effects, *in vitro* and *in vivo*, in several types of tumor cells such as neuroendocrine tumor, colon cancer, murine melanoma, glioblastoma, and HaCaT cells [47], [49]–[52]. In the present study, we found high levels of IL-28A in MIBC, compared to normal samples. Bladder cancer cells expressed both IL-28A and IL-28AR1, as determined by RT-PCR and immunoblot. Binding of IL-28A to IL-28AR1 induced the activation of ERK1/2, p38MAPK, and Jak/Stat (Jak2, Jak3, Stat1, Stat3, and Stat5) signaling pathways in bladder cancer cells. Our results indicate that the expression of IL-28A in bladder cancer correlates with MIBC development.

The migration and invasion of cancer cells requires breakdown of the extracellular matrix (ECM) and basement membrane by proteases such as MMP-2 and MMP-9 [53], [54]. Recent advances in cancer cell biology have led to the association of MMP-2 and MMP-9 expression in bladder cancer progression [4], [15], [25]–[29]. The preclinical evidence supporting a role for MMP-9 in bladder cancer metastasis has been elucidated, in which increased MMP-9 expression correlates directly with high-grade and advanced-stage bladder tumors [4], [25]–[27]. Cumulative results have shown that NF-κB, AP-1, and Sp-1 can work as key transcription factors by inducing MMP-9 [30]–[32]. The present results indicate that transcription factors NF-κB and AP-1 are the main factors for MMP-9 induction in response to IL-5, IL-20, and IL-28A in bladder cancer cells. The up-regulation of MMP-9 activity by IL-5, IL-20, and IL-28A is considered to be an important mechanism to explain the association with increased metastatic ability. In addition, regarding the mechanism of the signaling pathway, it may be possible to link our data showing activation of MAPK and Jak/Stat signaling to increased MMP-9 expression induced by the interleukins described above, although the underlying exact and direct mechanism remains to be determined.

Cytokine secretion in cancer is supposedly related to a diverse range of cellular stresses, such as a carcinogen in response to injury, infection, or inflammation [55], [56]. Host responses to cellular stresses can influence several stages of tumor growth and metastasis. Production of inflammatory cytokines released by cancer cells may thus either accelerate cancer development and cancer cell growth or exhibit anti-tumor effects [12]–[16], [19]. The immune system can exert its effects as an extrinsic tumor suppressor, and cytokines mediated by BCG in bladder cancer play a key role in anti-tumor activity [5]–[7]. In addition, the importance and role of cytokines, including IL-2, IL-12, IFN-gamma, IL-6, and IL-15, in inhibiting the incidence and growth of bladder tumors has been established [12], [13], [19]. However, a critical role for bladder cancer cells, via the production of specific cytokines such as IL-6, IL-8, and IL-17, in promoting tumor growth has recently been demonstrated [14]–[16]. Unexpectedly, the results of the present study showed that 3 kinds of cytokines (IL-5, IL-20, and IL-28A) correlate with metastatic potential without altering cell proliferation, which results in bladder cancer cell migration and invasion through MMP-2 and MMP-9 expression. Thus, our results are the first to suggest that IL-5, IL-20, and IL-28A act as novel factors of migration and invasion in bladder carcinoma cells.

The results of the present study identified the 10 inflammatory-associated genes with at least a 2-fold increased expression in patients with MIBC, compared to normal tissue. Among the genes and proteins examined, we observed that IL-5, IL-20, IL-28A, and their receptors produced by bladder cancer cells induced migration, invasion, transcription factor-mediated MMP-9 expression, and activation of signaling pathways, such as the MAPK and Jak-Stat pathways. IL-5, IL-20, and IL-28A may thus be major molecules that characterize the migration and invasiveness of TCC, as well as the development of bladder cancer associated with disease progression. These cytokines could be investigated as new molecular targets for therapeutic treatment. In addition, further studies should examine the molecular mechanisms underlying the cytokines, which may be useful in determining which bladder tumors may progress.

## Materials and Methods

### Materials

Recombinant proteins IL-5, IL-20, and IL-28A and polyclonal antibodies to IL-5, IL-20, and IL-28A were purchased from R&D systems. (Minneapolis, MN). Polyclonal antibodies to ERK, phospho-ERK, p38MAPK, phospho-p38MAPK, JNK, and phospho-JNK were obtained from Cell Signaling (Danvers, MA). Polyclonal antibodies to Jak1, Jak2, Jak3, Stat1, Stat2, Stat3, Stat5, phospho-Jak1, phospho-Jak2, phospho-Jak3, phospho-Stat1, phospho-Stat2, phospho-Stat3, and phospho-Stat5 were purchased from Santa Cruz Biotechnology (Santa Cruz, CA).

### Ethics Statement

The Ethics Committee of Chungbuk National University approved the protocol used for this study. Written informed consent was obtained from all patients involved in this study. The Institutional Review Board of Chungbuk National University approved collection and analysis of all samples.

### Tissue Samples

All tumors were macro-dissected, typically within 15 minutes of surgical resection. Each bladder cancer specimen was confirmed by pathological analysis of a part of the tissue sample in fresh frozen sections from cystectomy and transurethral resection (TUR) specimens, and then frozen in liquid nitrogen and stored at −80°C until use.

### Human Bladder Cancer Microarrays

For initial gene expression profiling, we used a previously published data set of human bladder cancer microarrays, GSE13507, from the NCBI GEO public database [57]. The data set consisted of 165 primary bladder cancer samples (103 NMIBCs and 62 MIBCs), 58 samples of histologically normal-looking surrounding tissues, and 10 samples of normal bladder mucosae from patients with benign diseases. These gene expression data were constructed on Illumina HumanWG-6 BeadChips (version 2).

### RNA Extraction for Gene Expression Microarray Analysis

Total RNA was isolated from tissue using the TRIzol reagent (Life Technologies, NY), according to the manufacturer’s protocol. The quality and integrity of the RNA was confirmed by agarose gel electrophoresis and ethidium bromide staining, followed by visual examination under ultraviolet light.

### Microarray Gene Expression Profiling

Biotin-labeled cRNA for hybridization was prepared according to Illumina’s recommended sample labeling procedure. Briefly, 500 ng of total RNA was used for cDNA synthesis, followed by a coupled amplification/labeling step (in vitro transcription) to synthesize biotin-labeled cRNA using the Illumina® TotalPrep RNA Amplification kit (Ambion Inc., Austin, TX). The cRNA concentration was measured using RiboGreen (Quant-iT™ RiboGreen® RNA assay kit; Invitrogen-Molecular Probes, ON, Canada) and a Victor3 spectrophotometer (PerkinElmer, CT). cRNA quality was verified by 1% agarose gel electrophoresis.

Labeled, amplified material (1,500 ng per array) was hybridized to an Illumina Human-6 BeadChip (48K), version 2, according to the manufacturer’s instructions (Illumina, Inc., San Diego, CA). Array signals were developed using Amersham fluorolink streptavidin-Cy3 (GE Healthcare Bio-Sciences, Little Chalfont, UK), according to the instructions in the BeadChip manual. The arrays were scanned with an Illumina Bead Array Reader confocal scanner (BeadStation 500GXDW; Illumina, Inc., San Diego, CA), according to the manufacturer’s instructions.

### Statistical Analysis for Gene Expression Microarray Analysis

To compare the molecular characteristics between different patient groups, we performed a hierarchical clustering analysis. A hierarchical clustering algorithm, using the uncentered correlation coefficient as the measure of similarity and average linkage clustering, was applied as described in Eisen et al [58]. We identified genes that were differentially expressed between 2 groups using a 2-sample t-test [59]. Genes were considered to have statistically significant differences in expression if the *P*-value was less than 0.001. To survey the spectrum of biological functions within genes, which were differentially expressed between patient groups, we performed functional classification of the genes using Ingenuity™ Pathways Analysis software. The significance of each function was estimated using Fisher’s exact test method provided by the Ingenuity Pathway Analysis Tool (version 8.8).

### Cell Cultures

Human bladder carcinoma cell lines (253J and EJ) were obtained from the American Type Culture Collection. The cells were maintained in DMEM (4.5 g glucose/liter) supplemented with 10% fetal calf serum, l-glutamine, and antibiotics (Biological Industries, Beit Haemek, Israel) at 37°C in a 5% CO_2_ humidified incubator.

### Wound-healing Migration Assay

Cells were plated on 6-well dishes and grown to 90% confluence in 2 ml of growth medium. The cells were damaged using a 2-mm-wide tip and were then treated with IL-5, IL-20, or IL-28A. They were allowed to migrate, and photographs were taken through an inverted microscope (×40 magnification).

### Invasion Assay

Cells (2.5×10^4^) were resuspended with IL-5, IL-20, or IL-28A in 100 µL of medium and placed in the upper part of a transwell plate. The cells were then incubated for 24 hr. The cells had to pass through an 8 µm pore size polycarbonate membrane with a thin layer of ECM Matrix-like material. The ability of the cells to invade the ECM Matrix-like material was determined with a commercial cell invasion assay kit (Chemicon International, Billerica, MA).

### RNA Extraction and Construction of cDNA

RNA extraction for real-time PCR was performed as described below. cDNA was prepared from 1 µg of total RNA using a First-Strand cDNA Synthesis Kit (Amersham Biosciences Europe GmbH, Freiburg, Germany) according to the manufacturer’s instructions.

### Real-time PCR

Real-time PCR assays were performed essentially as previously described [55]. For amplification, IL-5 sense (5′-CATCCAGTGCTACTTGTGTT-3′); IL-5 anti-sense (5′- ACTTCAGGTCGAAGTCAATC-3′); IL-5R〈 sense (5′- GCAGAACGACCACTCACTA-3′); IL-5R〈 anti-sense (5′- GGTGCAGTGAAGGGAAACT-3′); IL-20 sense (5′-TTGCCTTCAGCCTTCTCTCT-3′); IL-20 anti-sense (5′-CCTTCCTCAGGTATCCTCTA-3′); IL-20R1 sense (5′- TACAATGGACTCCACCAGAG-3′); IL-20R1 anti-sense (5′- ACCGTCCACTTTCAGCCCAT-3′); IL-28A sense (5′-ACC GCT GAC ACT GAC CCA G-3′); IL-28A anti-sense (5′-CAG CCA GGG GAC TCC TTT-3′), IL-28AR1 sense (5′-CAG AAT GTG ACG CTG CTC TC-3′); and IL-28AR1 anti-sense (5′-ATC CAG GTA TTC GGA CTC CA-3′) primers were used. GAPDH was analyzed in parallel as an endogenous RNA reference gene, and data were normalized to the expression of GAPDH.

### Plasmid Construction and Cell Transfection

To obtain the full-length cDNA of TRAF2, first-strand cDNA synthesis was performed using a HelixCript™ 1^st^-Strand cDNA synthesis kit (NanoHelix Co., Ltd. Korea) according to the manufacturer’s instructions, with 5 µg of total RNA from EJ human bladder cancer cells. The full-length cDNA was obtained by PCR with first-strand cDNA as a template using a 5′ primer containing a *Hind*III site, 5′-ATCGAAGCTTCATGGCTGCAGCTAG CGT-3′ and a 3′ primer containing a *Xba*I site, 5′-TAGCTCTAGAGTTAGAGCCCTGTCAGGT-3′. PCR was performed as follows: 95°C for 60 s, 30 cycle of 95°C for 20 s, 56°C for 40 s, 72°C for 120 s, and 72°C for 5 min. The PCR product (1.5 kb) was cloned into pHelix-TA-vector (NanoHelix Co., Ltd. Korea) and determined by DNA sequencing. The inserted fragment (1.5kb) was cut out by digestion with *Hind*III and *Xba*I, and then inserted into the corresponding sites of pcDNA3 (Invitrogen, NY), which was designated pcDNA3-TRAF2. For the cloning of pcDNA3-IL-1RAPL1, we used specific primers from the human IL-1RAPL1 gene (2.679 kb): 5′- GGCCTTTAAGAGCTGGAAGAT-3′ (forward) and 5′-TCCCTTGCTTTTCTGTCACCA-3 (reverse).

Cells were transfected with pcDNA3-TRAF2, pcDNA3-IL-1RAPL1 or pcDNA3 (no insert) in 100 mm dishes using the Superfect reagent (Qiagen, Valencia, CA) according to the manufacturer’s protocol.

### Cell Proliferation

Cells were seeded into 12-well culture plates at 4×10^4^ cells/mL with DMEM containing 10% FBS. Cells were incubated at 37°C for 24 h. The medium was then replaced by serum-free medium. After 24h, the cells were stimulated with IL-5, IL-20 or IL-28A, and then trypsinized with trypsin-EDTA. Cells were counted using a coulter counter chamber (Coulter Corp, Florida).

### Immunoblot

Growth-arrested cells were treated with IL-5, IL-20, or IL-28A in the absence of 10% FBS for various durations at 37°C. The cells were then washed twice with cold PBS and freeze-thawed in 250 µL lysis buffer (containing, in mmol/L, HEPES [pH 7.5] 50, NaCl 150, EDTA 1, EGTA 2.5, DTT 1, ®-glycerophosphate 10, NaF 1, Na_3_VO_4_ 0.1, and phenylmethylsulfonyl fluoride 0.1 and 10% glycerol, 0.1% Tween-20, 10 ∝g/mL of leupeptin, and 2 µg/mL of aprotinin), and then scraped into 1.5-mL tubes. The lysates were placed on ice for 15 minutes and then centrifuged at 12, 000 rpm for 20 minutes at 4°C. The protein concentration of the supernatant was determined using a Bradford reagent method (Bio-Rad). Equal amounts of cellular proteins were resolved by electrophoresis on a 0.1% SDS–10% polyacrylamide gel (SDS-PAGE) under denaturing conditions. The proteins were transferred electrophoretically to nitrocellulose membranes (Hybond, Amersham Corp). After blocking in 10 mmol/L Tris-HCl (pH 8.0), 150 mmol/L NaCl, and 5% (wt/vol) nonfat dry milk, the membranes were treated with primary antibodies for 90 minutes, followed by incubation with peroxidase-conjugated secondary antibodies for 45 minutes. The immunocomplexes were detected using a chemiluminescence reagent kit (Amersham Corp). For the immunoblotting studies, the experiments were repeated at least 3 times [60].

### Preparation of IL-5-, IL-20-, and IL-28A-conjugated QD565

The carboxyl QD565 nanoparticles were covalently conjugated with the IL-5/20/28A (concentration: 10 ng/ml) by incubation for 1 h at room temperature with the addition of *N*-ethyl-N′-dimethylaminopropyl carbodiimide (EDC) to enhance the coupling efficiency between the amine and the carboxyl groups [61], [62]. The reaction ratio of the QD565 particles to the IL-5, IL-20, IL-28A, and EDC was 1∶2:1000. The QD565- IL-5/20/28A was centrifuged at 15,000 rpm for 15 min to remove the unconjugated free IL-5/20/28A and EDC; this was followed by several washing steps using Tris buffer solution (10 mM Tris–HCl, pH 7.4). After a brief sonication, the final conjugated products were mixed using a Tris-borate buffer solution (10 mM Tris–HCl, 10 mM sodium borate, pH 7.4; Sigma, St. Louis, MO).

### Confocal Microscopy of Il-5, IL-20 and IL-28A-QD565 Nanoparticles from the Cells

The cells were seeded into pre-coated gelatin 6-well plates and sterile cover slips were placed. The cells were then washed with twice phosphate-buffered saline (PBS). The antibody-conjugated QD565 nanoparticles described above were introduced with docking cells, and incubated for 4 h at 37°C. The cells were then fixed using a 3.7% formaldehyde solution (Sigma, St. Louis, MO) and were rinsed 3 times with PBS for 10 min. The cover slips from the 6-well plates were placed onto glass slides after mounting medium containing 4′,6-diamidino-2-phenylindole dihydrochloride (DAPI) solution was added (Vector Laboratories, Inc., CA). The fluorescence signal was detected using confocal laser scanning microscopy (Carl Zeiss LSM 510, Carl Zeiss, Jena, Germany).

### Zymography

Conditioned medium was electrophoresed in a polyacrylamide gel containing 1 mg/ml gelatin. The gel was then washed at room temperature for 2 h with 2.5% Triton X-100 and subsequently at 37°C overnight in a buffer containing 10 mM CaCl_2_, 150 mM NaCl, and 50 mM Tris–HCl, pH 7.5. The gel was stained with 0.2% Coomassie blue and photographed on a light box. Proteolysis was detected as a white zone in a dark blue field [60].

### Nuclear Extracts and Electrophoretic Mobility Shift Assay (EMSA)

Cultured cells were collected by centrifugation, washed and suspended in a buffer containing 10 mM Hepes (pH 7.9), 10 mM KCl, 0.1 mM EDTA, 0.1 mM EGTA, 1 mM DTT and 0.5 mM PMSF. After 15 min on ice, the cells were vortexed in the presence of 0.5% Nonidet NP-40. The nuclear pellet was then collected by centrifugation and extracted in a buffer containing 20 mM Hepes pH 7.9, 0.4 M NaCl, 1 mM EDTA, 1 mM EGTA, 1 mM DTT, and 1 mM PMSF for 15 min at 4°C.

The nuclear extract (10–20 µg) was preincubated at 4°C for 30 min with a 100-fold excess of an unlabeled oligonucleotide spanning the -79 MMP-9 *cis* element of interest. The sequences were as follows: AP-1, CTGACCCCTGAGTCAGCACTT; NF-κB, CAGTGGAATTCCCCAGCC; Sp-1, GCCCATTCCTTCCGCCCCCAGATGAAGCAG. The reaction mixture was then incubated at 4°C for 20 min in a buffer (25 mM HEPES buffer pH 7.9, 0.5 mM EDTA, 0.5 mM DTT, 0.05 M NaCl and 2.5% glycerol) with 2 µg of poly dI/dC and 5 fmol (2×10^4^ cpm) of a Klenow end-labeled (^32^P-ATP) 30-mer oligonucleotide, which spanned the DNA binding site in the MMP-9 promoter. The reaction mixture was electrophoresed at 4°C in a 6% polyacrylamide gel using a TBE (89 mM Tris, 89 mM boric acid and 1 mM EDTA) running buffer. The gel was rinsed with water, dried and exposed to X-ray film overnight [60].

### Statistical Analysis

For invasion and real-time PCR, when appropriate, data were expressed as the mean ± SE. Data were analyzed by factorial ANOVA and Fisher’s least significant difference test where appropriate. Statistical significance was set at *P*<0.05.

## Supporting Information

Figure S1 **Effect of IL-5, IL-20, and IL-28A in the proliferation of bladder cancer 253J and EJ cells.** (A, B) Confluent cells were cultured in serum-free medium for 24 h. The cells were then treated with IL-5, IL-20, and IL-28A for the indicated times. The cells were trypsinized with trypsin-EDTA, and counted by a coulter counter chamber.
(TIF)

Figure S2 **Effect of the other 7 up-regulated genes in wound healing migration of bladder cancer 253J and EJ cells.** The confluent bladder cancer cells were incubated with serum free medium and treated with recombinant protein IL-26, IL-1F5, IL-22, IL-17E, and IL-17C for the indicated times. The widths of injury lines made in cells were then examined at 0 and 24 h. Wound-healing migration is represented by the widths of injury lines. For the pcDNA3-TRAF2 and pcDNA3-IL-1RAPL1, cells were transfected with the pcDNA3 and pcDNA3-TRAF2 or pcDNA3-IL-1RAPL1 plasmids (2 µg), and the medium was changed to a serum-free medium. After 24 h, representative images of wound healing were taken on the time of the scratch and after 24 h of the wound scratch. The level of cell migration into the wound scratch was represented by the widths of injury lines.
(TIF)

Figure S3 **Effect of the other 7 up-regulated genes in wound healing migration of bladder cancer 253J and EJ cells.** The confluent bladder cancer cells were incubated with serum free medium and treated with recombinant protein IL-26, IL-1F5, IL-22, IL-17E, and IL-17C for the indicated times. The widths of injury lines made in cells were then examined at 0 and 24 h. Wound-healing migration is represented by the widths of injury lines. For the pcDNA3-TRAF2 and pcDNA3-IL-1RAPL1, cells were transfected with the pcDNA3 and pcDNA3-TRAF2 or pcDNA3-IL-1RAPL1 plasmids (2 µg), and the medium was changed to a serum-free medium. After 24 h, representative images of wound healing were taken on the time of the scratch and after 24 h of the wound scratch. The level of cell migration into the wound scratch was represented by the widths of injury lines.
(TIF)

Figure S4 **Effect of the other 7 up-regulated genes in the invasion of bladder cancer 253J and EJ cells.** The cells were placed in the upper chamber, and the indicated concentrations of IL-26, IL-1F5, IL-22, IL-17E, and IL-17C were placed in the low well of the chemotaxis chamber. Invaded cell numbers were counted after the indicated times. The results are expressed as the number of invaded cells relative to an untreated control, as determined from 3 independent experiments. ** *P*<0.01 compared with no treatment. For the pcDNA3-TRAF2 and pcDNA3-IL-1RAPL1, cells were transfected with the pcDNA3 and pcDNA3-TRAF2 or pcDNA3-IL-1RAPL1 plasmids (2 µg) and the cells were maintained in a serum-free medium for 24. After 24 h matrigel invasion under serum free medium conditions, invaded cell numbers were counted. The data are presented as the percentage of invasion relative to control vector.
(TIF)

Figure S5 **MAPK, AKT, and JAK-Stat signaling pathway in bladder cancer cells induced by IL-5, IL-20, and IL-28A.** (A, B) Cells were cultured with IL-5, IL-20, and IL-28A (100 ng/ml) for the indicated times. The total cell lysates were prepared and analyzed by immunoblot for the activation levels of signaling molecules using specific antibodies.
(TIF)

Table S1 **Up-regulated genes in bladder tumor samples, compared to normal tissue samples.**
(DOCX)

Table S2 **Down-regulated genes in bladder tumor samples, compared to normal tissue samples.**
(DOCX)

Table S3 **Up-regulated genes in muscle invasive bladder cancer (MIBC) samples, compared to non-muscle invasive bladder cancer (NMIBC) samples.**
(DOCX)

Table S4 **Down-regulated genes in muscle invasive bladder cancer (MIBC) samples, compared to non-muscle invasive bladder cancer (NMIBC) samples.**
(DOCX)
