# Supplementary figures and images for: Identification of Pro-Inflammatory Cytokines Associated with Muscle Invasive Bladder Cancer; The Roles of IL-5, IL-20, and IL-28A
Source: PLoS One. 2012 Sep 4;7(9):e40267. doi: 10.1371/journal.pone.0040267 (PMC3433484; doi:10.1371/journal.pone.0040267)

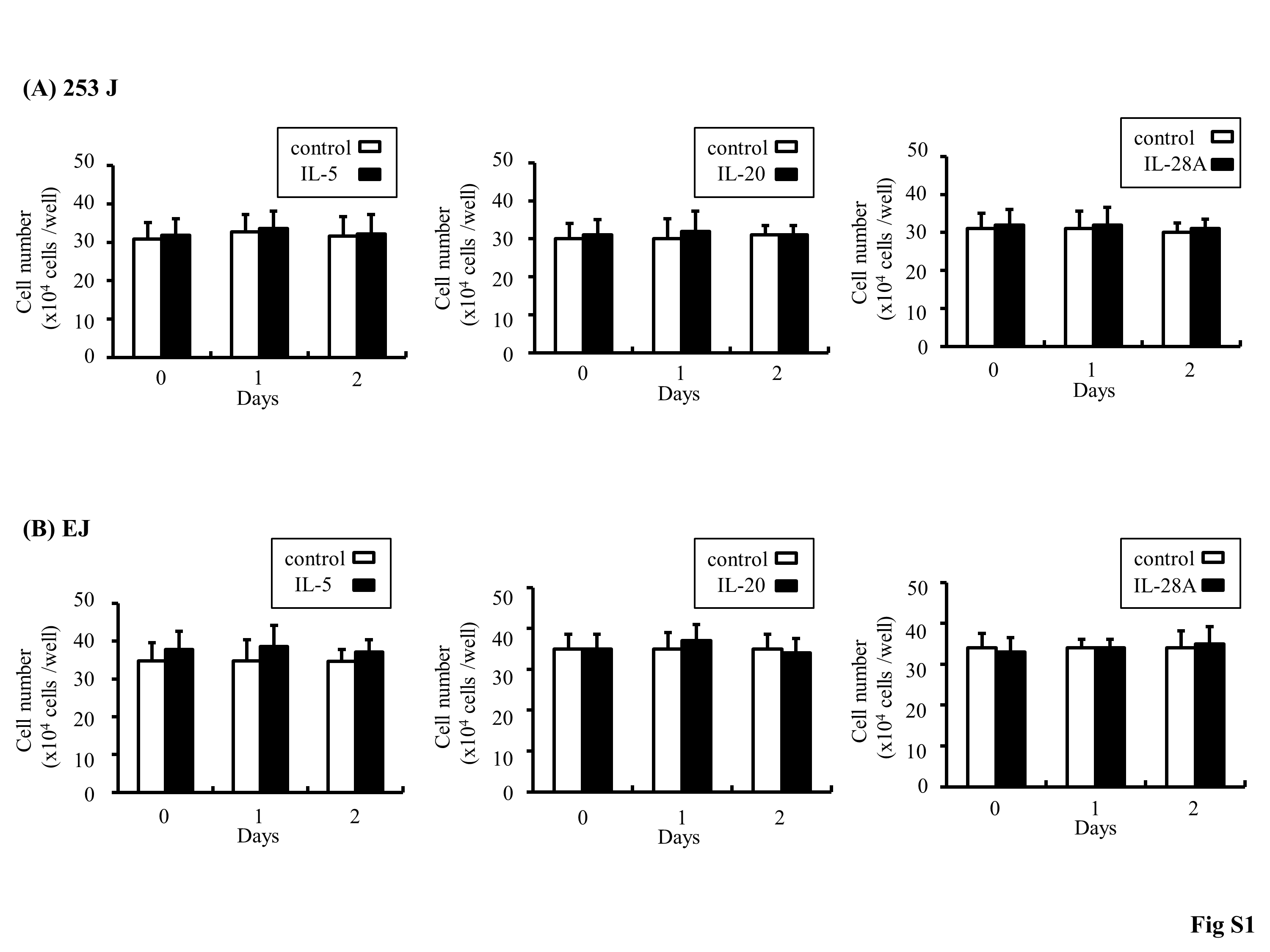

Supplement: Figure S1 — Effect of IL-5, IL-20, and IL-28A in the proliferation of bladder cancer 253J and EJ cells. (A, B) Confluent cells were cultured in serum-free medium for 24 h. The cells were then treated with IL-5, IL-20, and IL-28A for the indicated times. The cells were trypsinized with trypsin-EDTA, and counted by a coulter counter chamber. (TIF) [file pone.0040267.s001.tif]

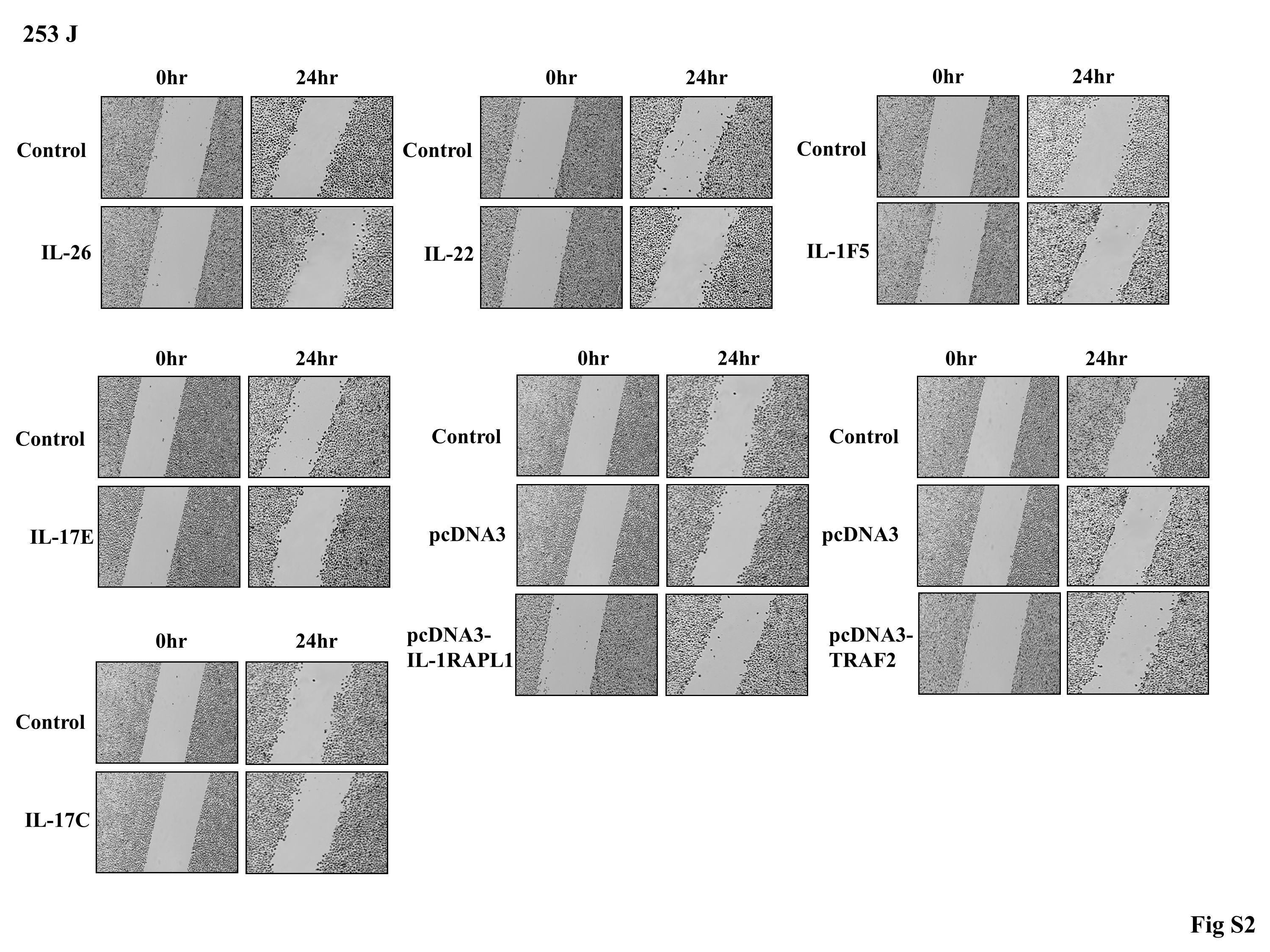

Supplement: Figure S2 — Effect of the other 7 up-regulated genes in wound healing migration of bladder cancer 253J and EJ cells. The confluent bladder cancer cells were incubated with serum free medium and treated with recombinant protein IL-26, IL-1F5, IL-22, IL-17E, and IL-17C for the indicated times. The widths of injury lines made in cells were then examined at 0 and 24 h. Wound-healing migration is represented by the widths of injury lines. For the pcDNA3-TRAF2 and pcDNA3-IL-1RAPL1, cells were transfected with the pcDNA3 and pcDNA3-TRAF2 or pcDNA3-IL-1RAPL1 plasmids (2 µg), and the medium was changed to a serum-free medium. After 24 h, representative images of wound healing were taken on the time of the scratch and after 24 h of the wound scratch. The level of cell migration into the wound scratch was represented by the widths of injury lines. (TIF) [file pone.0040267.s002.tif]

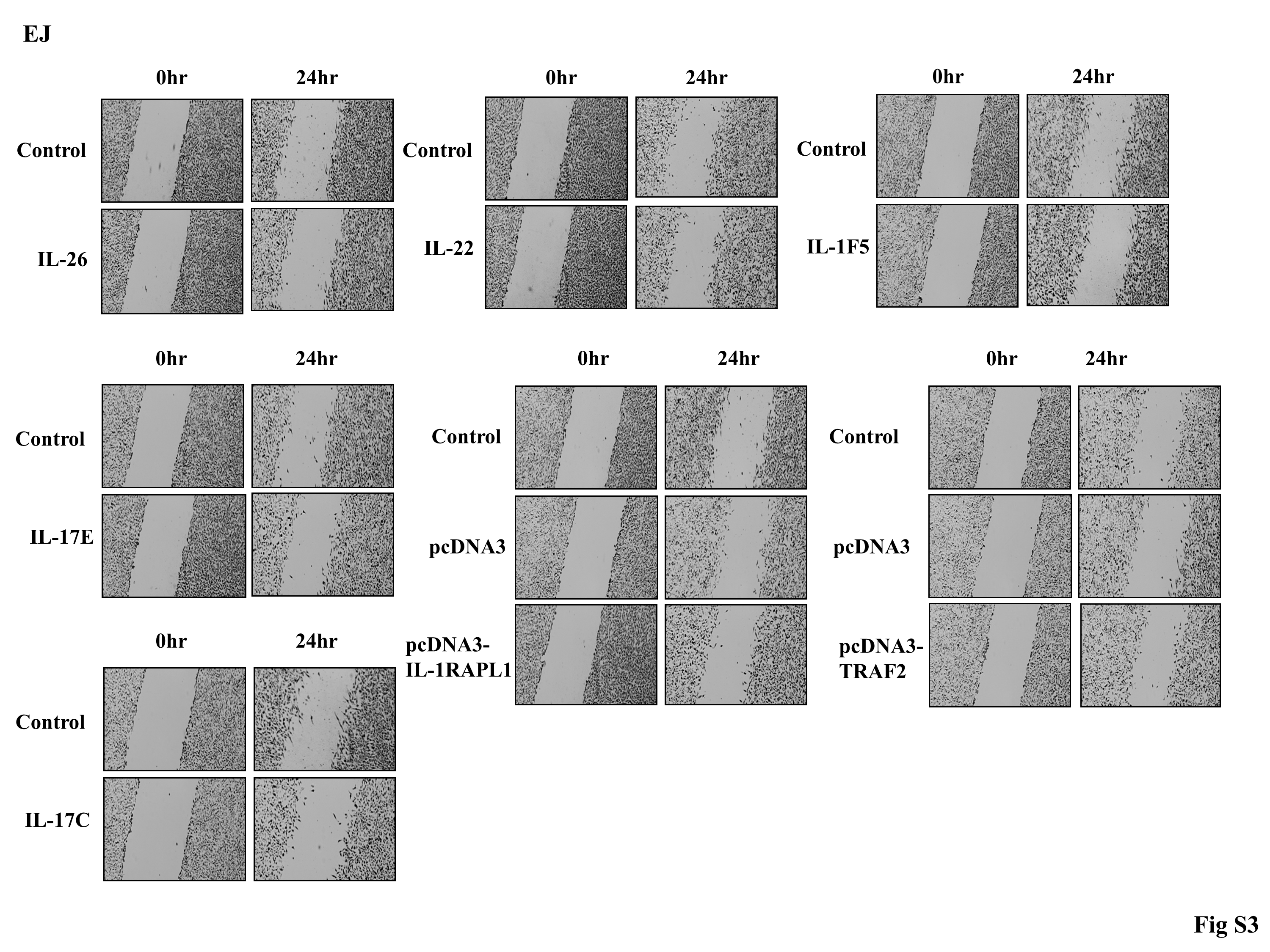

Supplement: Figure S3 — Effect of the other 7 up-regulated genes in wound healing migration of bladder cancer 253J and EJ cells. The confluent bladder cancer cells were incubated with serum free medium and treated with recombinant protein IL-26, IL-1F5, IL-22, IL-17E, and IL-17C for the indicated times. The widths of injury lines made in cells were then examined at 0 and 24 h. Wound-healing migration is represented by the widths of injury lines. For the pcDNA3-TRAF2 and pcDNA3-IL-1RAPL1, cells were transfected with the pcDNA3 and pcDNA3-TRAF2 or pcDNA3-IL-1RAPL1 plasmids (2 µg), and the medium was changed to a serum-free medium. After 24 h, representative images of wound healing were taken on the time of the scratch and after 24 h of the wound scratch. The level of cell migration into the wound scratch was represented by the widths of injury lines. (TIF) [file pone.0040267.s003.tif]

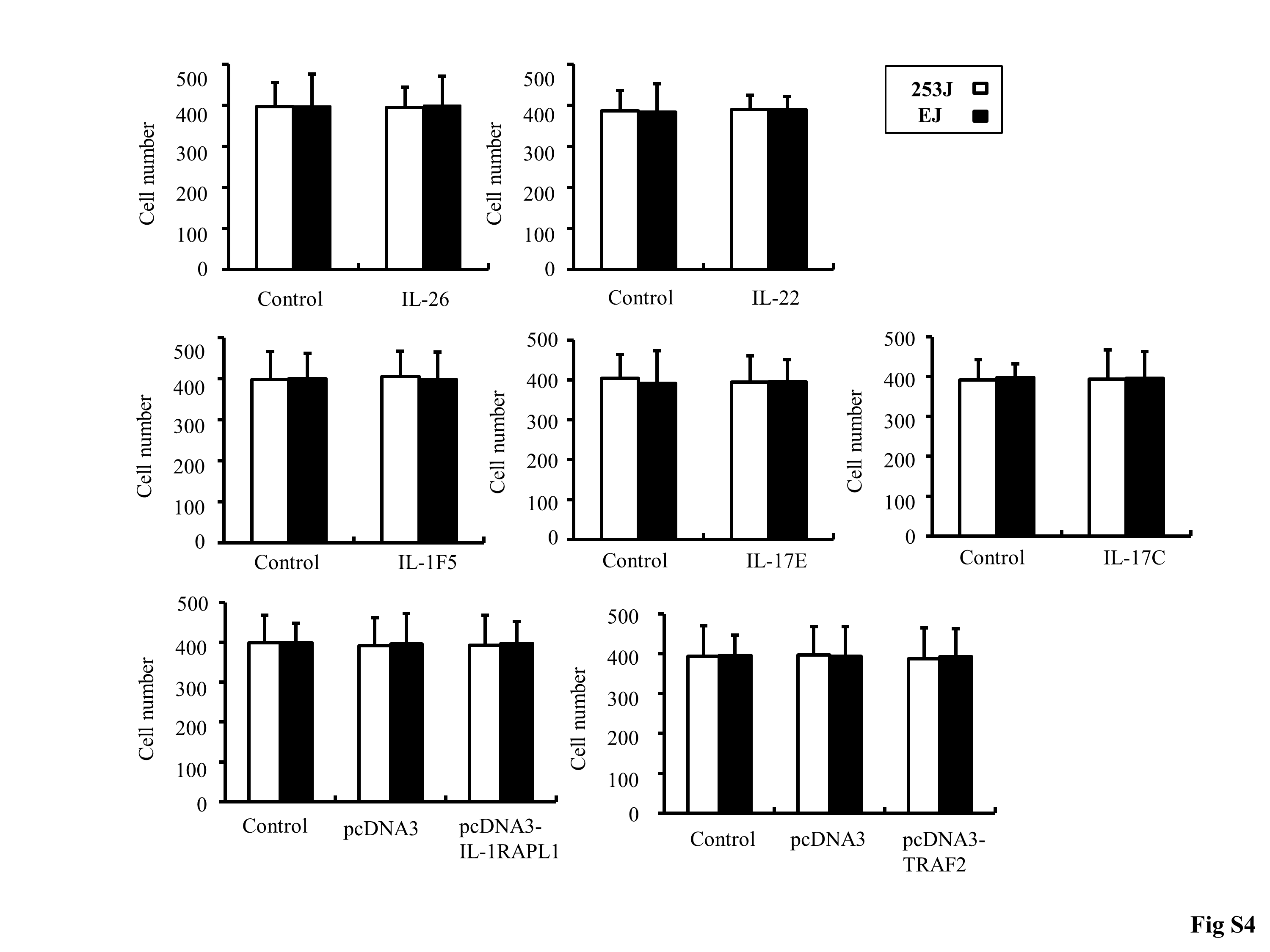

Supplement: Figure S4 — Effect of the other 7 up-regulated genes in the invasion of bladder cancer 253J and EJ cells. The cells were placed in the upper chamber, and the indicated concentrations of IL-26, IL-1F5, IL-22, IL-17E, and IL-17C were placed in the low well of the chemotaxis chamber. Invaded cell numbers were counted after the indicated times. The results are expressed as the number of invaded cells relative to an untreated control, as determined from 3 independent experiments. ** P<0.01 compared with no treatment. For the pcDNA3-TRAF2 and pcDNA3-IL-1RAPL1, cells were transfected with the pcDNA3 and pcDNA3-TRAF2 or pcDNA3-IL-1RAPL1 plasmids (2 µg) and the cells were maintained in a serum-free medium for 24. After 24 h matrigel invasion under serum free medium conditions, invaded cell numbers were counted. The data are presented as the percentage of invasion relative to control vector. (TIF) [file pone.0040267.s004.tif]

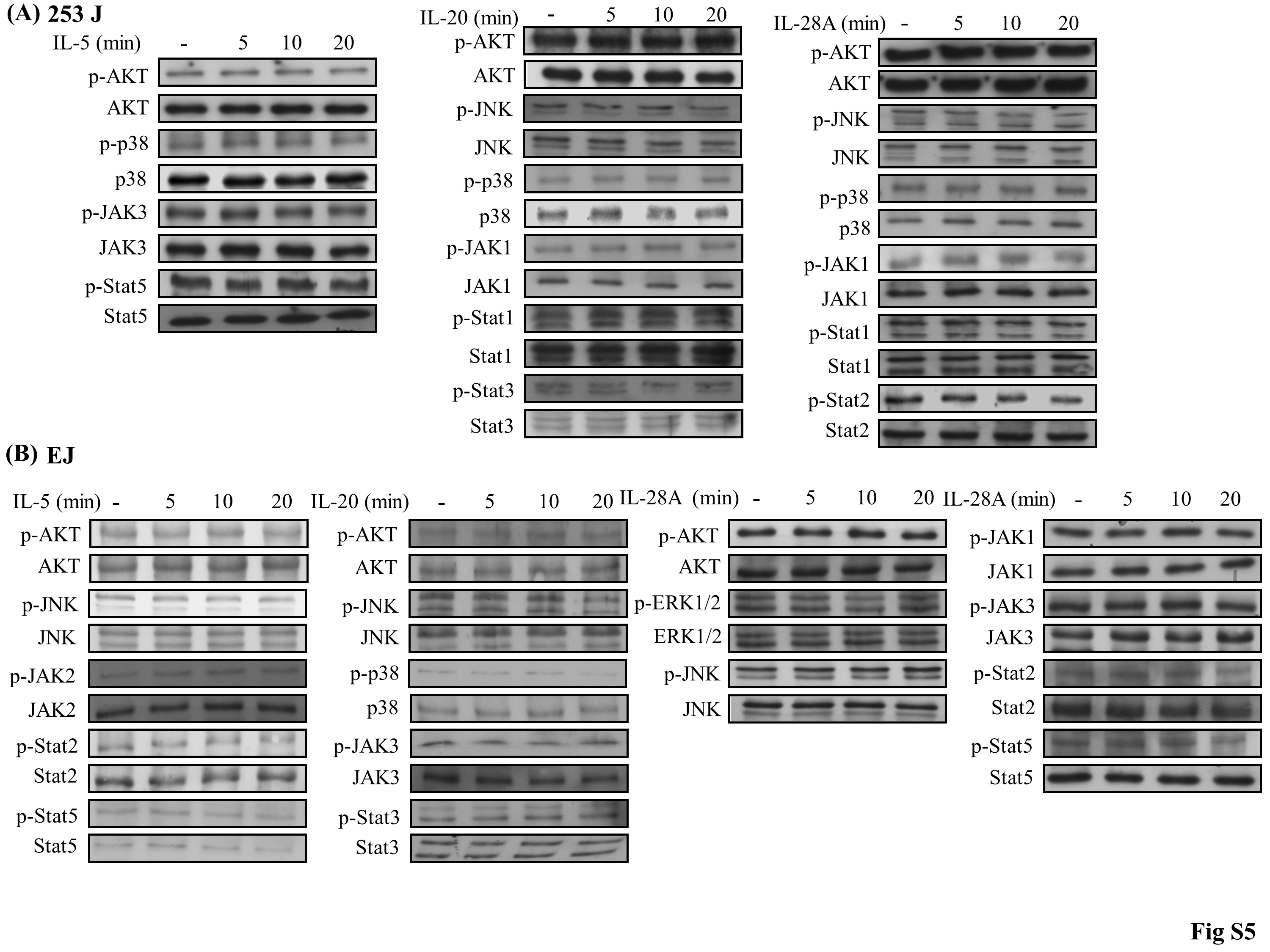

Supplement: Figure S5 — MAPK, AKT, and JAK-Stat signaling pathway in bladder cancer cells induced by IL-5, IL-20, and IL-28A. (A, B) Cells were cultured with IL-5, IL-20, and IL-28A (100 ng/ml) for the indicated times. The total cell lysates were prepared and analyzed by immunoblot for the activation levels of signaling molecules using specific antibodies. (TIF) [file pone.0040267.s005.tif]
