# Supplementary material for: Identification of Pro-Inflammatory Cytokines Associated with Muscle Invasive Bladder Cancer; The Roles of IL-5, IL-20, and IL-28A
Source: PLoS One. 2012 Sep 4;7(9):e40267. doi: 10.1371/journal.pone.0040267 (PMC3433484; doi:10.1371/journal.pone.0040267)
Supplement: Table S1 — Up-regulated genes in bladder tumor samples, compared to normal tissue samples. (DOCX) [file pone.0040267.s006.docx]

**Table S1. Up-regulated genes in bladder tumor samples, compared to normal tissue samples**

| response to wounding | | cell proliferation | | apoptosis | | immune response | | cytoskeleton | | cytoskeleton | | cell adhesion | |
| --- | --- | --- | --- | --- | --- | --- | --- | --- | --- | --- | --- | --- | --- |
|  |  |  |  |  |  |  |  |  |  |  |  |  |  |
| **Gene symbol** | **Class** | **Gene symbol** | **Class** | **Gene symbol** | **Class** | **Gene symbol** | **Class** | **Gene symbol** | **Class** | **Gene symbol** | **Class** | **Gene symbol** | **Class** |
| PROC | S not I | VHL | S not I | VHL | S not I | SPAG4 | S not I | TNS4 | S not I | SAC3D1 | I not S | TAOK2 | S not I |
| F12 | S not I | VEGF | S not I | VEGF | S not I | BCAP31 | S not I | PPP2R1A | S not I | RACGAP1 | I not S | F12 | S not I |
| AKT1 | S not I | SLC29A2 | S not I | TNS4 | S not I | TRAF2 | I not S | NARF | S not I | ODF2 | I not S | KPTN | S and I |
| MIF | S and I | SKP2 | S not I | TAOK2 | S not I | IL5 | I not S | MYH14 | S not I | NEK2 | I not S | PVRL4 | S not I |
| KPTN | S and I | PPARD | S not I | STEAP3 | S not I | IL26 | I not S | MICAL-L1 | S not I | LMNB1 | I not S | PPP2R1A | S not I |
|  |  | PGF | S not I | RAD9A | S not I | IL22RA1 | I not S | MARK2 | S not I | KNTC1 | I not S | PODXL2 | S not I |
|  |  | PES1 | S not I | PROC | S not I | IL1RAPL1 | I not S | KIAA1026 | S not I | KIF22 | I not S | HAPLN1 | S not I |
|  |  | PEMT | S not I | PRKCZ | S not I | IL1F5 | I not S | KATNB1 | S not I | KIF15 | I not S | CLSTN3 | S not I |
|  |  | IPF1 | S not I | PPP2R1A | S not I | IL17RB | I not S | HOOK1 | S not I | ITGB4BP | I not S | CLDN9 | S not I |
|  |  | ERBB2 | S not I | PPARD | S not I | IL17RE | I not S | CORO1B | S not I | FAM33A | I not S | CAMK2N1 | S not I |
|  |  | EIF2AK1 | S not I | PML | S not I | IL20 | I not S | BPY2IP1 | S not I | ESPL1 | I not S | ARVCF | S not I |
|  |  | VAX1 | S and I | PIK3R2 | S not I | IL28RA1 | I not S | ARVCF | S not I | DLG7 | I not S | TSTA3 | S and I |
|  |  | TTK | S and I | MAP3K10 | S not I |  |  | AKT1 | S not I | CDC27 | I not S | TROAP | S and I |
|  |  | TPX2 | S and I | FKBP8 | S not I |  |  | TUBG1 | S and I | CDC2 | I not S | ADAM15 | S and I |
|  |  | TIMELESS | S and I | FASTK | S not I |  |  | TTK | S and I | CCNB2 | I not S | TINAG | I not S |
|  |  | TBRG4 | S and I | EIF5A | S not I |  |  | TPX2 | S and I | BUB1B | I not S | SNIP | I not S |
|  |  | STIL | S and I | DNASE1 | S not I |  |  | TOP2A | S and I | BUB1 | I not S | SCARB1 | I not S |
|  |  | PA2G4 | S and I | CHEK2 | S not I |  |  | SPTBN2 | S and I | BRCA2 | I not S | PVR | I not S |
|  |  | MIF | S and I | BPY2IP1 | S not I |  |  | SPAG5 | S and I | ANLN | I not S | PPFIA1 | I not S |
|  |  | MCM7 | S and I | BCL7C | S not I |  |  | SCYL1 | S and I |  |  | FAF1 | I not S |
|  |  | KIF2C | S and I | BCAP31 | S not I |  |  | RCC2 | S and I |  |  | CELSR3 | I not S |
|  |  | HGS | S and I | AKT1 | S not I |  |  | RAE1 | S and I |  |  | ADRM1 | I not S |
|  |  | DHCR7 | S and I | TOP2A | S and I |  |  | PRC1 | S and I |  |  |  |  |
|  |  | CENPF | S and I | TBRG4 | S and I |  |  | PPP4C | S and I |  |  |  |  |
|  |  | CDC25C | S and I | SCOTIN | S and I |  |  | NUSAP1 | S and I |  |  |  |  |
|  |  | BRCA1 | S and I | MTP18 | S and I |  |  | MYOHD1 | S and I |  |  |  |  |
|  |  | UHRF1 | I not S | MIF | S and I |  |  | LMNB2 | S and I |  |  |  |  |
|  |  | TRAIP | I not S | GSK3B | S and I |  |  | KPTN | S and I |  |  |  |  |
|  |  | RECQL4 | I not S | FANCG | S and I |  |  | KIFC1 | S and I |  |  |  |  |
|  |  | RACGAP1 | I not S | BRCA1 | S and I |  |  | KIF4A | S and I |  |  |  |  |
|  |  | NRAS | I not S | BIRC5 | S and I |  |  | KIF2C | S and I |  |  |  |  |
|  |  | MKI67 | I not S | AKT1S1 | S and I |  |  | KIF20A | S and I |  |  |  |  |
|  |  | KISS1R | I not S | YARS | I not S |  |  | KIF14 | S and I |  |  |  |  |
|  |  | KIF15 | I not S | TRIB3 | I not S |  |  | KIAA1688 | S and I |  |  |  |  |
|  |  | ING1 | I not S | TRAIP | I not S |  |  | GTSE1 | S and I |  |  |  |  |
|  |  | IMPDH1 | I not S | TRAF2 | I not S |  |  | CENPF | S and I |  |  |  |  |
|  |  | FGF18 | I not S | SCARB1 | I not S |  |  | CDC20 | S and I |  |  |  |  |
|  |  | E2F1 | I not S | RAD21 | I not S |  |  | CCNB1 | S and I |  |  |  |  |
|  |  | DLG7 | I not S | NRAS | I not S |  |  | C9orf48 | S and I |  |  |  |  |
|  |  | CDC7 | I not S | FAF1 | I not S |  |  | C18orf24 | S and I |  |  |  |  |
|  |  | CDC27 | I not S | ESPL1 | I not S |  |  | BRCA1 | S and I |  |  |  |  |
|  |  | CDC25A | I not S | E2F2 | I not S |  |  | BIRC5 | S and I |  |  |  |  |
|  |  | BUB1B | I not S | E2F1 | I not S |  |  | AZI1 | S and I |  |  |  |  |
|  |  | BUB1 | I not S | CDC2 | I not S |  |  | AURKB | S and I |  |  |  |  |
|  |  | BRCA2 | I not S | BUB1B | I not S |  |  | TUBA6 | I not S |  |  |  |  |
|  |  | BLM | I not S | BCL2L12 | I not S |  |  | STMN1 | I not S |  |  |  |  |
|  |  |  |  | ATF5 | I not S |  |  | SNIP | I not S |  |  |  |  |
|  |  |  |  | AATF | I not S |  |  | SLC9A3R1 | I not S |  |  |  |  |

**Table S1. Up-regulated genes in bladder tumor samples, compared to normal tissue samples**

| regulation of progression | | cell cycle |  | cell cycle |  | angiogenesis |  | DNA replication |  | DNA repair |  | wound healing |  |
| --- | --- | --- | --- | --- | --- | --- | --- | --- | --- | --- | --- | --- | --- |
| through cell cycle |  |  |  |  |  |  |  |  |  |  |  |  |  |
| **Gene symbol** | **Class** | **Gene symbol** | **Class** | **Gene symbol** | **Class** | **Gene symbol** | **Class** | **Gene symbol** | **Class** | **Gene symbol** | **Class** | **Gene symbol** | **Class** |
| VHL | S not I | VHL | S not I | C18orf24 | S and I | VEGF | S not I | RAD9A | S not I | XAB2 | S not I | PROC | S not I |
| VEGF | S not I | VEGF | S not I | BRCA1 | S and I | PML | S not I | PPP2R1A | S not I | UNG | S not I |  |  |
| UHMK1 | S not I | UHMK1 | S not I | BIRC5 | S and I | PGF | S not I | PNKP | S not I | SMUG1 | S not I |  |  |
| TADA3L | S not I | TADA3L | S not I | AURKB | S and I | ERBB2 | S not I | PMS2L1 | S not I | RAD9A | S not I |  |  |
| SKP2 | S not I | STEAP3 | S not I | ASPM | S and I | TOP2A | S and I | PEO1 | S not I | PNKP | S not I |  |  |
| RPS6KB2 | S not I | ANGPT2 | S not I | ZWINT | I not S | POLD1 | S and I | TREX1 | S and I | PMS2L1 | S not I |  |  |
| RAD9A | S not I | RPS6KB2 | S not I | XRCC2 | I not S | Pfs2 | S and I | ATXN3 | S not I | PML | S not I |  |  |
| PPP2R1A | S not I | RAD9A | S not I | UHRF1 | I not S | ORC1L | S and I | ALKBH2 | S not I | CSNK1E | S not I |  |  |
| PML | S not I | PRM3 | S not I | STMN1 | I not S | MCM7 | S and I | TREX1 | S and I | RAD54L | S and I |  |  |
| PGF | S not I | PPP2R1A | S not I | SPBC25 | I not S | NPR1 | I not S | TOP2A | S and I | PRPF19 | S and I |  |  |
| MCRS1 | S not I | PML | S not I | SMC4L1 | I not S | FGF18 | I not S | TDP1 | S and I | POLD1 | S and I |  |  |
| SKP2 | S not I | PGF | S not I | SGOL1 | I not S |  |  | LIN9 | S and I | MGC32020 | S and I |  |  |
| SCC-112 | S not I | PES1 | S not I | SC65 | I not S |  |  | DNA2L | S and I | H2AFX | S and I |  |  |
| FZR1 | S not I | MCRS1 | S not I | SAC3D1 | I not S |  |  | CHAF1A | S and I | GTF2H4 | S and I |  |  |
| CHEK2 | S not I | KLHDC3 | S not I | RAD21 | I not S |  |  | CDT1 | S and I | FANCG | S and I |  |  |
| CCNT1 | S not I | KATNB1 | S not I | RACGAP1 | I not S |  |  | TYMS | I not S | FANCD2 | S and I |  |  |
| UBE2C | S and I | GADD45GIP1 | S not I | PTTG1 | I not S |  |  | TDG | I not S | EME1 | S and I |  |  |
| TTK | S and I | FZR1 | S not I | POLE | I not S |  |  | POLQ | I not S | CHAF1A | S and I |  |  |
| TREX1 | S and I | DDX12 | S not I | PKMYT1 | I not S |  |  | POLE2 | I not S | BRCA1 | S and I |  |  |
| TIMELESS | S and I | CUL7 | S not I | PBK | I not S |  |  | POLE | I not S | XRCC2 | I not S |  |  |
| TBRG4 | S and I | CHEK2 | S not I | NRAS | I not S |  |  | POLD2 | I not S | UHRF1 | I not S |  |  |
| RCC1 | S and I | CCNT1 | S not I | NEK2 | I not S |  |  | POLA2 | I not S | TYMS | I not S |  |  |
| PSMD8 | S and I | UBE2C | S and I | MKI67 | I not S |  |  | ORC6L | I not S | TDG | I not S |  |  |
| PA2G4 | S and I | TUBG1 | S and I | MCM2 | I not S |  |  | MCM2 | I not S | RUVBL2 | I not S |  |  |
| NUSAP1 | S and I | TTK | S and I | KNTC2 | I not S |  |  | MCM10 | I not S | RECQL4 | I not S |  |  |
| LIN9 | S and I | TREX1 | S and I | KNTC1 | I not S |  |  | EXO1 | I not S | RAD51AP1 | I not S |  |  |
| GTSE1 | S and I | TPX2 | S and I | KIF22 | I not S |  |  | CHAF1B | I not S | RAD21 | I not S |  |  |
| GSG2 | S and I | TIMELESS | S and I | KIF15 | I not S |  |  | CDC7 | I not S | PTTG1 | I not S |  |  |
| FANCG | S and I | TBRG4 | S and I | KIAA1794 | I not S |  |  | CDC45L | I not S | POLQ | I not S |  |  |
| E2F3 | S and I | SUV39H1 | S and I | ING1 | I not S |  |  | CCNE2 | I not S | POLE2 | I not S |  |  |
| CENPF | S and I | SPAG5 | S and I | FAM33A | I not S |  |  | BRCA2 | I not S | POLE | I not S |  |  |
| CDT1 | S and I | RCC2 | S and I | EXO1 | I not S |  |  | BLM | I not S | NUDT1 | I not S |  |  |
| CDK5RAP1 | S and I | RCC1 | S and I | ESPL1 | I not S |  |  |  |  | KIF22 | I not S |  |  |
| CDC25C | S and I | RAD54L | S and I | E2F6 | I not S |  |  |  |  | KIAA1794 | I not S |  |  |
| CDC20 | S and I | PSMD8 | S and I | E2F2 | I not S |  |  |  |  | FANCB | I not S |  |  |
| CCNF | S and I | PRC1 | S and I | E2F1 | I not S |  |  |  |  | FANCA | I not S |  |  |
| CCNB1 | S and I | POLD1 | S and I | DLG7 | I not S |  |  |  |  | EXO1 | I not S |  |  |
| BRCA1 | S and I | PA2G4 | S and I | CIT | I not S |  |  |  |  | CHAF1B | I not S |  |  |
| BIRC5 | S and I | NUSAP1 | S and I | CHAF1B | I not S |  |  |  |  | BRCA2 | I not S |  |  |
| ZWINT | I not S | MCM7 | S and I | CEP55 | I not S |  |  |  |  | BLM | I not S |  |  |
| PKMYT1 | I not S | LIN9 | S and I | CDCA8 | I not S |  |  |  |  |  |  |  |  |
| NRAS | I not S | KIFC1 | S and I | CDCA5 | I not S |  |  |  |  |  |  |  |  |
| NEK2 | I not S | KIF2C | S and I | CDCA2 | I not S |  |  |  |  |  |  |  |  |
| MKI67 | I not S | HCAP-G | S and I | CDCA1 | I not S |  |  |  |  |  |  |  |  |
| KNTC1 | I not S | H2AFX | S and I | CDC7 | I not S |  |  |  |  |  |  |  |  |
| ING1 | I not S | GTSE1 | S and I | CDC45L | I not S |  |  |  |  |  |  |  |  |
| ESPL1 | I not S | GSG2 | S and I | CDC27 | I not S |  |  |  |  |  |  |  |  |
| E2F2 | I not S | GPS1 | S and I | CDC25A | I not S |  |  |  |  |  |  |  |  |
| DLG7 | I not S | FLJ22624 | S and I | CDC2 | I not S |  |  |  |  |  |  |  |  |
| CDC7 | I not S | FANCG | S and I | CCNE2 | I not S |  |  |  |  |  |  |  |  |
| CDC45L | I not S | FANCD2 | S and I | CCNB2 | I not S |  |  |  |  |  |  |  |  |
| CDC25A | I not S | E2F3 | S and I | CCNA2 | I not S |  |  |  |  |  |  |  |  |
| CDC2 | I not S | CHAF1A | S and I | C20orf172 | I not S |  |  |  |  |  |  |  |  |
| CCNE2 | I not S | CENPF | S and I | BUB1B | I not S |  |  |  |  |  |  |  |  |
| CCNB2 | I not S | CDT1 | S and I | BUB1 | I not S |  |  |  |  |  |  |  |  |
| CCNA2 | I not S | CDK5RAP1 | S and I | BRCA2 | I not S |  |  |  |  |  |  |  |  |
| BUB1B | I not S | CDCA3 | S and I | ATF5 | I not S |  |  |  |  |  |  |  |  |
| BUB1 | I not S | CDC25C | S and I | ANLN | I not S |  |  |  |  |  |  |  |  |
| BRCA2 | I not S | CDC20 | S and I | ANAPC11 | I not S |  |  |  |  |  |  |  |  |
| ATF5 | I not S | CCNF | S and I |  |  |  |  |  |  |  |  |  |  |
| ANLN | I not S | CCNB1 | S and I |  |  |  |  |  |  |  |  |  |  |
