# Supplementary material for: Identification of Pro-Inflammatory Cytokines Associated with Muscle Invasive Bladder Cancer; The Roles of IL-5, IL-20, and IL-28A
Source: PLoS One. 2012 Sep 4;7(9):e40267. doi: 10.1371/journal.pone.0040267 (PMC3433484; doi:10.1371/journal.pone.0040267)
Supplement: Table S2 — Down-regulated genes in bladder tumor samples, compared to normal tissue samples. (DOCX) [file pone.0040267.s007.docx]

**Table S2. Down-regulated genes in bladder tumor samples, compared to normal tissue samples**

| response to wounding | | cell proliferation |  | apoptosis |  | immune response | | cytoskeleton |  | cell adhesion |  |
| --- | --- | --- | --- | --- | --- | --- | --- | --- | --- | --- | --- |
|  |  |  |  |  |  |  |  |  |  |  |  |
| **Gene symbol** | **Class** | **Gene symbol** | **Class** | **Gene symbol** | **Class** | **Gene symbol** | **Class** | **Gene symbol** | **Class** | **Gene symbol** | **Class** |
| ZFP36 | S not I | ZNF9 | S not I | ZBTB16 | S not I | WAS | S not I | WASPIP | S not I | ZFHX1B | S not I |
| WAS | S not I | ZMYND11 | S not I | ZAK | S not I | TNFSF12 | S not I | WAS | S not I | VCL | S not I |
| TPST1 | S not I | ZBTB16 | S not I | TNFSF12 | S not I | TLR1 | S not I | VIM | S not I | VCAM1 | S not I |
| TLR1 | S not I | ZAK | S not I | TNFRSF19 | S not I | TCF8 | S not I | VCL | S not I | TSPAN2 | S not I |
| STAT5A | S not I | TSPAN2 | S not I | TGFB3 | S not I | STAT5A | S not I | TUBB6 | S not I | TNC | S not I |
| STAB1 | S not I | TNFSF12 | S not I | STAT5A | S not I | SERPING1 | S not I | TPM2 | S not I | THBS2 | S not I |
| SERPING1 | S not I | TIMP2 | S not I | SOCS2 | S not I | RBM24 | S not I | TPM1 | S not I | TGFBI | S not I |
| SERPINA3 | S not I | TGFBI | S not I | SNRK | S not I | PAG1 | S not I | TMOD1 | S not I | TGFB1I1 | S not I |
| RBM24 | S not I | TGFB3 | S not I | SNCA | S not I | LY96 | S not I | TGFB1I1 | S not I | STAT5A | S not I |
| RAB27A | S not I | TGFB1I1 | S not I | SIAH2 | S not I | LY86 | S not I | STOM | S not I | STAB1 | S not I |
| PROS1 | S not I | TCF8 | S not I | SFRP1 | S not I | LILRA2 | S not I | SSPN | S not I | SSPN | S not I |
| PLSCR4 | S not I | STAT5A | S not I | RUNX3 | S not I | KLRK1 | S not I | SPIRE1 | S not I | SORBS1 | S not I |
| PLAT | S not I | ST8SIA1 | S not I | RTN4 | S not I | HLA-F | S not I | SORBS1 | S not I | SGCE | S not I |
| PLA2G4C | S not I | SLAMF1 | S not I | RRAGA | S not I | HLA-DRB3 | S not I | SGCE | S not I | SCARF2 | S not I |
| NTRK3 | S not I | RUNX3 | S not I | ROCK1 | S not I | HLA-DMB | S not I | SGCA | S not I | SCARB2 | S not I |
| NR3C1 | S not I | RARRES3 | S not I | RNF130 | S not I | GZMA | S not I | ROCK1 | S not I | ROR2 | S not I |
| NFATC4 | S not I | PMP22 | S not I | RABEP1 | S not I | FYB | S not I | PTP4A1 | S not I | PTPNS1 | S not I |
| LY96 | S not I | PGR | S not I | PTGIS | S not I | FCER1G | S not I | PRDM8 | S not I | PSCDBP | S not I |
| LY86 | S not I | PCAF | S not I | PRNP | S not I | FAS | S not I | PLS3 | S not I | PPFIA2 | S not I |
| KLRG1 | S not I | OSR2 | S not I | PRG1 | S not I | F3 | S not I | PLEKHC1 | S not I | PLEKHC1 | S not I |
| ITGB2 | S not I | NUMB | S not I | PRF1 | S not I | ETS1 | S not I | PDLIM3 | S not I | PKD1 | S not I |
| ITGAL | S not I | NCK1 | S not I | PLAGL1 | S not I | CX3CL1 | S not I | PCGF5 | S not I | PGM5 | S not I |
| GPR68 | S not I | MPL | S not I | P2RX1 | S not I | CLEC7A | S not I | PARVG | S not I | PCDH7 | S not I |
| GHRL | S not I | MAPRE2 | S not I | NTF3 | S not I | CLEC4A | S not I | PARVA | S not I | PCDH18 | S not I |
| FOS | S not I | LY86 | S not I | MOAP1 | S not I | CD97 | S not I | PALLD | S not I | PARVG | S not I |
| FCER1G | S not I | LRP1 | S not I | MAGEH1 | S not I | CD86 | S not I | NUDT21 | S not I | PARVA | S not I |
| F8 | S not I | KIAA1212 | S not I | LY86 | S not I | CD74 | S not I | NES | S not I | NRXN2 | S not I |
| F3 | S not I | JAK2 | S not I | LGALS1 | S not I | CD7 | S not I | NEIL2 | S not I | NLGN1 | S not I |
| F13A1 | S not I | ITGAL | S not I | KLRK1 | S not I | CD6 | S not I | MYOT | S not I | NELL2 | S not I |
| ELA2 | S not I | IGFBP7 | S not I | KIAA0367 | S not I | CD209 | S not I | MYOM1 | S not I | MGP | S not I |
| CYBB | S not I | IGFBP6 | S not I | JAK2 | S not I | CD14 | S not I | MYO1G | S not I | LAMB2 | S not I |
| CX3CL1 | S not I | GPNMB | S not I | ITGB2 | S not I | CCR2 | S not I | MYO1F | S not I | LAMA3 | S not I |
| CTGF | S not I | GLI2 | S not I | GZMH | S not I | CCL5 | S not I | MYL9 | S not I | LAMA2 | S not I |
| CLEC7A | S not I | GJA1 | S not I | GZMA | S not I | CCL4L1 | S not I | MAPRE2 | S not I | ITGB2 | S not I |
| CIAS1 | S not I | GHRL | S not I | GJA1 | S not I | CCL2 | S not I | MAP1LC3B | S not I | ITGAL | S not I |
| CD97 | S not I | GAS6 | S not I | GHRL | S not I | CCL19 | S not I | MAP1B | S not I | ISLR | S not I |
| CD14 | S not I | GAS1 | S not I | GADD45A | S not I | C7 | S not I | LYST | S not I | IGSF4B | S not I |
| CCR2 | S not I | ETS1 | S not I | FLJ11259 | S not I | C3 | S not I | LMOD1 | S not I | GPNMB | S not I |
| CCL5 | S not I | EMP3 | S not I | FCER1G | S not I | C1S | S not I | KIFAP3 | S not I | GNE | S not I |
| CCL4L1 | S not I | ELA2 | S not I | FAS | S not I | C1QC | S not I | KIF2 | S not I | FLRT2 | S not I |
| CCL2 | S not I | EDNRA | S not I | FAIM2 | S not I | C1QB | S not I | JAK2 | S not I | F8 | S not I |
| CCL19 | S not I | DDR2 | S not I | DNASE1L3 | S not I | C1QA | S not I | HINT1 | S not I | EMCN | S not I |
| C7 | S not I | CTTNBP2 | S not I | CRYAB | S not I | TLR7 | S and I | GYPC | S not I | EDIL3 | S not I |
| C3 | S not I | CSF1R | S not I | COL4A3 | S not I | HLA-DRB4 | S and I | GSN | S not I | DLL1 | S not I |
| C1S | S not I | COL4A3 | S not I | CIAS1 | S not I | HLA-DRA | S and I | GABARAPL1 | S not I | DDR2 | S not I |
| C1QC | S not I | CDV3 | S not I | CD74 | S not I | HLA-DQA1 | S and I | FYB | S not I | DCHS1 | S not I |
| C1QB | S not I | CDKN1C | S not I | CD14 | S not I | HLA-DPB1 | S and I | FNBP1 | S not I | CX3CL1 | S not I |
| C1QA | S not I | CDK6 | S not I | CCL2 | S not I | HLA-DPA1 | S and I | FLNC | S not I | CTGF | S not I |
| BDKRB2 | S not I | CD86 | S not I | CASP1 | S not I | FCER1A | S and I | FLNA | S not I | CPXM2 | S not I |
| AOX1 | S not I | CD74 | S not I | C8orf4 | S not I | CXCL12 | S and I | FLJ20366 | S not I | COL6A3 | S not I |
| AOAH | S not I | CD47 | S not I | BTG1 | S not I | CLEC10A | S and I | FKSG30 | S not I | COL6A2 | S not I |
| ANXA5 | S not I | CD33 | S not I | BNIP2 | S not I | CFI | S and I | EPB41L3 | S not I | COL6A1 | S not I |
| ANXA1 | S not I | CAV2 | S not I | APIP | S not I | CFD | S and I | DSTN | S not I | COL5A1 | S not I |
| ALOX5AP | S not I | CAV1 | S not I | API5 | S not I | CD8A | S and I | DOCK2 | S not I | COL4A6 | S not I |
| AIF1 | S not I | BTG1 | S not I | APBB2 | S not I | CCL14 | S and I | DOC1 | S not I | COL4A3 | S not I |
| ACVRL1 | S not I | BIN1 | S not I | ANXA5 | S not I | BCL2 | S and I | DMN | S not I | COL21A1 | S not I |
| TLR7 | S and I | ANXA1 | S not I | ANXA1 | S not I | B2M | S and I | DES | S not I | COL15A1 | S not I |
| SCUBE1 | S and I | ANG | S not I | ALDH1A3 | S not I | IL6ST | S and I | DCTN6 | S not I | COL14A1 | S not I |
| MGLL | S and I | AIF1 | S not I | ACTC | S not I | IL18RAP | S and I | CLIC4 | S not I | CNTNAP1 | S not I |
| FCER1A | S and I | ACVRL1 | S not I | LITAF | S and I | IL15 | S and I | CGNL1 | S not I | CNTN1 | S not I |
| FBLN5 | S and I | TGFBR2 | S and I | GULP1 | S and I | TLR3 | I not S | CALD1 | S not I | CLEC4A | S not I |
| F10 | S and I | STAT4 | S and I | ESR1 | S and I | IL7 | I not S | CALCOCO2 | S not I | CLDN5 | S not I |
| CXCL12 | S and I | SMAD4 | S and I | EDAR | S and I | CFH | I not S | C8orf72 | S not I | CDON | S not I |
| CFI | S and I | SERPINF1 | S and I | CD2 | S and I | IL7R | I not S | BIN1 | S not I | CDK6 | S not I |
| CFD | S and I | RPS4X | S and I | CASP12 | S and I | IL18R1 | I not S | ANXA1 | S not I | CDH17 | S not I |
| IL18RAP | S and I | RERG | S and I | BCL2 | S and I | IL16 | I not S | AMPH | S not I | CD97 | S not I |
| TLR3 | I not S | PTCH | S and I | TPT1 | I not S | IL11RA | I not S | AMOT | S not I | CD84 | S not I |
| EPHX2 | I not S | PPAP2A | S and I | PEG3 | I not S | IL10RA | I not S | ACTR10 | S not I | CD6 | S not I |
| CFH | I not S | PDGFRA | S and I | PDCD4 | I not S |  |  | ACTC | S not I | CD47 | S not I |
| IL7R | I not S | PDGFD | S and I | IL7 | I not S |  |  | UTRN | S and I | CD33 | S not I |
| IL7 | I not S | PDGFC | S and I | CFLAR | I not S |  |  | SYNE1 | S and I | CD209 | S not I |
| IL16ST | I not S | PAFAH1B1 | S and I | BTG2 | I not S |  |  | SORBS2 | S and I | CCL5 | S not I |
| IL18R1 | I not S | NDUFS4 | S and I | ARHGEF6 | I not S |  |  | PLEKHH2 | S and I | CCL4L1 | S not I |
| IL16 | I not S | NDN | S and I | IL7R | I not S |  |  | PDE4D | S and I | CCL2 | S not I |
| IL15 | I not S | LIFR | S and I | IL16ST | I not S |  |  | PCM1 | S and I | BOC | S not I |
| IL11RA | I not S | HHIP | S and I | IL18RAP | I not S |  |  | PAFAH1B1 | S and I | ATP2A2 | S not I |
| IL10RA | I not S | GLI1 | S and I | IL18R1 | I not S |  |  | NDN | S and I | AEBP1 | S not I |
| HLA-DRB4 | I not S | FGF9 | S and I | IL16 | I not S |  |  | MYOM2 | S and I | ACVRL1 | S not I |
| HLA-DRA | I not S | CGRRF1 | S and I | IL15 | I not S |  |  | MYH11 | S and I | SRPX | S and I |
| HLADQA1 | I not S | CD160 | S and I | IL11RA | I not S |  |  | JAK1 | S and I | SPON1 | S and I |
| HLA-DPB1 | I not S | CCND2 | S and I | IL10RA | I not S |  |  | IQGAP1 | S and I | NOPE | S and I |
| HLA-DPA1 | I not S | CCL14 | S and I | TLR7 | I not S |  |  | GABARAPL2 | S and I | MFAP4 | S and I |
| CLEC10A | I not S | BCL2 | S and I | HLA-DRB4 | I not S |  |  | EPB41L2 | S and I | LAMC3 | S and I |
| CD8A | I not S | AXIN2 | S and I | HLA-DRA | I not S |  |  | AKAP11 | S and I | JAM2 | S and I |
| CCL14 | I not S | ADAMTS8 | S and I | HLA-DQA1 | I not S |  |  | ACTG2 | S and I | ITGA8 | S and I |
| BCL2 | I not S | ADAMTS1 | S and I | HLA-DPB1 | I not S |  |  | ACTA2 | S and I | FBLN5 | S and I |
| B2M | I not S | IL6ST | S and I | HLA-DPA1 | I not S |  |  | TPT1 | I not S | EPDR1 | S and I |
|  |  | IL15 | S and I | FCER1A | I not S |  |  | TNNT3 | I not S | EMILIN1 | S and I |
|  |  | SCAP2 | I not S | CXCL12 | I not S |  |  | PKD2 | I not S | DPT | S and I |
|  |  | LDOC1 | I not S | CLEC10A | I not S |  |  | KIF13B | I not S | CXCL12 | S and I |
|  |  | IL7 | I not S | CFI | I not S |  |  | ANKRA2 | I not S | COL16A1 | S and I |
|  |  | BTG2 | I not S | CFD | I not S |  |  | IL7R | I not S | COL13A1 | S and I |
|  |  | IL7R | I not S | CD8A | I not S |  |  | IL7 | I not S | CDH11 | S and I |
|  |  | IL18RAP | I not S | CCL14 | I not S |  |  | IL6ST | I not S | CD2 | S and I |
|  |  | IL18R1 | I not S | B2M | I not S |  |  | IL18RAP | I not S | AMICA1 | S and I |
|  |  | IL16 | I not S | TLR3 | I not S |  |  | IL18R1 | I not S | PKD2 | I not S |
|  |  | IL11RA | I not S | CFH | I not S |  |  | IL16 | I not S | LPXN | I not S |
|  |  | IL10RA | I not S |  |  |  |  | IL15 | I not S | LGALS4 | I not S |
|  |  | TLR7 | I not S |  |  |  |  | IL11RA | I not S | IL7R | I not S |
|  |  | HLA-DRB4 | I not S |  |  |  |  | IL10RA | I not S | IL7 | I not S |
|  |  | HLA-DRA | I not S |  |  |  |  | TLR7 | I not S | IL6ST | I not S |
|  |  | HLA-DQA1 | I not S |  |  |  |  | HLA-DRB4 | I not S | IL18RAP | I not S |
|  |  | HLA-DPB1 | I not S |  |  |  |  | HLA-DRA | I not S | IL18R1 | I not S |
|  |  | HLA-DPA1 | I not S |  |  |  |  | HLA-DQA1 | I not S | IL16 | I not S |
|  |  | FCER1A | I not S |  |  |  |  | HLA-DPB1 | I not S | IL15 | I not S |
|  |  | CXCL12 | I not S |  |  |  |  | HLA-DPA1 | I not S | IL11RA | I not S |
|  |  | CLEC10A | I not S |  |  |  |  | FCER1A | I not S | IL10RA | I not S |
|  |  | CFI | I not S |  |  |  |  | CXCL12 | I not S | TLR7 | I not S |
|  |  | CFD | I not S |  |  |  |  | CLEC10A | I not S | HLA-DRB4 | I not S |
|  |  | CD8A | I not S |  |  |  |  | CFI | I not S | HLA-DRA | I not S |
|  |  | B2M | I not S |  |  |  |  | CFD | I not S | HLA-DQA1 | I not S |
|  |  | TLR3 | I not S |  |  |  |  | CD8A | I not S | HLA-DPB1 | I not S |
|  |  | CFH | I not S |  |  |  |  | CCL14 | I not S | HLA-DPA1 | I not S |
|  |  |  |  |  |  |  |  | BCL2 | I not S | CXCL12 | I not S |
|  |  |  |  |  |  |  |  | B2M | I not S | CLEC10A | I not S |
|  |  |  |  |  |  |  |  | TLR3 | I not S | CFI | I not S |
|  |  |  |  |  |  |  |  | CFH | I not S | CFD | I not S |
|  |  |  |  |  |  |  |  |  |  | CD8A | I not S |
|  |  |  |  |  |  |  |  |  |  | CCL14 | I not S |
|  |  |  |  |  |  |  |  |  |  | BCL2 | I not S |
|  |  |  |  |  |  |  |  |  |  | B2M | I not S |
|  |  |  |  |  |  |  |  |  |  | TLR3 | I not S |
|  |  |  |  |  |  |  |  |  |  | CFH | I not S |

**Table S2. Down-regulated genes in bladder tumor samples, compared to normal tissue samples**

| angiogenesis |  | wound healing | | DNA replication |  | DNA repair |  | regulation of progression | | cell cycle |  |
| --- | --- | --- | --- | --- | --- | --- | --- | --- | --- | --- | --- |
|  |  |  |  |  |  |  |  | through cell cycle |  |  |  |
| **Gene symbol** | **Class** | **Gene symbol** | **Class** | **Gene symbol** | **Class** | **Gene symbol** | **Class** | **Gene symbol** | **Class** | **Gene symbol** | **Class** |
| TNFSF12 | S not I | WAS | S not I | NFIX | S not I | NEIL2 | S not I | ZMYND11 | S not I | ZMYND11 | S not I |
| STAB1 | S not I | SERPING1 | S not I | NFIA | S not I | GADD45A | S not I | ZAK | S not I | ZAK | S not I |
| GHRL | S not I | RAB27A | S not I | KIAA1212 | S not I | CCNH | S not I | WEE1 | S not I | WEE1 | S not I |
| EPAS1 | S not I | PROS1 | S not I | GLI2 | S not I | GTF2H5 | S and I | STAT5A | S not I | UBB | S not I |
| EMCN | S not I | PLSCR4 | S not I | CTGF | S not I | CRY2 | S and I | SASH1 | S not I | TCF7L1 | S not I |
| CTGF | S not I | PLAT | S not I | GLI1 | S and I | BTG2 | I not S | RUNX3 | S not I | TACC1 | S not I |
| COL4A3 | S not I | NTRK3 | S not I | IL7R | I not S | IL7R | I not S | PPP3CB | S not I | STAT5A | S not I |
| COL15A1 | S not I | F8 | S not I | IL7 | I not S | IL7 | I not S | PLAGL1 | S not I | SIAH2 | S not I |
| BTG1 | S not I | F3 | S not I | IL6ST | I not S | IL6ST | I not S | PCAF | S not I | SASH1 | S not I |
| BMP4 | S not I | F13A1 | S not I | IL18RAP | I not S | IL18RAP | I not S | JAK2 | S not I | RUNX3 | S not I |
| ANPEP | S not I | ANXA5 | S not I | IL18R1 | I not S | IL18R1 | I not S | HIC1 | S not I | RGS2 | S not I |
| ANG | S not I | ACVRL1 | S not I | IL16 | I not S | IL16 | I not S | HBP1 | S not I | PTP4A1 | S not I |
| AMOT | S not I | SCUBE1 | S and I | IL15 | I not S | IL15 | I not S | GAS1 | S not I | PPP3CB | S not I |
| ACVRL1 | S not I | FBLN5 | S and I | IL11RA | I not S | IL11RA | I not S | GADD45A | S not I | PPP1CB | S not I |
| SERPINF1 | S and I | F10 | S and I | IL10RA | I not S | IL10RA | I not S | DIRAS3 | S not I | PLAGL1 | S not I |
| FGF9 | S and I | IL7R | I not S | TLR7 | I not S | TLR7 | I not S | CDKN1C | S not I | PCAF | S not I |
| IL7R | I not S | IL7 | I not S | HLA-DRB4 | I not S | HLA-DRB4 | I not S | CDK8 | S not I | NEK1 | S not I |
| IL7 | I not S | IL6ST | I not S | HLA-DRA | I not S | HLA-DRA | I not S | CDK6 | S not I | MAPRE2 | S not I |
| IL6ST | I not S | IL18RAP | I not S | HLA-DQA1 | I not S | HLA-DQA1 | I not S | CCNH | S not I | KIAA0367 | S not I |
| IL18RAP | I not S | IL18R1 | I not S | HLA-DPB1 | I not S | HLA-DPB1 | I not S | BMP4 | S not I | JAK2 | S not I |
| IL18R1 | I not S | IL16 | I not S | HLA-DPA1 | I not S | HLA-DPA1 | I not S | BIN1 | S not I | HIC1 | S not I |
| IL16 | I not S | IL15 | I not S | FCER1A | I not S | FCER1A | I not S | AXL | S not I | HBP1 | S not I |
| IL15 | I not S | IL11RA | I not S | CXCL12 | I not S | CXCL12 | I not S | ARHGAP20 | S not I | GPR132 | S not I |
| IL11RA | I not S | IL10RA | I not S | CLEC10A | I not S | CLEC10A | I not S | APBB2 | S not I | GFI1 | S not I |
| IL10RA | I not S | TLR7 | I not S | CFI | I not S | CFI | I not S | AIF1 | S not I | GAS1 | S not I |
| TLR7 | I not S | HLA-DRB4 | I not S | CFD | I not S | CFD | I not S | RPS4X | S and I | GADD45A | S not I |
| HLA-DRB4 | I not S | HLA-DRA | I not S | CD8A | I not S | CD8A | I not S | RECK | S and I | DUSP1 | S not I |
| HLA-DRA | I not S | HLA-DQA1 | I not S | CCL14 | I not S | CCL14 | I not S | PTCH | S and I | DIRAS3 | S not I |
| HLA-DQA1 | I not S | HLA-DPB1 | I not S | BCL2 | I not S | BCL2 | I not S | PDGFD | S and I | CDKN1C | S not I |
| HLA-DPB1 | I not S | HLA-DPA1 | I not S | B2M | I not S | B2M | I not S | PDGFC | S and I | CDK8 | S not I |
| HLA-DPA1 | I not S | FCER1A | I not S | TLR3 | I not S | TLR3 | I not S | NDN | S and I | CDK6 | S not I |
| FCER1A | I not S | CXCL12 | I not S | CFH | I not S | CFH | I not S | FGF9 | S and I | CCNH | S not I |
| CXCL12 | I not S | CLEC10A | I not S |  |  |  |  | CGRRF1 | S and I | CALM1 | S not I |
| CLEC10A | I not S | CFI | I not S |  |  |  |  | CCND2 | S and I | BMP4 | S not I |
| CFI | I not S | CFD | I not S |  |  |  |  | BCL2 | S and I | BIN1 | S not I |
| CFD | I not S | CD8A | I not S |  |  |  |  | RBL2 | I not S | AXL | S not I |
| CD8A | I not S | CCL14 | I not S |  |  |  |  | PKD2 | I not S | ARHGAP20 | S not I |
| CCL14 | I not S | BCL2 | I not S |  |  |  |  | PDCD4 | I not S | APBB2 | S not I |
| BCL2 | I not S | B2M | I not S |  |  |  |  | IL7R | I not S | ANXA1 | S not I |
| B2M | I not S | TLR3 | I not S |  |  |  |  | IL7 | I not S | AIF1 | S not I |
| TLR3 | I not S | CFH | I not S |  |  |  |  | IL6ST | I not S | RPS4X | S and I |
| CFH | I not S |  |  |  |  |  |  | IL18RAP | I not S | RECK | S and I |
|  |  |  |  |  |  |  |  | IL18R1 | I not S | PTCH | S and I |
|  |  |  |  |  |  |  |  | IL16 | I not S | PDGFD | S and I |
|  |  |  |  |  |  |  |  | IL15 | I not S | PDGFC | S and I |
|  |  |  |  |  |  |  |  | IL11RA | I not S | PCNP | S and I |
|  |  |  |  |  |  |  |  | IL10RA | I not S | PAM | S and I |
|  |  |  |  |  |  |  |  | TLR7 | I not S | PAFAH1B1 | S and I |
|  |  |  |  |  |  |  |  | HLA-DRB4 | I not S | NDN | S and I |
|  |  |  |  |  |  |  |  | HLA-DRA | I not S | GSPT2 | S and I |
|  |  |  |  |  |  |  |  | HLA-DQA1 | I not S | FGF9 | S and I |
|  |  |  |  |  |  |  |  | HLA-DPB1 | I not S | CGRRF1 | S and I |
|  |  |  |  |  |  |  |  | HLA-DPA1 | I not S | CCPG1 | S and I |
|  |  |  |  |  |  |  |  | FCER1A | I not S | CCND2 | S and I |
|  |  |  |  |  |  |  |  | CXCL12 | I not S | BCL2 | S and I |
|  |  |  |  |  |  |  |  | CLEC10A | I not S | RBL2 | I not S |
|  |  |  |  |  |  |  |  | CFI | I not S | PKD2 | I not S |
|  |  |  |  |  |  |  |  | CFD | I not S | PDCD4 | I not S |
|  |  |  |  |  |  |  |  | CD8A | I not S | IL7R | I not S |
|  |  |  |  |  |  |  |  | CCL14 | I not S | IL7 | I not S |
|  |  |  |  |  |  |  |  | B2M | I not S | IL6ST | I not S |
|  |  |  |  |  |  |  |  | TLR3 | I not S | IL18RAP | I not S |
|  |  |  |  |  |  |  |  | CFH | I not S | IL18R1 | I not S |
|  |  |  |  |  |  |  |  |  |  | IL16 | I not S |
|  |  |  |  |  |  |  |  |  |  | IL15 | I not S |
|  |  |  |  |  |  |  |  |  |  | IL11RA | I not S |
|  |  |  |  |  |  |  |  |  |  | IL10RA | I not S |
|  |  |  |  |  |  |  |  |  |  | TLR7 | I not S |
|  |  |  |  |  |  |  |  |  |  | HLA-DRB4 | I not S |
|  |  |  |  |  |  |  |  |  |  | HLA-DRA | I not S |
|  |  |  |  |  |  |  |  |  |  | HLA-DQA1 | I not S |
|  |  |  |  |  |  |  |  |  |  | HLA-DPB1 | I not S |
|  |  |  |  |  |  |  |  |  |  | HLA-DPA1 | I not S |
|  |  |  |  |  |  |  |  |  |  | FCER1A | I not S |
|  |  |  |  |  |  |  |  |  |  | CXCL12 | I not S |
|  |  |  |  |  |  |  |  |  |  | CLEC10A | I not S |
|  |  |  |  |  |  |  |  |  |  | CFI | I not S |
|  |  |  |  |  |  |  |  |  |  | CFD | I not S |
|  |  |  |  |  |  |  |  |  |  | CD8A | I not S |
|  |  |  |  |  |  |  |  |  |  | CCL14 | I not S |
|  |  |  |  |  |  |  |  |  |  | B2M | I not S |
|  |  |  |  |  |  |  |  |  |  | TLR3 | I not S |
|  |  |  |  |  |  |  |  |  |  | CFH | I not S |
