# Supplementary material for: Identification of Pro-Inflammatory Cytokines Associated with Muscle Invasive Bladder Cancer; The Roles of IL-5, IL-20, and IL-28A
Source: PLoS One. 2012 Sep 4;7(9):e40267. doi: 10.1371/journal.pone.0040267 (PMC3433484; doi:10.1371/journal.pone.0040267)
Supplement: Table S3 — Up-regulated genes in muscle invasive bladder cancer (MIBC) samples, compared to non-muscle invasive bladder cancer (NMIBC) samples. (DOCX) [file pone.0040267.s008.docx]

**Table S3. Up-regulated genes in muscle invasive bladder cancer (MIBC) samples, compared to non-muscle invasive bladder cancer (NMIBC) samples**

| Category | Molecules |
| --- | --- |
| Cancer | NES, CCNB2, IL6, GPSM2, CSE1L, ADA, GNA13, PTGIS, EGLN2, HEYL, PDIA2, CCNE2, FERMT2, ANGPTL2, PDLIM7, UBE2C, KIF20A, FHL1, EG:14199, GADD45B, CALM1, PRDX1, TTK, SYNM, TUBB2B, C1R, PALLD, ARRB1, MXD1, ANTXR2, CD27, EXO1, EG:26909, SLC12A8, FCGR2A, EMP3, BUB1, EG:100307076, GPR183, NR4A1, KIF23, SOCS3, FOXD1, RARRES2, IDI1, TMEM45A, JUND, ZWINT, ACTG2, SEC63, EG:11231, E2F2, IL8, THBS1, KDM2B, CDKN2D, MUC13, RAD54L, AURKA, CLIC2, JAM3, PPAT, SULF1, LOX, TPM1, EG:22003, MOK, RAB23, SNRPG, IFITM3, CTLA4, HDGF, FOXL2, COL6A1, MICB, HSPB6, TRIP13, TACC3, HPD, COL6A2, RACGAP1, PRC1, EG:233406, PLAUR, APOLD1, ERG, MPP1, TUBB6, NFE2, CKS1B, BRCA2, CDKN1B, CXCL2, PIM2, PRUNE2, MCM6, C1orf38, CTGF, FAM20C, MKI67, SERPINB2, MIA, MYLK, CTSL2, NR4A3, ASB5, LYVE1, DNMT1, SFRP4, ADORA3, CLC, MT1X, CTNNAL1, ITGA5, HBEGF, RAB31, AP2S1, RBM28, HSD17B6, SPON2, UBE2G2, ISLR, FOXE1, SGOL1, SETBP1, NRP1, EG:18186, COL3A1, AKAP12, FYN, ITGA2B, FBXL7, OSR2, ZFP36, CDT1, CUL1, TWIST1, CDH11, FAM54A, STOM, NFIL3, JUN, LZTS1, LETM2, ORC1, EG:18392, GRIN3B, TNC, KRT6B, EGR1, COL12A1, SPC25, EG:100144563, MED30, PLK1, RAMP1, SLIT2, P2RX7, RELN, AXL, TRIM37, F3, CDK1, RRM1, DENND2A, PI3, DHCR24, GMPS, MBNL1, IL2RA, KCNMB1, TM4SF1, EHBP1, MYH11, PBK, BIRC5, YWHAQ, IL7R, OR4Q3, ALDH1A1, OSM, TSPAN4, STX11, MT1G, FST, COL4A1, POLE2, RRAD, PDGFRL, C1S, RGS4, CD97, PAPSS2, COL6A3, FAM46C, RASSF4, TGFB3, FBN1, IDO1, CD14, TK1, PDLIM3, SFRP2, GEM, DDX39A, NUDT3, ACTA2, MRGPRF, LMOD1, NFKBIA, CALD1, PHGDH, SPHK1, CDCA5, ACTC1, RASSF1, EMILIN1, RUNX3, UHRF1, SPRR1B, RYR2, HYAL1, TPM2, CEBPB, EG:1051, NXT1, TNFRSF13C, LYAR, PARP1, EMP1, COL1A1, IL1B, GAP43, SNCAIP, DPYSL2, DBN1, MYH10, CRYAB, GLI2, HMMR, SERPINA3, PMP2, EG:100334189, SPI1, EG:20375, DOCK11, SOD2, GAL, MT2A, KIF1A, IFITM2, LUM, NEK6, CAV1, MTHFD2, MGP, SCHIP1, FOSL1, SERPINE1, TYMS, RHEB, PODN, PLN, ESPL1, CDCA8, MFAP5, GYPC, TPM3, TBX4, PITX2, PTP4A1, DES, RASL12, MAPK8IP1, ATF2, PCP4, SEC61G, NCF1, RUNX1T1, CTSL1, SCN1B, GPR65, SIK1, TFRC, CD86, ITGA1, BCL2A1, SAMD4A, PIP4K2A, DEK, RAP2A, APOE, ICAM1, MFGE8, GLIPR1, KSR1, VRK1, CDCA7, DUSP5, CCL2, CD38, KIF2C, NMU, RNASE2, SERTAD2, MGC24103, NRP2, ENTPD1, VIM, MLF1, HIST1H3A, NR4A2, ZFPM2, FLNC, TMEM106C, TAGLN, CST7, MAP3K8, NRIP1, DLGAP5, MMP3, KIF1B, FGL2, PTTG1, SCUBE3, BNC2, SMC4, TLR8, TYMP, RNASE1, CD163, RBP1, OLR1, TCEB1, STAB2, CEBPG, NNMT, SMS, B4GALNT1, STMN1, PIM1, RUNX2, HOXC6, SCRG1, PHF16, CDKN1C, PDZRN3, KLF11, STK17B, KIFC1, ITGB1BP2, KCNJ8, ATF3, FSTL3, CNP, CDK14, PTGDS, ADAMTS9, BUB1B, DCHS1, FPR1, FOSB, NPC1, ICOS, PTPN11, DUSP1, PPP1R12B, GOLGA5, CD248, FAM83D, IRF8, SMTN, ELMO1, PTGFR, LIX1L, SLC1A4, CDC20, SRPX, CHST7, RECK, CHRDL2, DMD, CCL3, CHRM3, SLC2A3, HIC1, CDC25B, NEK2, OLFML2A, LILRB3, MCAM, NAMPT, NUSAP1, C12orf24, UPP1, MT1H, FOXM1, SNAP25, SERPINE2, PMP22, LAMA4, P2RY6, CDH17, MSH6, C15orf48, BDKRB1, ISG20, EME1, CENPF, GLT8D2, SOCS1, FTL, MYL6, SGK1, CDC7, EG:12545, CDKN2C, ADARB1, ADAMTS2, CXCL10, BCAT1, ENAH, CXCL13, CDKN3, PRDM1, OSMR, CHAMP1, PDGFRB, TIMP2, EG:21858, SYDE1, CD19, TOPBP1, ZIC2, SNAI1, LAG3, DEFA1, CACNA1C, THY1, MMP2, ANLN, UBE2S, IER3, COLEC12, CDH2, AQP9, RFC4, PSMB2, BHLHE40, ZAK, E2F1, IL10RA, DTNA, HSPB7, LRP8, SFRP1, CYR61, MT1E, HMGB2, MSC, FN1, DEPDC1, MXRA8, WWTR1, COL4A2, DCLK1, LEFTY2, VCAN, CHEK1, UCHL1, S100A7, TRIB1, MMP25, SLC2A14, PRRX1, ANXA5, GAS1, EG:14451, TOP2A, MCM10, EG:307126, CACNB2, COL18A1, FGF7, SLC7A6, ITGB1, RGS2, EG:19735, CTSK, TGFBI, TYROBP, MOXD1, CDC6, EG:23834, ST6GALNAC5, HMHA1, POLD1, YBX2, ITGB2, ABL2, MCM2, S100A9, KIF20B, CNN1, EG:1264, CENPA, ZEB2, PEA15, CCL21, MAFB, COL15A1, ADORA2A, FOXC2, ATP6V1B1, CLDN3, RGS1, INSIG1, DIAPH3, IL32, XPO5, CXCL1, MELK, AURKB, TUBB, CDA, POLD3, DTX1, CRIP1, NUDT11, PRRX2, EZH2, DSTN, IFI30, POSTN, ECT2, BRCA1, FAM65B, CDC25A, PDK4, KIF14, TUBB3, SPP1, EG:20750, GGH, CDK6, ENO2, IL24, TPX2, PDLIM4, STC1, MPP3, CEBPD, TMOD1, CEP55, KIF11, RFC3, TEAD4, HIST2H2BE, ERN1, DYNC1I1, TNFAIP3, ACOT7, COL1A2, ZNF295, HMOX1, SERPINB9, FLNA, ANXA1, CIT, MMP11, S100A8, PRNP, IRF4, IL13RA1, CXCR4, RELB, ADCY3, CBX2, BCL3, USP1, S100A12, DNM1, WARS, CYTIP, KDELR2, EDNRA, PDE5A, EFEMP1, UBC, ELK1, MMP9, LGALS1 |
| Reproductive System Disease | CRYAB, NES, GAS6, HMMR, ACCN3, CCNB2, PMP2, EG:100334189, IL6, DOCK11, SOD2, GAL, MT2A, CSE1L, IFITM2, LUM, NEK6, CAV1, MTHFD2, MGP, SCHIP1, FOSL1, SERPINE1, GUCY1B3, PTGIS, TYMS, EGLN2, HEYL, PLN, ESPL1, MFAP5, FERMT2, TPM3, PITX2, RASL12, DES, PTP4A1, ATF2, PCP4, RUNX1T1, CTSL1, GPR65, TFRC, SIK1, ITGA1, BCL2A1, SAMD4A, UBE2C, KIF20A, APOE, FHL1, EG:14199, ICAM1, GADD45B, CALM1, MFGE8, TTK, GLIPR1, KSR1, TUBB2B, SYNM, C1R, PALLD, CDCA7, CCL2, MXD1, ANTXR2, KIF2C, EXO1, EG:26909, NMU, RNASE2, MGC24103, ENTPD1, NRP2, CCL23, EMP3, GREM1, VIM, GPR183, BUB1, EG:100307076, ZFPM2, NR4A2, FLNC, TAGLN, NR4A1, TMEM106C, MAP3K8, NRIP1, SOCS3, FOXD1, YWHAH, MMP3, KIF1B, FGL2, PTTG1, BNC2, SMC4, MT1M, TYMP, PIP5K1B, CD163, TCEB1, SMS, PIM1, RUNX2, CCNK, HOXC6, JUND, SCRG1, ACTG2, PDZRN3, KIFC1, KLF11, IL8, ATF3, FSTL3, THBS1, CNP, RAD54L, PTGDS, AURKA, BUB1B, DCHS1, FOSB, ICOS, CLIC2, PTPN11, JAM3, DUSP1, PPP1R12B, SMTN, PPAT, PTGFR, TPM1, EG:22003, SULF1, SLC1A4, CDC20, CHST7, RECK, CHRDL2, DMD, CTLA4, CHRM3, SLC2A3, HIC1, CDC25B, FOXL2, COL6A1, NEK2, MICB, OLFML2A, MCAM, NAMPT, HSPB6, TRIP13, COL6A2, RACGAP1, PRC1, EG:233406, UPP1, PLAUR, MT1H, FOXM1, APOLD1, ERG, PMP22, TUBB6, LAMA4, CKS1B, MSH6, BRCA2, CDKN1B, CXCL2, ISG20, IL11, PRUNE2, GLT8D2, SOCS1, MCM6, CADM3, C1orf38, CTGF, MYL6, SGK1, CDC7, EG:12545, MKI67, ADARB1, CXCL10, CTSL2, MYLK, BCAT1, NR4A3, LTB4R, ENAH, CXCL13, ASB5, PRDM1, LYVE1, DNMT1, TIMP2, EG:21858, PDGFRB, SFRP4, TOPBP1, DCN, MT1X, CTNNAL1, THY1, CACNA1C, HBEGF, MMP2, UBE2S, IER3, AP2S1, HSD17B6, SPON2, RBM28, UBE2G2, CDH2, RFC4, BHLHE40, ZAK, E2F1, DTNA, LRP8, HSPB7, SFRP1, SETBP1, CYR61, MT1E, COL3A1, OSR2, FN1, FBXL7, ZFP36, DEPDC1, MXRA8, WWTR1, LEFTY2, CDH11, FAM54A, VCAN, CHEK1, UCHL1, TRIB1, NFIL3, JUN, ANXA5, GAS1, EG:14451, TOP2A, CACNB2, LETM2, TNFRSF1B, CAV2, COL18A1, FGF7, SLC7A6, ITGB1, RGS2, EG:19735, GRIN3B, CNTN1, TNC, TGFBI, KRT6B, MOXD1, ACTB, EGR1, CDC6, EG:23834, SPC25, EG:100144563, HMHA1, PLK1, P2RX7, RAMP1, SLIT2, AXL, RELN, POLD1, TRIM37, CDK1, RRM1, YBX2, DENND2A, PI3, MCM2, DHCR24, S100A9, CNN1, EG:1264, CENPA, PEA15, CCL21, IL2RA, KCNMB1, COL15A1, EHBP1, CLDN3, ATP6V1B1, FOXC2, RGS1, IL32, CXCL1, MYH11, TUBB, AURKB, PBK, CDA, BIRC5, IL7R, YWHAQ, OR4Q3, G0S2, ALDH1A1, EZH2, POSTN, IFI30, TSPAN4, BRCA1, ECT2, FAM65B, PDK4, MT1G, TUBB3, FST, SPP1, EG:20750, COL4A1, POLE2, C1S, CDK6, CD97, TPX2, PAPSS2, STC1, PDLIM4, COL6A3, CEBPD, TGFB3, IDO1, FBN1, CCL18, KIF11, TK1, PDLIM3, GEM, SFRP2, HIST2H2BE, DYNC1I1, ACTA2, MRGPRF, LMOD1, ZNF295, COL1A2, HMOX1, SERPINB9, FLNA, CALD1, ANXA1, CIT, S100A8, MMP11, ACTC1, EMILIN1, RASSF1, RUNX3, UHRF1, CXCR4, RELB, ADCY3, HYAL1, TPM2, CEBPB, EG:1051, PARP1, COL1A1, CYTIP, PDE5A, EDNRA, SNCAIP, MMP9 |
| Gastrointestinal Disease | DPYSL2, CCNB2, SERPINA3, IL6, GPSM2, SOD2, MT2A, CSE1L, IFITM2, NEK6, CAV1, SERPINE1, PTGIS, TYMS, CDCA8, PODN, ANGPTL2, TBX4, PITX2, DES, CTSL1, SCN1B, GPR65, CD86, TFRC, UBE2C, KIF20A, APOE, ICAM1, PRDX1, TTK, VRK1, C1R, COL16A1, PALLD, CDCA7, DUSP5, C5AR1, CCL2, EXO1, EG:26909, SERTAD2, SLC12A8, VIM, HIST1H3A, NR4A1, NRIP1, SOCS3, DLGAP5, IDI1, MMP3, PTTG1, TLR8, TYMP, RBP1, OLR1, STAB2, NNMT, B4GALNT1, HOXC6, CCL8, PHF16, ZWINT, PDZRN3, SEC63, EG:11231, KIFC1, IL8, KCNJ8, ATF3, FSTL3, MUC13, RAD54L, CDK14, AURKA, PDE4B, BUB1B, NPC1, PTPN11, CD248, FAM83D, SRPX, MOK, DMD, CTLA4, IFITM3, CHRM3, SLC2A3, HDGF, CDC25B, NEK2, COL6A1, MICB, MCAM, NAMPT, TRIP13, NUSAP1, HPD, COL6A2, C12orf24, CD6, PLAUR, MT1H, FOXM1, SERPINE2, PMP22, LAMA4, CKS1B, CDH17, MSH6, BRCA2, COL11A1, C15orf48, CDKN1B, CXCL2, BDKRB1, ISG20, CENPF, SOCS1, FTL, CTGF, SGK1, CDC7, EG:12545, CDKN2C, MKI67, CXCL10, MYLK, ENAH, CXCL13, COL10A1, CDKN3, OSMR, PDGFRB, TIMP2, EG:21858, CLC, MT1X, HBEGF, THY1, ITGA5, DEFA1, ANLN, MMP2, UBE2S, RAB31, HSD17B6, SPON2, COLEC12, ISLR, CDH2, RFC4, LRP8, SFRP1, SGOL1, CYR61, MT1E, HMGB2, COL3A1, NRP1, EG:18186, MSC, FN1, TWIST1, COL4A2, DCLK1, COL5A1, UCHL1, JUN, MMP25, PDE3B, LZTS1, SLC2A14, PRRX1, TOP2A, MCM10, EG:307126, COL18A1, MNDA, ORC1, EG:18392, TNC, TYROBP, COL12A1, ACTB, SPC25, EG:100144563, CDC6, EG:23834, MED30, PLK1, SLIT2, AXL, F3, POLD1, RRM1, GMPS, CENPA, CCL21, MAFB, COL15A1, CLDN3, TM4SF1, INSIG1, XPO5, CXCL1, MELK, TUBB, PBK, CDA, BIRC5, IL7R, POLD3, CRIP1, PRRX2, DSTN, POSTN, ECT2, BRCA1, TUBB3, SPP1, EG:20750, FST, COL4A1, POLE2, RRAD, C1S, PDGFRL, GGH, RGS4, TPX2, PAPSS2, COL6A3, MPP3, CEBPD, TGFB3, CD14, IDO1, CCL18, CEP55, TK1, PDLIM3, TEAD4, SFRP2, DDX39A, NUDT3, ACTA2, ACOT7, COL1A2, HMOX1, ANXA1, CALD1, PHGDH, S100A8, CDCA5, MMP11, RASSF1, PRNP, RUNX3, COL5A2, CXCR4, RELB, TPM2, CEBPB, EG:1051, USP1, LYAR, EMP1, COL1A1, WARS, IL1B, PDE5A, EFEMP1, MMP9, LGALS1 |
| Cellular Movement | DPYSL2, MYH10, NES, HMMR, IL13RA2, CTSG, SERPINA3, IL6, SOD2, CSE1L, NEK6, MGP, CAV1, FOSL1, GNA13, SERPINE1, PODN, FERMT2, TPM3, PITX2, PTP4A1, CTSL1, CD86, ITGA1, KIF20A, DEK, CD99, APOE, RAP2A, FHL1, EG:14199, ICAM1, CALM1, SYNM, TUBB2B, TGM2, ARRB1, CCL2, DOCK2, CD38, FAP, RNASE2, CCL23, VIM, PVR, CCL11, SEMA3A, TAGLN, MAP3K8, KIF23, SOCS3, RARRES2, LIF, MMP3, PTTG1, SCUBE3, GHSR, OLR1, STMN1, PIM1, JUND, CCL8, KIFC1, IL8, ATF3, THBS1, CNP, KIF4A, CDK14, AURKA, MAPK12, FPR1, ICOS, PTPN11, JAM3, SGPP1, ELMO1, SIRPA, DCBLD2, SULF1, LOX, TPM1, EG:22003, CDC20, RECK, CCL3, CLIC4, CHRM3, HDGF, CDC25B, MCAM, NUSAP1, ST8SIA1, RACGAP1, PRC1, EG:233406, PLAUR, FOXM1, SERPINE2, PMP22, NRTN, SLC22A16, BRCA2, CDKN1B, CXCL2, BDKRB1, C5orf13, IL11, ITGAX, CTGF, MIA, MYLK, CXCL10, CXCL13, LYVE1, TIMP2, EG:21858, PDGFRB, ADORA3, PDE2A, DCN, SNAI1, DEFA1, ITGA5, HBEGF, THY1, MMP2, ANLN, IER3, S1PR3, NOD2, CDH2, LRP8, SFRP1, CYR61, NRP1, EG:18186, HMGB2, FYN, FN1, WWTR1, TWIST1, COL4A2, VCAN, CDH11, PDXP, S100A7, TRIB1, JUN, TOP2A, COL18A1, TNFRSF1B, FGF7, CD53, ITGB1, CTSK, TNC, EGR1, ST6GALNAC5, PLK1, SLIT2, AXL, RELN, F3, CDK1, ITGB2, S100A9, WAS, KIF20B, ZEB2, CCL21, ADORA2A, CLDN3, GAB2, RGS1, DIAPH3, CXCL1, ACVRL1, PBK, PLA2G7, BIRC5, POSTN, OSM, ECT2, ARHGAP24, S100A10, KIF14, FST, SPP1, EG:20750, COL4A1, RGS4, CD97, STC1, TGFB3, CD14, CCL3L1/CCL3L3, CEP55, SFRP2, MAPK11, HMOX1, ROR2, CCL13, NFKBIA, FLNA, ANXA1, CIT, SPHK1, S100A8, GTPBP4, RASSF1, PRNP, CXCR4, ASAP1, DNM1, COL1A1, WARS, ASAP2, IL1B, NCKAP1L, ELK1, MMP9, LGALS1 |
| Cellular Growth and Proliferation | DBN1, GLI2, NES, GAS6, HMMR, IL13RA2, IL6, SPI1, EG:20375, FADS3, DYRK3, NCK2, EGR2, SOD2, GAL, GNLY, MAD2L2, MT2A, CSE1L, LUM, NEK6, CAV1, MGP, FOSL1, AIF1, EG:11629, SERPINE1, TYMS, GNE, PDIA5, EGLN2, CCNE2, CDCA8, ESPL1, PODN, MAFF, DES, PTP4A1, CDCA4, SEC61G, CTSL1, RUNX1T1, GFI1, TFRC, CD86, ITGA1, BCL2A1, ADCYAP1, EG:11516, UBE2C, KIF20A, CD99, APOE, FHL1, EG:14199, SLAMF1, ICAM1, GADD45B, CALM1, PRDX1, TTK, SYNM, TGM2, DUSP5, CDCA7, CCL2, MXD1, CD38, KIF2C, SERTAD2, ENTPD1, EMP3, CCL23, CCL11, BUB1, EG:100307076, NR4A2, LMO3, NR4A1, TCF19, NRIP1, KIF23, DLGAP5, SOCS3, LIF, PTTG1, SCUBE3, CD163, BIN1, TSC22D3, STMN1, SAE1, RUNX2, PIM1, HOXC6, JUND, CDKN1C, KLF11, EIF5A2, IL8, ATF3, FSTL3, THBS1, LAMA2, CDKN2D, CDK14, AURKA, MAPK12, BUB1B, DCHS1, ATAD2, ICOS, PTPN11, DUSP1, HAVCR2, CD248, SLC7A7, MAPRE2, IRF8, PPAT, SIRPA, PMEPA1, DCBLD2, TPM1, EG:22003, LOX, SULF1, SUMO3, CCL3, CHRM3, CTLA4, HDGF, CDC25B, COL6A1, NEK2, MCAM, NAMPT, PREB, ST3GAL2, CDC25C, ST8SIA1, COL6A2, CD6, PLAUR, NFATC4, FOXM1, MT1A, DEGS1, SERPINE2, ERG, BTK, TYRP1, PMP22, PSMD2, NFE2, CKS1B, BRCA2, CDKN1B, CXCL2, BDKRB1, ISG20, IL11, PIM2, SOCS1, FTL, CTGF, SGK1, CDC7, EG:12545, SRF, CDKN2C, MKI67, RBL1, CD37, SERPINB2, CXCL10, MIA, NR4A3, BCAT1, PEG10, CDKN3, OSMR, ICOSLG, TIMP2, EG:21858, PDGFRB, SFRP4, CD19, POU4F1, MT1X, DCN, DEFA1, HBEGF, ITGA5, MMP2, ANLN, IER3, S1PR3, CDH2, ASGR1, PSMB2, E2F1, IL10RA, SFRP1, CYR61, NRP1, EG:18186, RCVRN, NUDT1, HMGB2, TCF4, ITGA2B, FN1, ZFP36, CDT1, WWTR1, CUL1, TWIST1, FOLR2, GPC4, COL4A2, CPSF4, CDH11, VCAN, CHEK1, UCHL1, S100A7, TRIB1, YY1AP1, JUN, LZTS1, MEG3, CAV2, FKBP5, COL18A1, TNFRSF1B, FGF7, MNDA, ITGB1, CDC45, SPEG, TGFBI, ACTB, EGR1, CDC6, EG:23834, PPP1R15A, PLK1, SLIT2, AXL, COPS3, F3, TSLP, CDK1, RRM1, PSMC1, KIF15, MCM2, WAS, CNN1, EG:1264, FCER1G, CCL21, IL2RA, GAB2, PHLDA1, INSIG1, CXCL1, UBE2V2, ACVRL1, MELK, AURKB, PBK, CDA, BIRC5, YWHAQ, IL7R, ALDH1A1, CRIP1, RORA, EZH2, POSTN, IFI30, MRAS, OSM, ECT2, BRCA1, ARHGAP24, CDC25A, S100A10, TUBB3, STIL, FST, COL4A1, SPP1, EG:20750, RRAD, CDK6, RGS4, FZD9, IL24, TPX2, PDLIM4, STC1, COL6A3, CEBPD, RASSF4, TGFB3, FBN1, CD14, IDO1, CCL3L1/CCL3L3, KIF11, FADS1, SFRP2, GADD45G, TNFAIP3, CD83, MAPK11, HMOX1, ROR2, NFKBIA, ANXA1, HCK, SPHK1, MMP11, CDCA5, GTPBP4, BLM, GNG4, ACTN1, EMILIN1, PRNP, DTL, RASSF1, RUNX3, COX17, EG:10063, IRF4, UHRF1, CXCR4, RELB, ADCY3, RYR2, HYAL1, VSIG4, BCL3, CEBPB, EG:1051, CLCF1, PARP1, EMP1, WARS, PRG2, LEPREL2, PDE5A, IL1B, UBC, NCKAP1L, ELK1, MMP9, LGALS1 |
| Inflammatory Response | SOCS1, GAS6, CTSG, SERPINA3, IL6, MYLK, CXCL10, LTB4R, DUSP3, CXCL13, IFITM2, ADA, FOSL1, GNA13, AIF1, EG:11629, SERPINE1, RIPK2, ICOSLG, TIMP2, EG:21858, ADORA3, CLC, DCN, DEFA1, CACNA1C, THY1, ITGA5, HBEGF, VAMP7, CORO1C, COLEC12, NCF1, NOD2, AQP9, CTSL1, TPST1, NCF2, GPR65, CD86, GPR68, TNFAIP6, ADCYAP1, EG:11516, IL5, LCP2, COL3A1, DEK, FYN, APOE, FN1, ICAM1, POU2F2, C1R, S100A7, TGM2, NFIL3, C5AR1, CCL2, PROS1, TOP2A, CD38, TNFRSF1B, RNASE2, TNC, TYROBP, CCL23, FCGR2A, PVR, SLIT2, CERK, P2RX7, CCL11, POLD1, TSLP, IL-20, GPR183, ITGB2, SEMA3A, LY96, EG:17087, S100A9, WAS, FCER1G, CCL21, CST7, IL2RA, ADORA2A, RGS1, SOCS3, RARRES2, IL32, TLR8, CXCL1, GHSR, CD163, PLA2G7, STAB2, IL7R, CYBB, CD209, OSM, CCL8, IL8, SPP1, EG:20750, THBS1, CNP, CD97, IL24, TUB, FPR1, ICOS, JAM3, CCL3L1/CCL3L3, CD14, SBNO2, CCL18, ELMO1, GEM, ABCF1, CD83, CCL3, CTLA4, IFITM3, AOAH, SERPINB9, VNN1, CCL13, ANXA1, SPHK1, HCK, S100A8, NLRC4, PRNP, MAP4K2, NLRP3, CXCR4, PLAUR, BCL3, S100A12, NFATC4, CEBPB, EG:1051, ERG, IL-28AR1, BTK, PRG2, ASAP2, IL1B, NCKAP1L, CXCL2, ISG20, MMP9, ITGAX, LGALS1, MSN |
| Cell Cycle | CENPF, NES, CAMK1, CDC7, EG:12545, CCNB2, CDKN2C, IL6, RBL1, GPSM2, SPI1, EG:20375, MIA, CXCL10, NR4A3, BCAT1, DUSP3, CSE1L, CENPE, MAD2L2, NEK6, CAV1, CDKN3, FOSL1, AIF1, EG:11629, ANAPC11, DNMT1, TIMP2, EG:21858, PDGFRB, CENPW, TYMS, CD19, CCNE2, CDCA8, ESPL1, TOPBP1, DCN, ITGA5, HBEGF, ANLN, IER3, UBE2S, PITX2, ATF2, SKA3, RUNX1T1, BHLHE40, ZAK, GFI1, E2F1, TFRC, CYR61, SGOL1, PIP4K2A, NUDT1, UBE2C, KIF20A, DEK, APOE, FYN, TCF4, FN1, CALM1, CDT1, CUL1, NUF2, TTK, CHEK1, PDXP, YY1AP1, ARRB1, LZTS1, MXD1, GAS1, EG:14451, TOP2A, MCM10, EG:307126, KIF2C, ITGB1, CDC45, TNC, CDC6, EG:23834, SPC25, EG:100144563, PPP1R15A, MLF1, PLK1, AXL, POLD1, CDK1, RRM1, BUB1, EG:100307076, PI3, KIF15, MCM2, KIF20B, ZEB2, CENPA, NR4A1, NRIP1, GAS7, KIF23, SOCS3, GAB2, DLGAP5, LIF, PTTG1, DIAPH3, SMC4, ACVRL1, MELK, LATS2, AURKB, USH1C, BIRC5, YWHAQ, STMN1, RUNX2, PIM1, EZH2, MPHOSPH9, OSM, ZWINT, CDKN1C, ECT2, BRCA1, E2F2, KIFC1, CDC25A, IL8, KIF14, STIL, ATF3, RRAD, THBS1, CDKN2D, KIF4A, CDK6, CDK14, AURKA, MAPK12, BUB1B, IL24, TP53BP2, TPX2, DUSP1, CEBPD, PKMYT1, NDC80, KIF11, CEP55, SULF1, CDC20, GADD45G, CHRM3, CDC25B, HMOX1, NEK2, FLNA, CIT, SPHK1, NAMPT, CDCA5, BLM, PRNP, DTL, RASSF1, NUSAP1, CDC25C, TACC3, PHF13, RACGAP1, PRC1, EG:233406, PLAUR, BCL3, CEBPB, EG:1051, FOXM1, MT1A, DEGS1, PARP1, COL1A1, RASSF2, CKS1B, IL1B, BRCA2, CDKN1B, MMP9, LGALS1, IL11 |
| Cell Death | CRYAB, GAS6, HMMR, IL6, PPFIA2, SPI1, EG:20375, DYRK3, SOD2, GAL, MT2A, GNLY, CSE1L, KIF1A, NEK6, CAV1, FOSL1, SERPINE1, RIPK2, TYMS, GNE, PTGIS, EGLN2, PDIA2, ESPL1, MAPK8IP1, ATF2, SEC61G, PLEKHG2, RUNX1T1, GPR65, GFI1, TFRC, BCL2A1, ADCYAP1, EG:11516, UBE2C, CD99, APOE, DEK, ICAM1, GADD45B, CALM1, IL17RD, PRDX1, NUF2, TTK, IL17D, GLIPR1, KSR1, TGM2, DUSP5, C5AR1, ARRB1, CCL2, CAMK1G, ANTXR2, CD38, CD27, FAP, ENTPD1, EMP3, PVR, CCL11, COPB2, SEMA3A, HUS1B, WIPF1, NR4A2, SRGN, NR4A1, MAP3K8, SOCS3, LIF, PTTG1, RNASE1, TYMP, RBP1, OLR1, STMN1, PIM1, HOXC6, CCNK, JUND, CDKN1C, KIFC1, STK17B, KLF11, IL8, ATF3, THBS1, CDKN2D, PTGDS, AURKA, BUB1B, ATAD2, CDCA2, TXNRD1, TP53BP2, FOSB, ICOS, NPC1, PTPN11, DUSP1, PPP1R12B, PKMYT1, NDC80, SGPP1, IRF8, PPAT, SIRPA, PMEPA1, SULF1, NTF3, SRPX, CDC20, CCL3, CTLA4, CDC25B, FOXL2, VNN1, NEK2, MCAM, NAMPT, CDC25C, TACC3, NLRP3, CD6, PLAUR, FOXM1, STK3, ERG, BTK, PMP22, NRTN, RASSF2, BRCA2, CDKN1B, PIM2, PRUNE2, SOCS1, CTGF, SGK1, HSPB2, CDC7, EG:12545, CDKN2C, MIF4GD, SERPINB2, CXCL10, MIA, MYLK, NR4A3, PRDM1, FBXO32, TIMP2, EG:21858, PDGFRB, SFRP4, ADORA3, CD19, TOPBP1, POU4F1, MT1X, SNAI1, DEFA1, MDC1, ITGA5, HBEGF, LAG3, ANLN, UBE2S, IER3, S1PR3, CDH2, BHLHE40, ZAK, E2F1, SFRP1, ADAMTSL4, CYR61, PDCD5, SNCA, NRP1, EG:18186, AKAP12, TCF4, FN1, DEPDC1, ZFP36, UBQLN1, TWIST1, COL4A2, SOD3, CHEK1, TRPM2, UCHL1, OSCAR, NFIL3, JUN, ANXA5, TOP2A, MCM10, EG:307126, DUSP14, TNFRSF1B, FKBP5, COL18A1, FGF7, MNDA, ITGB1, CD53, CDC45, TNC, KHK, EGR1, CDC6, EG:23834, SPC25, EG:100144563, PPP1R15A, PLK1, P2RX7, CERK, SLIT2, AXL, F3, CDK1, RRM1, ITGB2, PI3, MCM2, DHCR24, ABL2, S100A9, WAS, PEA15, IL2RA, ADORA2A, CLDN3, PHLDA1, IL32, CXCL1, ACVRL1, MELK, LATS2, PLA2G7, PBK, BIRC5, YWHAQ, IL7R, G0S2, ALDH1A1, OSM, BRCA1, CDC25A, KIF14, TUBB3, FST, SPP1, EG:20750, CDK6, RGS4, IL24, STC1, PDLIM4, ITPK1, CEBPD, RASSF4, CD14, IDO1, DDAH2, SFRP2, GADD45G, GTF2F2, ERN1, TNFAIP3, COMP, NRGN, HMOX1, ROR2, SERPINB9, NFKBIA, CCL13, FLNA, ANXA1, SPHK1, HCK, S100A8, BLM, NLRC4, PRNP, RASSF1, RUNX3, IRF4, CXCR4, RELB, RYR2, LRRK2, BCL3, TCF7L1, CEBPB, EG:1051, NFKBIZ, CLCF1, PARP1, EMP1, PRG2, IL1B, EFEMP1, SNCAIP, ELK1, NCKAP1L, MMP9, MSN, LGALS1 |
| Connective Tissue Disorders | PRUNE2, SOCS1, CRYAB, CAMK1, CTSG, C1QA, CLSTN2, NRM, C1QB, IL6, ADAMTS2, CXCL10, AGPS, NR4A3, LPIN1, SOD2, CXCL13, GNLY, COL10A1, PRDM1, AIF1, EG:11629, ICOSLG, DNMT1, TIMP2, EG:21858, ZNF331, PTGIS, ADORA3, TOPBP1, POU4F1, GPR15, DEFA1, MDC1, ITGA5, TBX4, MMP2, CNTN2, UBE2G2, IFT122, NOD2, AQP9, ADAMTS6, CTSL1, TPST1, SCN1B, IL10RA, CD86, TFRC, SNCA, COL3A1, APOE, DEK, TCF4, ICAM1, GADD45B, FBXL7, FN1, ZFP36, PRDX1, TWIST1, SLC1A3, GPC4, COL4A2, IL17D, CDH11, LBR, EG:368360, COL16A1, COL5A1, C1R, VRK1, CCL2, RENBP, COL18A1, FKBP5, CD27, TNFRSF1B, RNASE2, STK19, TYROBP, FCGR2A, CCL23, COL12A1, VIM, CCL11, ITGB2, NR4A2, S100A9, NR4A1, CELF2, CCL21, CILP, IL2RA, COL15A1, SOCS3, RARRES2, MMP3, CXCL1, TLR8, CDA, BIRC5, IL7R, FCN1, G0S2, OSM, CCL8, FAM101B, STK17B, S100A10, ADAMTS4, IL8, TUBB3, COL4A1, SPP1, EG:20750, THBS1, C1S, CDKN2D, HCG27, PTGDS, CD97, ADAMTS9, PAPSS2, FOSB, ARIH1, ICOS, CLIC2, COL6A3, DUSP1, JAM3, RECQL4, HAVCR2, CCL3L1/CCL3L3, FBN1, CCL18, PPAT, NTF3, SLC1A4, RECK, ACTA2, TNFAIP3, CD83, CCL3, COMP, CTLA4, CHCHD2, CDC25B, COL1A2, ROR2, HMOX1, SH3PXD2B, NFKBIA, COL6A1, CCL13, FLNA, ANXA1, CALD1, SPHK1, ASPN, HCK, MMP11, S100A8, NAMPT, VARS, PRNP, RUNX3, COL5A2, NLRP3, COL6A2, CXCR4, CD6, HYAL1, ZNF469, VSIG4, LRRK2, TPM2, PLAUR, S100A12, CEBPB, EG:1051, SNAP25, PARP1, DNM1, COL1A1, PHF19, PDE5A, IL1B, EDNRA, COL11A1, CXCL2, MMP9, LGALS1 |
| Inflammatory Disease | DPYSL2, CRYAB, GAS6, CTSG, CLSTN2, C1QA, IL6, SPI1, EG:20375, SOD2, GNLY, MAD2L2, AIF1, EG:11629, PTGIS, TYMS, GPR15, TPM3, CNTN2, CTSL1, SCN1B, TPST1, GPR65, CD86, TFRC, APOE, DEK, ICAM1, GADD45B, PRDX1, IL17D, C1R, VRK1, C5AR1, CCL2, RENBP, CD27, RNASE2, CCL23, FCGR2A, VIM, CCL11, NR4A2, NR4A1, SOCS3, RARRES2, MMP3, IDI1, TLR8, CD163, FCN1, CCL8, STK17B, IL8, THBS1, HCG27, CDKN2D, PTGDS, PDE4B, ADAMTS9, ARIH1, FOSB, ICOS, CLIC2, DUSP1, JAM3, HAVCR2, PPAT, TPM1, EG:22003, NTF3, SLC1A4, RECK, CCL3, CTLA4, CDC25B, COL6A1, MCAM, NAMPT, VARS, NLRP3, CD6, PLAUR, SNAP25, PHF19, COL11A1, CXCL2, PRUNE2, SOCS1, CAMK1, CTGF, SGK1, NRM, C1QB, MKI67, ADAMTS2, CXCL10, CTSL2, NR4A3, LPIN1, CXCL13, COL10A1, PRDM1, OSMR, DNMT1, ICOSLG, TIMP2, EG:21858, PDGFRB, ZNF331, ADORA3, TOPBP1, POU4F1, MDC1, DEFA1, ITGA5, THY1, MMP2, UBE2G2, NOD2, AQP9, IL10RA, SNCA, COL3A1, FYN, TCF4, FBXL7, FN1, ZFP36, SLC1A3, GPC4, CDH11, S100A7, JUN, PDE3B, ANXA5, TNFRSF1B, FKBP5, MNDA, STK19, TNC, AXL, ITGB2, PI3, S100A9, CCL21, CELF2, FCER1G, CILP, IL2RA, CXCL1, CDA, BIRC5, IL7R, G0S2, IFI30, OSM, FAM101B, S100A10, ADAMTS4, PDK4, CDC25A, TUBB3, SPP1, EG:20750, FST, C1S, CPVL, CD97, TGFB3, CCL3L1/CCL3L3, FBN1, CD14, CCL18, ACTA2, TNFAIP3, CD83, COMP, CHCHD2, APOBEC3A, HMOX1, CCL13, NFKBIA, FLNA, CALD1, ANXA1, SPHK1, HCK, ASPN, PHGDH, S100A8, PRNP, RUNX3, IL13RA1, CXCR4, HYAL1, TPM2, LRRK2, VSIG4, BCL3, CEBPB, EG:1051, S100A12, PARP1, DNM1, CNN3, IL1B, EDNRA, PDE5A, MMP9, LGALS1, MSN |
