# Supplementary material for: Identification of Pro-Inflammatory Cytokines Associated with Muscle Invasive Bladder Cancer; The Roles of IL-5, IL-20, and IL-28A
Source: PLoS One. 2012 Sep 4;7(9):e40267. doi: 10.1371/journal.pone.0040267 (PMC3433484; doi:10.1371/journal.pone.0040267)
Supplement: Table S4 — Down-regulated genes in muscle invasive bladder cancer (MIBC) samples, compared to non-muscle invasive bladder cancer (NMIBC) samples. (DOCX) [file pone.0040267.s009.docx]

**Table S4. Down-regulated genes in muscle invasive bladder cancer (MIBC) samples, compared to non-muscle invasive bladder cancer (NMIBC) samples**

| Category | Molecules |
| --- | --- |
| Metabolic Disease | ALDH4A1, CLN8, IDUA, HPS6, PIK3R1, ETFDH, FABP4, MCCC1, AGA, PYGL, ARSA, CLN5, AGL, UGT1A1, FAH, AMT, MLPH, FLOT2, PCCA, FERMT1, ACY1, SLC22A5, SLC37A4, DBT, FBP1, MLYCD, IVD, ALOX5, ABCC3, SUOX, TUBG2, MCCC2 |
| Renal and Urological Disease | PPARG, PCCA, ACY1, DBT, ETFDH, MCCC1, MLYCD, IVD, MCCC2 |
| Tissue Development | SHH, GNPDA1, HES5, TBX6, NTF4, SMAD3, MSX2, FGFR3, KRT13, ADAM28, KRTAP5-9, KRT18, BCOR, NME5, HAS3, AES, ATPIF1, FLOT2, BTG2, RAB11A, KRT19, C10orf58, AGRN, ISL2, DEAF1, HOXA1, TSC1, PAX8, VAPA, SOX15, TSGA10, RPS6KA3, ABLIM1, AGGF1, TRIM27, HOXA5, NOX5, TBX3, ITGA2, PROC, NLGN4X, COL17A1, ITGA3, HERC4, CTF1, GPR56, BCAM, FABP5, WNT10A, LAMB3, ATXN10, BMP7, GATA3, GJB5, TBX1 |
| Cellular Growth and Proliferation | SHH, PRKAB1, CAPS, PIK3R1, HOXB3, MADD, HOXB7, GPRC5C, MGAT4B, DNAJA3, ITGB4, KAT6B, TNFRSF10B, NRG4, SCAMP4, DDR1, TNS4, ABCC5, CAPN1, BTG2, RXRA, ALOX5, STEAP3, TSC1, PTPN13, PSMB10, SEMA6A, GPX1 , EG:14775, GALNT2, ETFDH, PRKCZ, PLCD1, ID1, HOXA5, STAP2, PIK3R2, TNK1, PKM2, FHIT, TXNIP, MGMT, SNCG, ITGA3, ID3 , EG:15903, CIRBP, USP4, SLC22A18, FBP1, BMP7, HOXB4, CXADR, VPS28 , EG:300052, NTF4, ARL6IP5, MSH3 , EG:17686, SMAD3, UNC5B, DNAJB2, MSX2, GATA2, SLC7A4, GGA2, FGFR3, GGA1 , EG:106039, ANXA11, ENTPD5, ACSL5, CNKSR1, TRIM35, SMPD2, ACVR1B, MLL, IGFBP2, HAS3, MECOM, MDM4, FLOT2, CD40, RAP1GAP, PRKCD, POLL, ID2, RPH3AL, CDH23, RPS6KA3, ING4, IL17RA, GPC1, PPARG, SRC, TP63, DGKA, SMAD7, QSOX1, ERBB3, CTF1, GPR56, CAT, MST1R |
| Connective Tissue Development and Function | FGFR3, TBX1, HOXA1 |
| Skeletal and Muscular System Development and Function | FGFR3, ID1, ID2, TBX3, HINFP, TBX1, HOXA1, ZFHX3 |
| Tumor Morphology | SRC, ID1, ID2, TP63, CD40, PRKCD, POLL, MST1R, ID3 , EG:15903) |
| Dermatological Diseases and Conditions | TP63, WNT10A, LAMB3, DLX3, COL17A1, EDARADD, ITGB4, RXRA, HR |
| Developmental Disorder | SHH, ALDH4A1, CLN8, HPS6, IDUA, DLX3, RPS6KA3, EMX2, IGBP1, ETFDH, SRPX2, COMT, MCCC1, ITGB4, TP63, TBX3, COL17A1, AGA, PYGL, ARSA, AGL, CLN5, UGT1A1, FAH, AMT, MLPH, CTF1, GPR56, FLOT2, WNT10A, PCCA, FERMT1, LAMB3, SLC37A4, SLC22A5, ACY1, CAT, FBP1, DBT, EDARADD, MLYCD, IVD, ABCC3, ALOX5, SUOX, TBX1, TUBG2, MCCC2 |
| Genetic Disorder | SHH, CLN8, VPS28 , EG:300052, KRT7, MSH3 , EG:17686, SMAD3, PIK3R1, DLX3, IGBP1, ABCG1, ATP8B1, FGFR3, PLEK2, ANXA11, COMT, ENTPD5, KRT18, RILP, ITGB4, CAPN5, PAFAH2, AGA, ARSA, GLRX3, CLN5, UGT1A1, MLPH, TNS4, FLOT2, SCP2, FERMT1, PRKCD, ACY1, SLC22A5, SLC37A4, EDARADD, MLYCD, ALOX5, ABCC4 , EG:10257, SUOX, LGR4, HSD17B2, MCCC2, TUBG2, ALDH4A1, IDUA, HPS6, RPS6KA3, EMX2, ETFDH, AQP3, GPC1, VIPR1, EFNB1, MCCC1, JPH4, PPARG, SRC, FHIT, TP63, TBX3, MGMT, DGKA, COL17A1, FAM3B, PYGL, AGL, BAK1, FAH, AMT, RAD54B, KRT8, MGST2, PCCA, WNT10A, CIRBP, SULT1A1, UGT2B7, LAMB3, CAT, GPX2, ERN2, DBT, FBP1, BMP7, BCAS1, GATA3, ABCC3, IVD, TBX1 |
